# Single-Molecule Discrimination of Multisialylated Ganglioside Oligosaccharides Using an Engineered Nanopore

**DOI:** 10.34133/research.1286

**Published:** 2026-05-19

**Authors:** Guangda Yao, Boyang Ren, Daigui Zhu, Jianling Tan, Jingjing Hou, Yuan Ma, Zhengyu Hang, Zhuojia Xu, Zhaobing Gao, Tiehai Li, Bingqing Xia

**Affiliations:** ^1^ State Key Laboratory of Drug Research, Shanghai Institute of Materia Medica, Chinese Academy of Sciences, Shanghai 201203, China.; ^2^ Tianjin University of Traditional Chinese Medicine, Tianjin 301617, China.; ^3^ School of Chinese Materia Medica, Nanjing University of Chinese Medicine, Nanjing 210023, China.; ^4^ State Key Laboratory of Chemical Biology, Shanghai Institute of Materia Medica, Chinese Academy of Sciences, Shanghai 201203, China.; ^5^ University of Chinese Academy of Sciences, Beijing 100049, China.; ^6^ School of Pharmacy, Fudan University, Shanghai 201203, China.

## Abstract

The function of ganglioside oligosaccharides critically depends on the number, linkage, and spatial arrangement of sialic acid residues, yet direct discrimination of highly sialylated ganglioside oligosaccharides, particularly at the isomeric level, remains challenging due to their high charge density and subtle structural differences. Here, we present a nanopore-based strategy for single-molecule identification of ganglioside oligosaccharides. By engineering the sensing region of an α-hemolysin nanopore with synergistic cationic and aromatic mutations, we markedly enhance the capture of multisialylated glycans and prolong their residence within the pore. This interaction-mediated slowdown converts otherwise transient blockade events into extended trajectories containing rich molecular dynamics information. Beyond conventional blockade amplitude and dwell time descriptors, we introduce time- and spectral-domain features to characterize intraevent current fluctuations, thereby overcoming the key limitations of traditional nanopore analyses. Using this approach, we reliably discriminate 4 linkage isomers of trisialylated ganglioside glycans and accurately identify ganglioside oligosaccharides containing 3 to 5 sialic acid residues in heterogeneous mixtures. When combined with a machine-learning framework, the extracted time- and spectral-domain features enable single-level isomer identification and mixture deconvolution and remain effective even in complex biological matrices such as neural cell line and brain tissue lysates. Together, these results demonstrate that controlling analyte–pore interactions to unlock higher-order dynamical signatures enables nanopore analysis of highly sialylated ganglioside oligosaccharides, providing a general strategy for single-molecule glycan sequence elucidation in biologically relevant environments.

## Introduction

Gangliosides constitute one of the most structurally complex classes of glycoconjugates in the nervous system [1], with their biological functions predominantly encoded by the glycan moiety (named ganglioside oligosaccharides) [2]. Sialic acid residues play a central role in this encoding by imparting a high negative charge density, conformational flexibility, and diverse molecular recognition capabilities to ganglioside oligosaccharides [3–5]. A growing body of evidence indicates that subtle variations in sialylation patterns critically shape ganglioside interactions with lectins, Siglec receptors, pathogens, and immune regulators, thereby influencing neural development, synaptic plasticity, and neuroimmune homeostasis [6–9]. Highly sialylated gangliosides are dynamically regulated during neurodevelopment and are extensively remodeled in a range of neurological disorders [9]. Accordingly, elucidating ganglioside oligosaccharide structures at a level sufficient to capture these sialylation-dependent differences is essential for understanding their biological functions [10], yet achieving such structural resolution, particularly at the isomeric level, remains a major analytical challenge.

At present, structural analysis of ganglioside oligosaccharides relies primarily on liquid chromatography–mass spectrometry-based approaches [11,12]. While these methods provide valuable compositional and subclass-level information, they face intrinsic limitations in resolving highly sialylated glycans, particularly those containing 3 or more sialic acids [13,14]. Sialic acid residues are prone to in-source fragmentation and neutral loss, and distinctions among positional and linkage isomers are often inferred indirectly rather than determined experimentally. These limitations become especially pronounced in complex or mixed systems, where extensive signal overlap obscures low-abundance yet functionally relevant structures. As a result, direct determination of sialylation-dependent structural features in intact ganglioside oligosaccharides remains beyond the reach of existing analytical techniques [15].

Nanopore sensing has emerged as a powerful single-molecule analytical technology [16–19], offering label-free detection with a high temporal resolution and sensitivity to molecular size [20], charge distribution [21], and conformation [22]. Previous studies have demonstrated the feasibility of nanopore-based analysis of monosaccharides [23,24], oligosaccharides [25–27], and glycosylated biomolecules [28,29], showing that carbohydrate translocation dynamics can encode structural information. These advances point to the potential of nanopores for glycan analysis at the single-molecule level [18]. Ganglioside oligosaccharides, however, pose a substantially greater challenge. Their structural diversity is dominated by subtle differences in sialylation, which introduce only minor changes to the overall molecular size and charge. Consequently, distinct ganglioside oligosaccharide structures are expected to generate highly similar translocation signatures, leading to extensive signal overlap. How such fine structural variations can be resolved into distinguishable and reproducible electrical signals therefore remains an open and fundamental question for nanopore-based glycan analysis.

In this work, we investigate engineered nanopores for characterizing the translocation behavior of highly sialylated ganglioside oligosaccharides at the single-molecule level. By tuning analyte–pore interactions within the sensing region, we seek to modulate glycan capture and residence in a manner that increases the informational content of individual blockade events while preserving glycan integrity. This approach allows systematic examination of how sialic acid-dependent structural features are reflected in nanopore electrical signals and provides a foundation for extending nanopore sensing to structurally complex glycan systems.

## Results and Discussion

### Cationic engineering enhances the nanopore capture of multisialylated glycans

**Fig. 1.**
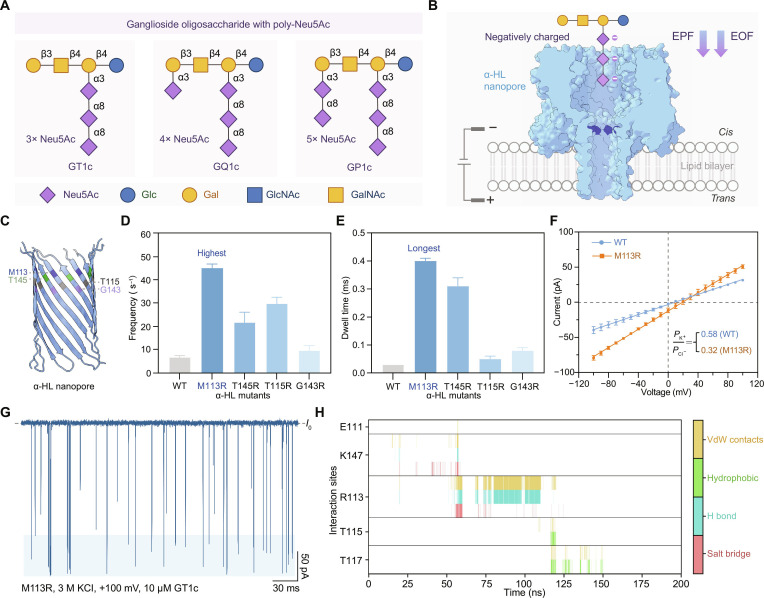
High-efficiency capture of multisialylated oligosaccharides by the α-hemolysin M113R mutant. (A) Structures of 3 representative multisialylated ganglioside oligosaccharides, GT1c, GQ1c, and GP1c, illustrated using the Symbol Nomenclature for Glycans (SNFG), in which *N*-acetylneuraminic acid (Neu5Ac), glucose (Glc), galactose (Gal), and *N*-acetylgalactosamine (GalNAc) are denoted by standard symbols. (B) Schematic illustration of the biological nanopore sensing platform, where an α-hemolysin nanopore is inserted into a lipid bilayer separating *cis* and *trans* chambers filled with symmetric electrolyte (3 M KCl, 10 mM citric acid [CA], pH 5.0) under an applied potential of +100 mV at the *trans* side, with the *cis* side grounded. (C) The positions of the sensing regions of α-hemoglobin, including M113, T145, T115R, and G143, are marked with different colors. (D) Bar chart showing the event frequency of GT1c (10 μM) events detected by wild-type α-hemolysin (WT) and its mutants carrying single arginine substitutions at different pore positions. (E) Bar chart showing the dwell time (*τ*, obtained from single-exponential fitting) of GT1c (10 μM) blockage events for the wild-type α-hemolysin (WT) and its mutants. (F) Reversal potential measurements under asymmetric electrolyte conditions (1 M KCl in the *trans* reservoir and 0.1 M KCl in the *cis* reservoir), with both solutions buffered in 10 mM CA acid at pH = 5.0, and ion selectivity calculation using the Goldman–Hodgkin–Katz equation. (G) Representative ionic current trace of GT1c sensed by the M113R nanopore. (H) Dominant interaction types occurring with frequencies greater than 0.5% during GT1c passage through residue R113. The error bars in (D) to (F) represent the standard deviation (SD) calculated from 3 independent replicates (*n* = 3).

To establish a nanopore-based identification platform for multisialylated ganglioside oligosaccharides, GT1c, GQ1c, and GP1c were selected as representative model analytes. As shown in Fig. 1A, these 3 oligosaccharides share a similar neutral glycan core but differ in the number of *N*-acetylneuraminic acid (Neu5Ac) and their modification sites on the core, thereby representing a class of ganglioside oligosaccharides that are structurally highly similar yet biologically distinct (Fig. 1A). To enable label-free nanopore measurements of multisialylated oligosaccharides, we constructed a planar lipid bilayer nanopore recording setup (Fig. 1B). In this platform, a single nanopore was reconstituted into a planar lipid bilayer separating the *cis* and *trans* chambers, permitting ionic conduction between electrolyte solutions (such as 3 M KCl, 10 mM citric acid [CA], pH = 5.0) (Fig. 1B). We employed an α-hemolysin (α-HL) nanopore (Fig. S1), which has been reported in multiple studies to support oligosaccharide identification [23,27,30]. Under an applied potential of +100 mV (*trans*-positive), the wild-type α-HL (WT) produced only a low event frequency for GT1c (6.86 s^−1^), with a dispersed distribution of normalized blockade amplitudes (Δ*I*_1_/*I*_0_) and a short dwell time (single-exponential fit: 0.03 ms), indicating that the WT did not efficiently capture the multisialylated glycans (Fig. S2A and B). The poor capture rate of α-HL (WT) for highly negatively charged glycans could be explained by the entry barrier at the pore mouth or/and the interaction network within the lumen.

Guided by the “multianionic terminal carboxylate groups (–COO^−^)” characteristic of ganglioside oligosaccharides, a site-directed mutation strategy was implemented to introduce arginine (Arg, R) at the nanopore sensing region to create a localized cationic environment near the constriction areas (Fig. 1C). This design was expected to lower the entry barrier and promote pore localization of the analyte through electrostatic attraction. To examine whether capture enhancement depends on the axial position, we constructed single-point mutants of M113R, T145R, T115R, and G143R individually (Fig. 1C and Fig. S1) and compared their event frequencies and dwell time under identical conditions (Fig. 1D and E). These mutants produced clearly distinct current traces upon GT1c measurements (Fig. S2A and B). Among them, M113R yielded the highest event frequency (45.04 s^−1^), exceeding those of T145R, T115R, and G143R and representing an over 5-fold increase relative to that of the WT (Fig. 1D). In addition, GT1c showed the longest dwell time (single-exponential fit: 0.40 ms) in the M113R mutant, corresponding to an over 12-fold increase compared to that in the WT (Fig. 1E and Table S1). Collectively, these results indicated that the M113R substitution substantially improved nanopore capture ability for multisialylated oligosaccharides and increases the temporal information content of recorded events. The nanopore ion selectivity was determined from reversal potentials under asymmetric electrolyte conditions using the Goldman–Hodgkin–Katz equation [31]. The permeability ratios (*P*_K_^+^/*P*_CI_^−^) of the WT and M113R were 0.58 ± 0.03 and 0.32 ± 0.04, respectively (Fig. 1F and Fig. S3), indicating enhanced anion selectivity in M113R, which may contribute to its increased capture rate via stronger electroosmotic flow [32,33]. Therefore, M113R mutation was considered necessary for α-HL to sense multisialylated glycans. To provide a molecular-level rationale for this enhancement after cationic engineering, we performed molecular dynamics (MD) simulations of the M113R–GT1c system. Umbrella sampling MD simulations along the pore axis indicated that as GT1c traverses the constriction region of α-HL, the carboxylate groups of Neu5Ac form a stable salt bridge with R113, accompanied by hydrogen-bonding and van der Waals interactions (Fig. 1G and Fig. S4A and B). The residue R113 also stabilizes galactose (Gal) and Neu5Ac residues through stable hydrophobic interactions and van der Waals contacts (Fig. 1G and Fig. S4C). These results offered a mechanistic basis for the experimentally observed increase in capture efficiency and dwell time.

### Double mutations reshape event kinetics and prolong residence time

**Fig. 2.**
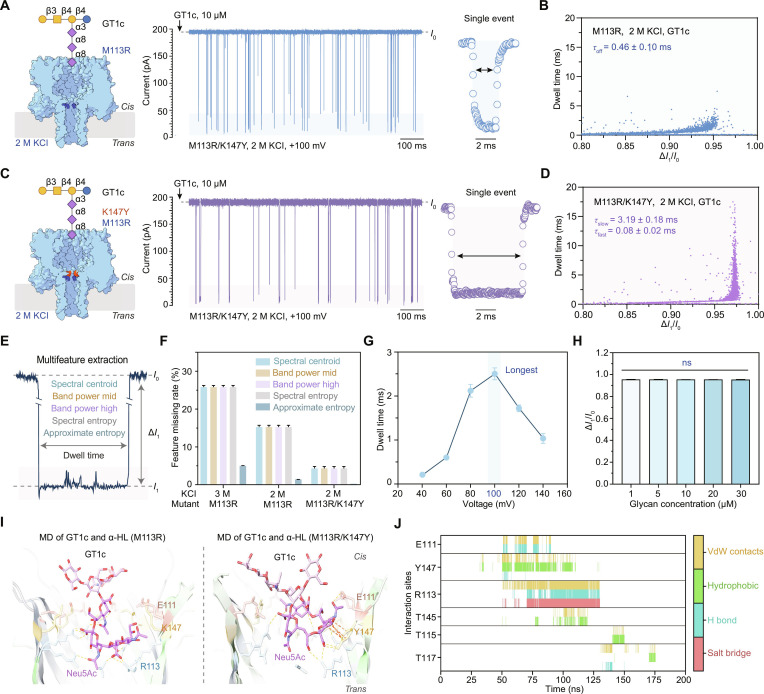
Prolonged dwell time enabling enriched feature extraction from nanopore current signals. (A) Left: Schematic illustration of GT1c sensing by the α-hemolysin (α-HL) (M113R) nanopore, nanopore insertion from the *cis* side, positively charged arginine at position 113 highlighted in deep blue, symmetric electrolyte conditions (2 M KCl, 10 mM citric acid [CA] acid, pH 5.0). Middle: Representative current trace of GT1c at 10 μM final concentration. Right: Representative single-event current blockade. (B) Scatterplot of dwell time versus normalized blockage current (Δ*I*_1_/*I*_0_) for GT1c detection under the conditions shown in (A). (C) Left: Schematic illustration of GT1c detection by the α-HL (M113R/K147Y) nanopore, tyrosine residue at position 147 highlighted in red, identical experimental conditions as in (A). The red section indicates tyrosine at position 147. Middle: Representative current trace at 10 μM GT1c. Right: Representative single-event signal. (D) Scatterplot of dwell time versus normalized blockage current (Δ*I*_1_/*I*_0_) under the conditions shown in (C). (E) Schematic depiction of extracted current signal features. (F) Bar graph of feature missing rates under 3 detection conditions, missing rate defined as the ratio of missing features to total events. (G) Voltage-dependent dwell time from +40 to +140 mV, maximum dwell time at +100 mV. (H) Blockage amplitude versus GT1c concentration gradient (1, 5, 10, 20, and 30 μM), absence of statistical significance. (I) Molecular dynamics simulation snapshots of GT1c docking within the constriction regions of α-HL (M113R) or α-HL (M113R/K147Y), labeled key residues, *N*-acetylneuraminic acid (Neu5Ac) moieties, and interaction bonds. (J) High-frequency (>0.5%) interaction profiles during GT1c passage through α-HL (M113R/K147Y), interaction types indicated. The data in (B) and (E) are presented as mean ± standard deviation (SD) (*n* = 3). The error bars in (F) to (H) represent the SD calculated from 3 independent replicates (*n* = 3).

While the single mutation M113R substantially improved capture, further analysis revealed that multisialylated glycans frequently produced near-complete blockades (Fig. 1F and Fig. S2), such that Δ*I*_1_/*I*_0_ approached saturation (Table S1). Under these circumstances, amplitude-based discrimination becomes intrinsically constrained. Consequently, subtle structural differences may no longer translate into resolvable amplitude differences. In prior studies, near-complete blockade events have been less frequently reported in nanopore-based glycan sensing systems [26,30,34,35], but this is an issue that will eventually be faced in the nanopore detection of complex glycans, especially for nonlinear glycans. Motivated by this limitation, we advanced our engineering objective from increasing capture probability to prolonging dwell time, thereby providing an extended observation window for each single-molecule event. This strategy offers a practical and effective solution to mitigating the constraints imposed by amplitude saturation. We first reduced the KCl concentration in the electrolyte from 3 to 2 M and recorded current traces for the M113R sensing of GT1c (Fig. 2A and Fig. S5). The dwell time of GT1c (*τ* = 0.46 ± 0.10 ms) under 2 M KCl was longer than that under 3 M KCl (Fig. 2B), which could be caused by decreased electroosmotic flow in 2 M KCl. Building on this result, we sought to introduce an additional interaction site within the sensing region. We therefore incorporated a tyrosine residue at the position adjacent to M113R, generating the M113R/K147Y double mutant (Fig. 2C). The aromatic ring of tyrosine was expected to support additional CH–π and/or hydrophobic contacts with the glycan, potentially stabilizing transient binding configurations [36]. Under the same electrolyte conditions (2 M KCl, pH 5.0), GT1c events recorded with M113R/K147Y (Fig. 2C) exhibited a markedly prolonged residence time, with *τ*_slow_ = 3.19 ± 0.18 ms and *τ*_fast_ = 0.08 ± 0.02 ms (Fig. 2D and Fig. S6). These data indicated that the double mutation substantially remodels event kinetics while preserving efficient capture.

Importantly, prolonging residence time enabled more extensive sampling of intraevent current fluctuations, which provided an opportunity to move beyond conventional signal parameters such as blockade amplitude and dwell time. The long-residence events enabled by the double mutant provide a sufficient time window to estimate the energy distribution and complexity of the current fluctuations with improved stability. Accordingly, we introduced time- and spectral-domain descriptors as a complementary event representation, including spectral centroid, band power in defined frequency ranges, spectral entropy, and approximate entropy (Fig. 2E), which were extracted using Python based on the calculation formula and physical meaning of each feature shown in Table S2 and Fig. S7. These descriptors compactly encoded the distribution and complexity of intraevent fluctuations and were therefore expected to be more sensitive to interaction-kinetic differences between closely related glycans. Comparison across conditions showed that multiple key spectral-domain features exhibited a substantially reduced missing rate when sensed by M113R/K147Y in 2 M KCl (Fig. 2F). Notably, the missing rate of approximate entropy decreased to nearly 0 (Fig. 2F), indicating that intraevent fluctuations had become a stable, quantifiable information source. Over the voltage range from +40 to +140 mV (20 mV per step), the dwell time reached a maximum at +100 mV (Fig. 2G, Fig. S8, and Table S3). Therefore, +100 mV was selected as the operating voltage for subsequent measurements. The results of glycan concentration-dependence measurements showed that increasing the analyte concentration did not significantly alter Δ*I*_1_/*I*_0_ (Fig. 2H, Fig. S9, and Table S4), which suggested that the events were single-molecule level.

To further elucidate how the K147Y substitution enhanced residence time, we performed MD simulations of GT1c passage through the M113R/K147Y nanopore using umbrella sampling under the NPT ensemble (Fig. 2I and Fig. S8). Compared with the single mutant M113R, the introduction of Y147 provided additional stabilizing interactions beyond the R113–COO^−^ salt bridge (Fig. 2I and Table S5). In addition, hydrogen bonds also played an important role in the interaction between α-HL and the analytes such as peptides [37–40]. In our results, the sialic acid pyranose ring engaged in a CH–π interaction with the aromatic ring of Y147, accompanied by a more extensive network of hydrophobic and van der Waals contacts involving neighboring residues (Fig. 2J and Fig. S10). These interaction features were consistent with the experimentally observed prolongation of residence time and improved event uniformity, supporting a cooperative role of the double mutation in modulating both capture and retention. In the additional constant-velocity steered molecular dynamics (SMD) simulations under the NVT ensemble, interaction results showed less complex interaction networks in the M113R/K147Y system. However, the glycan maintained interactions with α-HL over the course of GT1c translocating, including stable hydrogen-bonding interactions with R113 and stable hydrophobic interactions with Y147, with sialic acid playing a pronounced role, consistent with these obtained under the NPT ensemble using umbrella sampling (Fig. S11). Interaction features were consistently observed although with changed initial position of glycan (Fig. S12). Analysis of GT1c conformational dynamics further revealed small fluctuations in root mean square deviation, root mean square fluctuation, and radius of gyration during passage, particularly in the M113R/K147Y system (Fig. S13), indicating that GT1c maintains a relatively stable conformation within the pore. Such conformational stability, together with the strengthened analyte–pore interaction network, is likely to restrict rotational and translational freedom of the glycan during passage, providing a plausible explanation for the pronounced and consistent current blockade observed experimentally.

### Discrimination of multisialylated ganglioside oligosaccharide isomers

**Fig. 3.**
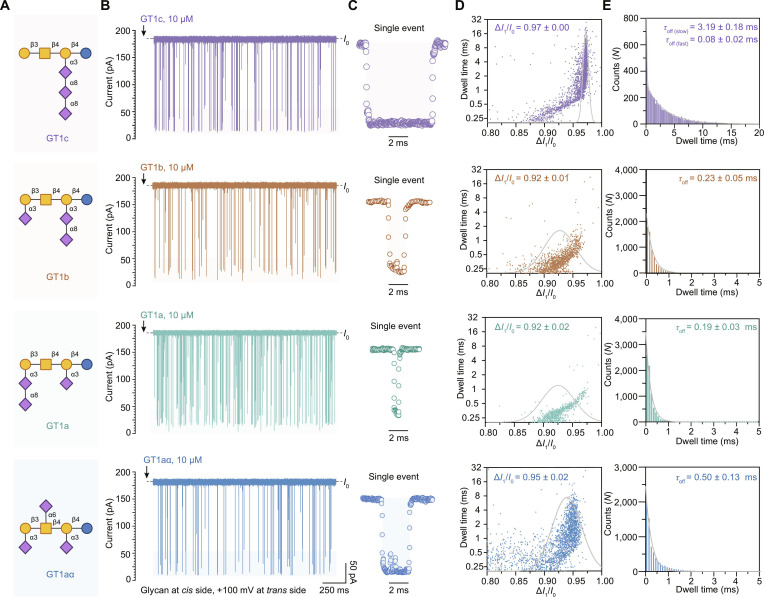
Identification of ganglioside oligosaccharide isomers using the α-hemolysin (α-HL) (M113R/K147Y) nanopore. (A) Structural representations of 4 ganglioside oligosaccharide isomers illustrated using the Symbol Nomenclature for Glycans (SNFG), isomeric differences arising from distinct spatial distributions of 3 *N*-acetylneuraminic acid (Neu5Ac) residues. (B) Representative ionic current traces generated by the 4 ganglioside oligosaccharide isomers detected by the α-HL (M113R/K147Y) nanopore. (C) Representative single-event current blockade signals corresponding to the traces shown in (B). (D) Two-dimensional scatterplots of dwell time versus normalized blockage current (Δ*I*_1_/*I*_0_) generated by the 4 ganglioside oligosaccharide isomers, with fitted curves of blockage current (Δ*I*_1_/*I*_0_) superimposed. (E) Fitted curves of dwell time for the 4 ganglioside oligosaccharide isomers. All nanopore experiments were performed in symmetric electrolyte solution (2 M KCl, 10 mM citric acid [CA] acid, pH = 5.0), with the *cis* side grounded and a potential of +100 mV applied to the *trans* side. Each glycan was added to the *cis* side to reach a final concentration of 10 μM. *n* ≥ 3.

To evaluate the analytical performance of the α-HL (M113R/K147Y) nanopore sensing system, we systematically investigated 4 trisialylated ganglioside isomers, namely, GT1c, GT1a, GT1b, and GT1aα, because current methods such as mass spectrometry face difficulties in directly resolving the complete structure of highly sialylated and isomeric ganglioside oligosaccharides, especially in complex mixtures [13–15]. We synthesized and purified these 4 glycan isomers to obtain structurally well-defined standards. As shown in Fig. 3A, despite sharing an identical monosaccharide composition consisting of glucose, Gal, *N*-acetylgalactosamine, and Neu5Ac, these isomers are distinguished by the specific linkage positions and spatial distribution of their 3 Neu5Ac along the neutral glycan core. Under the optimized and unified experimental conditions (2 M KCl, 10 mM CA acid, pH 5.0, +100 mV applied to the *trans* side), each glycan was efficiently captured, generating reproducible current blockade events (Fig. 3B). The detailed inspection of the raw current traces (Fig. 3B and Fig. S14) and representative single-event diagrams (Fig. 3C) revealed that although Δ*I*_1_/*I*_0_ remained within a similar range, the isomers exhibited distinct intraevent current fluctuations. Specifically, the blockade events of GT1a and GT1b exhibited shorter and comparable residence times, whereas the events of GT1aα and GT1c showed substantially longer residence times (Fig. 3C). Notably, the events of GT1aα were characterized by more frequent spikes and pronounced fluctuations within individual events (Fig. 3C), suggesting that its specific Neu5Ac arrangement undergoes a more complex set of conformational transitions during pore occupancy. The event dynamics were also reflected in the 2-dimensional scatterplots of Δ*I*_1_/*I*_0_ versus dwell time (Fig. 3D). The events group of GT1a and GT1b clustered in the shorter-time region. In contrast, more events of GT1aα and GT1c distributed in the longer-time region, indicating a higher affinity or increased steric hindrance within the pore. Statistical analyses of the blockade amplitudes and durations were performed for each glycan (Fig. 3D and E). The results show that the Gaussian-fitted Δ*I*_1_/*I*_0_ values of GT1c, GT1b, GT1a, and GT1aα were 0.97 ± 0.00, 0.92 ± 0.01, 0.92 ± 0.02, and 0.95 ± 0.02, respectively. The dwell time of GT1c followed a biexponential distribution, with *τ*_slow_ = 3.19 ± 0.18 ms and *τ*_fast_ = 0.08 ± 0.02 ms. Such behavior was typically attributed to the presence of 2 distinct interaction states within the nanopore (e.g., weak vs. strong binding) or to different entry orientations/conformations corresponding to distinct pathways. The exponential-fitted dwell times of GT1b, GT1a, and GT1aα were 0.23 ± 0.05, 0.19 ± 0.03, and 0.50 ± 0.13 ms, respectively. These observations indicated that even when isomeric differences are confined to Neu5Ac linkage patterns, the α-HL (M113R/K147Y) nanopore could transduce such differences into measurable dynamics. The ability to discriminate between these specific trisialylated structures is biologically important, as the isomers often serve as critical markers for neurodevelopmental stages and pathological conditions. For instance, GT1b is a major ganglioside component of the adult mammalian brain and contributes to neuronal stability [41], whereas alterations in the relative abundance of GT1b and its closely related isomer GT1a have been reported to correlate with certain tumors and inflammatory conditions [41,42].

### Machine-learning-assisted automated identification of a ganglioside oligosaccharide mixture

**Fig. 4.**
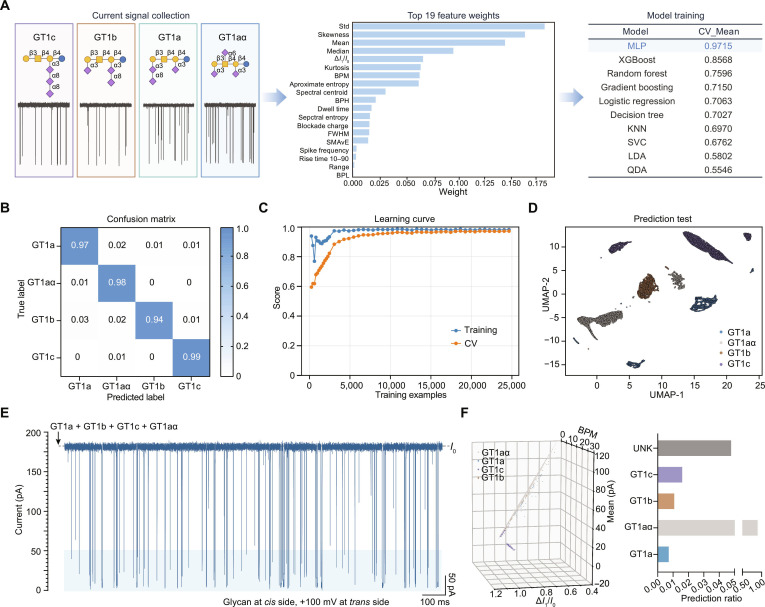
Automatic identification of ganglioside oligosaccharide isomers using machine learning. (A) Machine-learning workflow for isomer classification based on nanopore current signals; event collection from GT1c, GT1b, GT1a, and GT1aα current traces; construction of a multiclass event database; extraction of the top 12 informative event features from the top 19 features for feature matrix generation; random selection of approximately 10,000 samples per class to form a labeled dataset; dataset partitioning into training (70%), validation (15%), and testing (15%) subsets; validation accuracy defined as the fraction of correctly classified events; comparative evaluation of 10 machine-learning models; highest classification accuracy achieved by the multilayer perceptron (MLP) model with an accuracy score of 0.9715. (B) Confusion matrix derived from predictions on the independent testing set. (C) Learning curve illustrating model performance as a function of training set size. (D) Dimensionality-reduced scatterplot of testing set data, visualization of class separability in feature space. (E) Representative ionic current traces of the 4 ganglioside oligosaccharide isomers detected by the α-hemolysin (α-HL) (M113R/K147Y) nanopore. (F) Three-dimensional feature-space distribution plot and corresponding class count distribution after mixture prediction; feature axes defined as band power middle (BPM; *x*-axis), mean (*y*-axis), and normalized blockage current (Δ*I*_1_/*I*_0_; *z*-axis). All nanopore experiments were conducted in a symmetric electrolyte solution (3 M KCl, 10 mM citrate, pH = 5.0), with the *cis* side grounded and a potential of +100 mV applied to the *trans* side. For the mixture, each glycan was added to the *cis* side to reach a final concentration of 5 μM. *n* ≥ 3.

Although the α-HL (M113R/K147Y) nanopore revealed systematic dynamical differences among glycans, event distributions still overlapped to some extent, making robust classification difficult using only one or a few manually selected parameters. We therefore constructed a machine-learning workflow that integrates time-domain statistical features and spectral-domain features to enable event-level identification of glycan isomers. Using a custom script, we extracted a feature set from raw current traces for each event and ranked features by their standardized one-way analysis of variance *F* values (Fig. 4A and Fig. S15). Then, the top 12 most informative features were selected from the 19 features, including current standard deviation, skewness, mean current, and median current. (Fig. 4A), for model training. We evaluated multiple classification algorithms using 10-fold cross-validation and found that a multilayer perceptron (MLP) achieved the best performance, with an average score of 0.9715 (Fig. 4A and B and Fig. S16). Learning-curve analysis indicated that both training and cross-validation scores stabilized and approached 1 as the training set increased, suggesting stable generalization with sufficient data (Fig. 4C). On an independent test set, the normalized confusion matrix showed high recall values ranging from 94% to 99% across the 4 GT1 isomers. Uniform manifold approximation and projection (UMAP) visualization further revealed well-separated clusters in the reduced feature space, supporting the discriminative capacity of the selected features (Fig. 4D). We next applied the trained MLP model to a mixture containing all 4 ganglioside species. Representative continuous current traces were acquired (Fig. 4E), and individual events were fed into the trained classifier for event-by-event prediction. All 4 constituents in the mixture were successfully detected and classified. A 3-dimensional feature-space visualization showed an overall tendency toward spatial separation among the 4 isomers, with each class occupying a preferential region, although partial overlap and differences in event density were observed (Fig. 4F). UMAP projection of the predicted mixture events (Fig. S17) reproduced the cluster structure observed for the corresponding pure standards, providing further evidence that the approach supports event-level mixture deconvolution and single-molecule identity assignment.

### Multiclass ganglioside identification in complex biological matrices

**Fig. 5.**
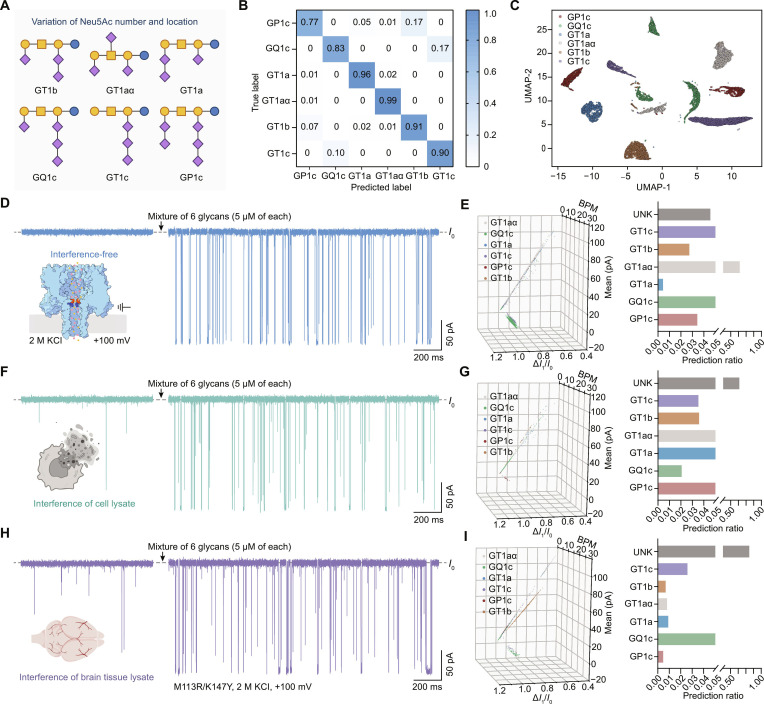
Identification of ganglioside oligosaccharide mixtures in complex biological backgrounds. (A) Structures of 6 ganglioside oligosaccharides (GT1b, GT1a, GT1aα, GQ1c, GT1c, and GP1c) in Symbol Nomenclature for Glycans (SNFG). (B) Confusion matrix generated using the testing set data for the 6 glycans. (C) Dimensionality-reduced scatterplot of the testing set data for the 6 glycans. (D) Representative current trace of the 6-ganglioside mixture via M113R/K147Y without interference. (E) 3-dimensional (3D) feature-space distribution plot and predicted proportions of the 6-component ganglioside oligosaccharide mixture classified by the multilayer perceptron (MLP) machine-learning model. (F) Representative current trace of the 6-ganglioside glycans mixture in SH-SY5Y cell lysate. (G) 3D feature-space distribution plot and predicted proportions of the mixture in (F). (H) Representative current trace of the 6-ganglioside mixture in mouse brain tissue lysate. (I) 3D feature-space distribution plot and predicted proportions of the mixture in (H). All nanopore experiments were conducted in a symmetric electrolyte solution (3 M KCl, 10 mM citric acid [CA], pH 5.0), with the *cis* side grounded and a potential of +100 mV applied to the *trans* side. For the mixture, each glycan was added to the *cis* side to reach a final concentration of 5 μM. *n* ≥ 3.

Building on the high-accuracy classification of 4 trisialylated isomers, we expanded the framework to 6 ganglioside oligosaccharide standards (GT1b, GT1aα, GT1a, GQ1c, GT1c, and GP1c) to evaluate performance under increased structural complexity (Fig. 5A). Compared with the 4-class classification task, the 6-class glycans introduced increased structural diversity, encompassing not only distinct Neu5Ac spatial distributions but also variations in Neu5Ac number (3 to 5 residues) (Fig. 5A). This expanded structural heterogeneity posed a more stringent challenge for both kinetic and spectral discrimination. Using the same nanopore mutant (M113R/K147Y) and identical recording conditions, current blockade signals of GQ1c and GP1c were acquired (Fig. S18). The event feature extraction pipeline was kept unchanged. The top 12 current features of GQ1c and GP1c were then combined with those of GT1b, GT1aα, GT1a, and GT1c to construct a 6-glycan dataset for training the MLP model (Fig. S19). The resulting 6-class MLP model exhibited stable performance (Fig. S19), achieving an overall prediction accuracy of 89.33%. Further analysis revealed that misclassifications were predominantly confined to GT1b versus GP1c and GQ1c versus GT1c, whereas glycan classes with isomers in Neu5Ac distribution displayed clearer decision boundaries (Fig. 5B). Consistently, UMAP projections resolved 6 well-separated clusters, indicating that the extracted time-domain and spectral-domain features effectively capture event-level kinetic and spectral signatures associated with glycan structural variations (Fig. 5C). A mixture of the 6 glycans, each at a concentration of 5 μM, was measured to obtain representative current traces (Fig. 5D and Fig. S20). Event-level features were subsequently extracted from the current traces and fed into the trained MLP model. All of the 6 glycans were successfully identified (Fig. 5E and Fig. S21).

We then challenged the nanopore system with complex biological matrices at both the cellular and tissue levels to evaluate its robustness under realistic sample backgrounds. Given that multisialylated ganglioside oligosaccharides are abundantly expressed in SH-SY5Y cells (a human neuroblastoma cell line) [43], as well as in brain tissue [2], SH-SY5Y cell lysates and mouse brain tissue lysates were prepared as representative biological matrices for subsequent nanopore measurements. Relative to the lysate-free buffer control of equal volume, the addition of cell lysate (5% v/v, 0.42 mg ml^−1^) or brain tissue lysate (5% v/v, 4.29 mg ml^−1^) to the M113R/K147Y nanopore generated abundant nonspecific transient events, characterized by increased noise, collision-like currents, and unstable-amplitude blockades (Fig. S22). Therefore, lysates at this concentration and volume were considered effective interfering backgrounds. While maintaining a constant total concentration and composition of the 6-component ganglioside oligosaccharide mixture, either cell lysate (5% v/v) or brain tissue lysate (5% v/v) was added, and the resulting current signals were analyzed using the trained MLP model (Fig. 5F to I and Fig. S23). Notably, even under conditions with pronounced biological noise (Fig. 5F and H), the machine-learning workflow remained effective and identified the 6 glycans successfully (Fig. 5G and I and Figs. S24 and S25). In the 3-dimensional feature-space distribution, the 6 glycans exhibited distinguishable clustering trends that enabled identification of the mixture components, although differences in cluster density were apparent, reflecting unequal event counts for certain species (Fig. 5G and I). Compared with the 6-glycan mixture without lysates, the introduction of cell lysate or brain tissue lysate led to elevated background signals, which were predominantly classified into the “unknown” (UNK) category. Together, these results suggest that the nanopore–machine learning strategy is tolerant of complex biological matrices and can support multicomponent identification and qualitative profiling of target ganglioside oligosaccharide in heterogeneous samples.

## Conclusion

In this study, we established an interaction-engineered nanopore strategy for the structural identification of multisialylated ganglioside oligosaccharides at the single-molecule level. By introducing synergistic cationic and aromatic residues (M113R/K147Y) into the sensing region of α-HL, we modulated electrostatic and CH–π interactions between the pore and highly sialylated glycans. These engineered interactions substantially prolonged the glycan residence time and reshaped passage kinetics, converting otherwise transient blockade events into extended electrical trajectories amenable to detailed fluctuation analysis. The improved structural resolvability observed here arose not simply from stronger analyte capture, but from enhanced temporal sampling. When the blockage events are of limited duration, the number of statistically independent current measurements is inherently limited, and structurally distinct analytes may yield overlapping blockade distributions. Extending residence time increases the effective observation window, allowing current fluctuations associated with molecular orientation, conformational dynamics, and interaction kinetics within the sensing region to be sampled with greater statistical robustness. This expanded sampling enables the extraction of higher-order descriptors in both the time domain (e.g., dwell time distributions and intrablockade dynamics) and the spectral domain (e.g., characteristic components of interaction-modulated fluctuations), which provides additional signal parameters for glycan electrical signal analysis, thereby reducing ambiguity between structurally similar glycans such as isomers. Our strategy can also guide nanopore design for distinguishing complex glycans with similar size exclusion effects, including the multisialylated glycan isomers used in this study.

Importantly, this work reframed nanopore sensing of glycans from amplitude-based discrimination toward dynamics-informed structural decoding. Rather than relying solely on instantaneous blockade levels, structural information was encoded in interaction-mediated current fluctuations that reflect analyte–pore coupling within a confined nanoscale environment. Such a strategy may be particularly valuable for highly charged and conformationally flexible glycans, where subtle variations in linkage and sialylation pattern do not necessarily translate into large static conductance differences.

Mass spectrometry remains the mainstream technology for glycomics analysis owing to its high sensitivity and sample compatibility. However, discrimination of highly sialylated ganglioside isomers often depends on chromatographic separation, derivatization strategies, or multistage fragmentation workflows. The nanopore approach described here is not intended to replace mass spectrometry, but rather to complement it by providing a label-free, single-molecule electrical readout that directly reflects interaction dynamics. The time- and spectral-domain signatures identified in this study constitute an orthogonal source of structural information that may assist, validate, or refine conventional glycomic analyses, particularly in cases where isomeric complexity or limited sample amounts pose challenges.

Several limitations of the current approach should be acknowledged. The effectiveness of this strategy depends on sufficiently strong and structurally informative interactions between the analyte and the engineered sensing region. In multicomponent systems, competitive pore occupancy was observed, with analytes exhibiting stronger interactions preferentially dominating blockage events. This behavior influences event statistics and currently constrains rigorous quantitative analysis of complex mixtures. These effects reflect intrinsic single-molecule transport dynamics rather than a limitation of structural discrimination itself. Future efforts involving more refined control over pore chemistry, tunable interaction strength, or advanced probabilistic data analysis frameworks may help mitigate these constraints and improve quantitative robustness.

Beyond methodological considerations, the ability to discriminate multisialylated ganglioside glycans in complex biological matrices suggests broader biological relevance. Altered ganglioside sialylation patterns are implicated in neurodevelopment, neurodegenerative disorders, and tumor-associated glycan remodeling. A sensing strategy that operates without labeling and tolerates crude lysates, as demonstrated here with neural cell line and brain tissue samples, may provide a foundation for future glycan profiling platforms requiring minimal sample amounts. While substantial technical development remains necessary before translational implementation, the present results illustrate the feasibility of probing subtle glycan structural variations in biologically relevant environments through electrical single-molecule measurements. Taken together, this work establishes a generalizable strategy for leveraging controlled analyte–pore interactions to unlock higher-order dynamical signatures and access structural information that is otherwise obscured in conventional nanopore analyses. By integrating interaction engineering with time- and spectral-domain feature extraction, we expand the scope of nanopore sensing toward the single-molecule analysis of structurally complex glycans in biologically relevant contexts.

## Materials and Methods

### Reagents and materials

All glycan compounds used in this study were synthesized in the Li Lab. The synthesis and characterization information of ganglioside oligosaccharides are detailed in the Supplementary Materials [44,45]. Other main reagents and materials were obtained from commercial suppliers and used without further purification unless otherwise specified. These included potassium chloride (Sigma-Aldrich, USA), 1,2-diphytanoyl-*sn*-glycero-3-phosphocholine (DPhPC; Avanti Polar Lipids, USA), chloroform (Sinopharm Chemical Reagent Co., Ltd., China), CA (Sigma, USA) decane (Sigma-Aldrich, USA), sodium dodecyl sulfate (Yeasen, China), PrimeSTAR Max DNA polymerase (Takara Biomedical Technology (Beijing) Co., Ltd., China), agarose (Yeasen, China), ampicillin sodium salt (Yeasen, China), yeast extract and tryptone (Thermo Fisher Scientific, USA), agar (Sinopharm Chemical Reagent Co., Ltd., China), E.Z.N.A. Plasmid DNA Mini Kit I (Omega Bio-Tek, USA), isopropyl-β-d-1-thiogalactopyranoside (IPTG; Yeasen, China), dithiothreitol (Yeasen, China), HisSep Ni-NTA agarose resin 6FF (Yeasen, China), imidazole (Yeasen, China), PageRuler prestained protein ladder (Thermo, USA), sodium dodecyl sulfate–polyacrylamide gel electrophoresis (SDS-PAGE) running buffer powder (Yeasen, China), Coomassie Brilliant Blue G-250 (Yeasen, China), *Escherichia coli* BL21 (DE3) pLysS chemically competent cells (TransGen Biotech, China), and *E. coli* Trans5α chemically competent cells (TransGen Biotech, China). The electrolyte buffer (2 or 3 M KCl, 10 mM CA, pH 5.0) was prepared using double-distilled water. Stock solutions of GT1a, GT1b, GT1c, GT1aα, GQ1c, and GP1c were prepared in double-distilled water at 20 mM concentration. Whole brains were harvested from healthy C57BL/6 mice and processed immediately. All animal procedures were approved by the Institutional Animal Care and Use Committee (Protocol No. 2025-07-GZB-25). The SH-SY5Y cells were purchased from the National Collection of Authenticated Cell Cultures (Shanghai, China) and cultured using Dulbecco’s modified Eagle medium (containing 10% fetal bovine serum) (Thermo, USA). Brain tissues or SH-SY5Y cells were suspended in lysis buffer and homogenized via mechanical grinding, followed by ultrasonication. The mixture was centrifuged at 2,000 × *g* for 15 min at 4 °C. The resulting supernatant was collected and ultrafiltered using a 3-kDa centrifugal filter (Millipore, USA). Protein concentrations were determined using a bicinchoninic acid protein assay kit (Thermo, USA).

### Preparation of α-HL mutants

The preparation of α-HL and its mutants followed our previously reported protocols [23]. Briefly, mutant variants (M113R, T145R, T115R, G143R, and M113R/K147Y) were generated via site-directed mutagenesis using the WT α-HL gene as a template. The mutant genes were cloned into pEASY expression vectors with a C-terminal 6×His tag, verified by DNA sequencing, and transformed into *E. coli* BL21(DE3) pLysS. The transformants were cultured in 1 l of Luria–Bertani medium supplemented with ampicillin (100 μg/ml). When the culture reached an OD_600_ of 0.6, protein expression was induced with 0.5 mM IPTG at 16 °C for 16 h. After cell disruption via high-pressure homogenization, the soluble His-tagged proteins were purified using Ni-NTA affinity chromatography and eluted with 300 mM imidazole. The purity and molecular weight of the purified proteins were assessed by 12% SDS-PAGE followed by Coomassie Brilliant Blue staining.

### Single-channel recordings

Single-channel recordings were conducted following a previously established protocol [30]. Briefly, a planar lipid bilayer was formed from DPhPC (dissolved in decane) across a 50-μm aperture in a polytetrafluoroethylene film, which partitioned the setup into *cis* and *trans* chambers. Each chamber was filled with 260 μl of electrolyte solution such as 2 M KCl (10 mM CA, pH 5.0), with a bias voltage applied to the *trans* side. A single α-HL nanopore or its mutant was inserted into the bilayer from the *cis* side, confirmed by a stable open-pore current. The analytes were premixed and then introduced into the *cis* chamber, followed by thorough stirring to ensure homogeneity. The ionic current signals were acquired using a Cube-D2 instrument (https://zenodo.org/records/11609574), low-pass filtered at 5 kHz, and sampled at 50 kHz.

### Data preprocessing and feature extraction

Raw nanopore recordings consist of continuous ionic current traces containing transient, discretely distributed blockade events. In this study, the event start times and end times were extracted using Clampfit (V10.6). Event-level feature extraction was performed using a custom Python pipeline. For each event, the event segment was defined as the current time series between its extracted start and end indices. The open-pore baseline current (*I*_0_) was estimated from the immediately preceding open-pore interval, defined as the time window from the end of the previous event to the beginning of the current event (for the first event, from the beginning of the selected analysis window to the event onset). To ensure data quality, events were automatically excluded if the preceding baseline segment contained insufficient samples or if the event duration was shorter than a predefined cutoff.

For each retained event, we computed a multidimensional feature vector capturing time-domain, spectral-domain, and morphological characteristics. Time-domain features included dwell time, descriptive statistics of the current amplitude (mean, median [Med], standard deviation [Std], skewness [Skew], and kurtosis [Kurt]), and the normalized blockade amplitude (Δ*I*_1_/*I*_0_). Morphological features were used to characterize event kinetics and included blockade charge (integrated blockade area), rise time (10% to 90%), fall time (90% to 10%), and full width at half maximum. Spectral-domain features were derived from the power spectral density estimated using the Welch method, including spectral centroid and relative power within low-, mid-, and high-frequency bands. In addition, approximate entropy and spectral entropy were calculated to quantify signal complexity. To mitigate the impact of transient fluctuations within events, we implemented a robust spike-detection procedure based on the median absolute deviation to identify and flag short-lived intraevent perturbations.

### Machine learning

A supervised learning framework was implemented in Python to classify ganglioside isomers. The preprocessing pipeline consisted of (a) imputing missing values using a median-based SimpleImputer fitted on the training set, with additional missingness indicator variables and (b) standardizing features to zero mean and unit variance using StandardScaler. (c) To improve training data quality, HDBSCAN clustering was applied to remove noise points and outliers from the training set. We evaluated multiple widely used classifiers, including MLP, random forest, XGBoost, support vector classifier, and linear discriminant analysis. Model performance was assessed using stratified cross-validation with 10 folds repeated 5 times, and metrics were reported on the validation set, including accuracy, macro-averaged F1 score (macro-F1), and balanced accuracy. The best-performing model was selected based on validation macro-F1 and saved as a serialized model bundle. The MLP classifier achieved the strongest validation performance and was therefore selected as the final model. The full preprocessing pipeline (imputer, scaler, and label encoder) was stored together with the trained model to ensure consistent inference.

To identify in-distribution samples and suppress anomalous events, a class-conditional kernel density estimation (KDE) gate was constructed in the standardized feature space. For each class, a Gaussian KDE was fitted on the training set; an acceptance threshold was defined as the fifth percentile of the training log-likelihood distribution and subsequently calibrated on the validation set to achieve a target per-class coverage of 70%. During inference, the KDE corresponding to the predicted class was applied; samples with log-likelihood below the threshold were labeled as “not accepted”, and model performance was additionally evaluated on the accepted-only subset.

All data processing, feature engineering, statistical testing, model training, and visualization were executed in the Python environment. The implementation relied primarily on the following libraries: NumPy, Pandas, SciPy, scikit-learn, umap-learn, hdbscan, xgboost, and Matplotlib/Seaborn.

### MD simulations and analysis

To elucidate the mechanism underlying the observed current perturbations, we performed all-atom MD simulations. The nanopore models of M113R and M113R/K147Y were built using the defined structures of *Staphylococcus aureus* α-HL pore as temple (Protein Data Bank code: 7AHL) by SWISS-MODEL [46]. The protonation states of titratable residues at pH 5.0 were assigned based on the pKa values predicted by H++, PropKa, DeepKa, and KaML-ESM (Table S6) [47–50]. The glycan was built by Carbohydrate Structure Database/Symbol Nomenclature for Glycans Structure Editor [51]. To build a simulation system, we place the molecular model into a 1,2-dilauroyl-*sn*-glycero-3-phosphocholine lipid bilayer. The lipid-embedded nanopore model was solvated in a periodic boundary condition box (115 Å × 115 Å × 233 Å) filled with TI3P water molecules and 2 M KCl using CHARMM-GUI [52]. On the basis of the CHARMM36m all-atom force field [53–55], MD simulations were conducted using the graphics-processing-unit-accelerated GROMACS software package (version 2025.2) [56]. Systems initially underwent a 5,000-step minimization with positional restraints on the protein backbone, side chains, lipids, and dihedral angle [57]. Subsequently, a 6-step equilibration process protocol generated by CHARMM-GUI was carried out with progressively reduced restraints under NVT and NPT conditions. During the first 3 equilibration steps, the system was gradually heated to 298.15 K with a 1-fs timestep using stochastic velocity rescaling with protein [58], membrane, and ion-water groups treated independently. Pressure was maintained at 1 atm by semi-isotropic application of a stochastic cell rescaling within 375,000 steps [59]. The last 3 steps maintained these conditions for 750,000 steps with a 2-fs timestep. Throughout these 6 steps, positional restraints on protein residues, glycan, and lipid molecules were progressively relaxed [57]. Following equilibration, SMD was performed in the NPT ensemble at temperature of 298.15 K using stochastic velocity rescaling and a pressure of 1 atm by semi-isotropic application of a stochastic cell rescaling following commonly used umbrella sampling workflows and the parameter settings adopted from established GROMACS/CHARMM-GUI-based protocols. The LINCS algorithm was employed to restrain bonds involving hydrogen atoms, which allowed a 2-fs timestep. Long-range electrostatic interactions were calculated using the particle mesh Ewald method, while short-range electrostatic and van der Waals interactions were treated with a 12-Å cutoff and gradually switched between 12 and 10 Å. The transmembrane bias *V* = 100 mV was induced by applying a constant electric field normal to the lipid bilayer. The glycan was initially positioned along the pore *z*-axis in the *cis* chamber of the nanopore [35], providing sufficient space for pulling simulations along the *z*-axis. During SMD, pulling potentials were applied to Neu5Ac using the lipid bilayer as an immobile reference for the pulling simulations. The glycan was passed through nanopore along the pore axis over 3 ns at a constant velocity of 5 nm·ns^−1^, with a harmonic force constant during umbrella pulling of 1,000 kJ·mol^−1^·nm^−2^. From this trajectory, snapshots were taken to generate the starting configurations for the umbrella sampling windows. The windows were centered at approximately 0.2-nm intervals along the *z*-axis [60,61]. A total of 20 individual umbrella sampling windows centered around residue 113 were each simulated for 10 ns, after 100 ps of NPT equilibration, using the same methodology described above, for a total simulation time of 200 ns of MD simulations. The windows were centered at about 0.2-nm intervals along the *z*-axis.

In addition, additional umbrella sampling simulations were performed to examine the sensitivity of the results to the initial configuration. Specifically, for both the M113R and M113R/K147Y systems, the initial axial position of the glycan was varied while kept approximately aligned with the pore axis. To more stringently test the robustness of the analysis against lateral displacement, additional simulations were conducted for the M113R/K147Y system in which the glycan was initially placed with a pronounced off-axis deviation. All of these supplementary umbrella sampling simulations were performed using the same SMD protocol described above. The potential of mean force was calculated using the weighted histogram analysis method with sampling windows extending from the *cis* vestibule to the *trans* side of the pore in the M113R/K147Y system [62], thereby enabling a more complete description of glycan transport through the nanopore.

Furthermore, to examine whether our conclusions depend on the choice of ensemble and umbrella sampling simulation, new constant-velocity SMD simulations were additionally carried out under the NVT ensemble for the 2 key systems, M113R and M113R/K147Y [33,63]. These production simulations were performed for 2,000 ps with a 2-fs timestep after energy minimization and a 6-step equilibration procedure. The temperature was maintained at 298.15 K using a velocity-rescaling thermostat and the same pulling potential.

The calculation was based on the gmx commands in GROMACS. Interaction analysis was conducted using ProLIF [64], while CH–π interaction was recognized by CH–π distance < 0.45 nm and angle < 40° [65].

## Data Availability

All data supporting the findings of this study are included within the article and its Supplementary Materials, as well as from the corresponding authors upon reasonable request.

## References

[1] Kalinichenko LS, Gulbins E, Kornhuber J, Müller CP. Sphingolipid control of cognitive functions in health and disease. Prog Lipid Res. 2022;86: Article 101162. 35318099 10.1016/j.plipres.2022.101162

[2] Schnaar RL. Gangliosides of the vertebrate nervous system. J Mol Biol. 2016;428(16):3325–3336. 27261254 10.1016/j.jmb.2016.05.020 PMC4983208

[3] Tseng H-K, Wu C-Y, Tseng H-W, Lyu K-H, Kuo W-H, Angata T, Lin C-C. Characterization of human sialyltransferase ST6GalNAc5 and its use in the synthesis of internally α2,6-sialylated glycans. ACS Catal. 2025;15(21):17815–17828.

[4] Yan M, Su A, Meyer D, Roman Sosa G, Fritsch H, Pitters M, Fischer N, Herrler G, Becher P. Precursor of H-type II histo-blood group antigen and subterminal sialic acids on gangliosides are significantly implicated in cell entry and infection by a porcine P[11] rotavirus. Emerg Microbes Infect. 2025;14(1):2447608. 39726161 10.1080/22221751.2024.2447608 PMC11727068

[5] Schnaar RL, Gerardy-Schahn R, Hildebrandt H. Sialic acids in the brain: Gangliosides and polysialic acid in nervous system development, stability, disease, and regeneration. Physiol Rev. 2014;94(2):461–518. 24692354 10.1152/physrev.00033.2013 PMC4044301

[6] Adak AK, Tseng HK, Chang SY, Chiang YC, Lyu KH, Lee YS, Lu W, Kuo WH, Angata T, Lin CC. Comprehensive modular synthesis of ganglioside glycans and evaluation of their binding affinities to Siglec-7 and Siglec-9. Adv Sci. 2025;12(2): Article e2412815. 10.1002/advs.202412815 PMC11727393 39555730

[7] Monyror J, Kadam V, Morales LC, Ordóñez D, Ibanga J, Zaidi AK, McNamara E, Pink D, Stenlund M, Reyes K, et al. Gangliosides modulate the secretion of extracellular vesicles and their misfolded protein cargo. Sci Adv. 2025;11(38):eady5212. 40961208 10.1126/sciadv.ady5212 PMC12442881

[8] Frosch M, Shimizu T, Wogram E, Amann L, Gruber L, Groisman AI, Fliegauf M, Schwabenland M, Chhatbar C, Zechel S, et al. Microglia–neuron crosstalk through Hex–GM2–MGL2 maintains brain homeostasis. Nature. 2025;646(8086):913–924. 40769205 10.1038/s41586-025-09477-y PMC12545202

[9] Hülsmeier AJ. Glycosphingolipids in neurodegeneration—Molecular mechanisms, cellular roles, and therapeutic perspectives. Neurobiol Dis. 2025;207: Article 106851. 39978484 10.1016/j.nbd.2025.106851

[10] Lee J, Yin D, Yun J, Kim M, Kim SW, Hwang H, Park JE, Lee B, Lee CJ, Shin HS, et al. Deciphering mouse brain spatial diversity via glyco-lipidomic mapping. Nat Commun. 2024;15(1):8689. 39375371 10.1038/s41467-024-53032-8 PMC11458762

[11] Ica R, Sarbu M, Biricioiu R, Fabris D, Vukelić Ž, Zamfir AD. Novel application of ion mobility mass spectrometry reveals complex ganglioside landscape in diffuse astrocytoma peritumoral regions. Int J Mol Sci. 2025;26(17):8433. 40943353 10.3390/ijms26178433 PMC12428295

[12] Biricioiu MR, Mlinac-Jerković K, Ilic K, Sajko T, Ica R, Sarbu M, Clemmer DE, Kalanj-Bognar S, Zamfir AD. Advanced ganglioside characterization in epileptic human hippocampus by travelling waves ion mobility tandem mass spectrometry. J Mass Spectrom. 2025;60(11): Article e5190. 41065107 10.1002/jms.5190

[13] Muggli T, Bühr C, Schürch S. Challenges in the analysis of gangliosides by LC-MS. Chimia. 2022;76(1–2):109–113. 38069756 10.2533/chimia.2022.109

[14] Nie H, Li Y, Sun XL. Recent advances in sialic acid-focused glycomics. J Proteome. 2012;75(11):3098–3112. 10.1016/j.jprot.2012.03.050 PMC3367064 22513219

[15] Williamson DL, Naylor CN, Nagy G. Sequencing sialic acid positioning in gangliosides by high-resolution cyclic ion mobility separations coupled with multiple collision-induced dissociation-based tandem mass spectrometry strategies. Anal Chem. 2024;96(34):14068–14073. 10.1021/acs.analchem.4c03411 PMC11822045 39137259

[16] Ying Y-L, Hu Z-L, Zhang S, Qing Y, Fragasso A, Maglia G, Meller A, Bayley H, Dekker C, Long Y-T. Nanopore-based technologies beyond DNA sequencing. Nat Nanotechnol. 2022;17(11):1136–1146. 36163504 10.1038/s41565-022-01193-2

[17] Lu C, Bonini A, Viel JH, Maglia G. Toward single-molecule protein sequencing using nanopores. Nat Biotechnol. 2025;43(3):312–322. 40097683 10.1038/s41587-025-02587-y PMC12006967

[18] Yao G, Ke W, Xia B, Gao Z. Nanopore-based glycan sequencing: State of the art and future prospects. Chem Sci. 2024;15(17):6229–6243. 38699252 10.1039/d4sc01466a PMC11062086

[19] Bhatti H, Ying Y-L, Long Y-T. How to compare the ion selectivity of smart nanopores/membranes. Research. 2024;7: Article 0506. 40771577 10.34133/research.0506 PMC12327432

[20] Jiang J, Li MY, Wu XY, Ying YL, Han HX, Long YT. Protein nanopore reveals the renin–angiotensin system crosstalk with single-amino-acid resolution. Nat Chem. 2023;15(4):578–586. 36805037 10.1038/s41557-023-01139-8

[21] Bayat P, Rambaud C, Priem B, Bourderioux M, Bilong M, Poyer S, Pastoriza-Gallego M, Oukhaled A, Mathé J, Daniel R. Comprehensive structural assignment of glycosaminoglycan oligo- and polysaccharides by protein nanopore. Nat Commun. 2022;13(1):5113. 36042212 10.1038/s41467-022-32800-4 PMC9427770

[22] Ratinho L, Meyer N, Greive S, Cressiot B, Pelta J. Nanopore sensing of protein and peptide conformation for point-of-care applications. Nat Commun. 2025;16(1):3211. 40180898 10.1038/s41467-025-58509-8 PMC11968944

[23] Xia B, Fang J, Ma S, Ma M, Yao G, Li T, Cheng X, Wen L, Gao Z. Mapping the acetylamino and carboxyl groups on glycans by engineered α-hemolysin nanopores. J Am Chem Soc. 2023;145(34):18812–18824. 37527445 10.1021/jacs.3c03563

[24] Zhang S, Cao Z, Fan P, Wang Y, Jia W, Wang L, Wang K, Liu Y, Du X, Hu C, et al. A nanopore-based saccharide sensor. Angew Chem Int Ed Engl. 2022;61(33): Article e202203769. 35718742 10.1002/anie.202203769

[25] Gao F, Wang JH, Ma H, Xia B, Wen L, Long YT, Ying YL. Identification of oligosaccharide isomers using electrostatically asymmetric OmpF nanopore. Angew Chem Int Ed Engl. 2025;64(9): Article e202422118. 39856493 10.1002/anie.202422118

[26] Li M, Xiong Y, Cao Y, Zhang C, Li Y, Ning H, Liu F, Zhou H, Li X, Ye X, et al. Identification of tagged glycans with a protein nanopore. Nat Commun. 2023;14(1):1737. 36977665 10.1038/s41467-023-37348-5 PMC10050315

[27] Yao G, Tian Y, Ke W, Fang J, Ma S, Li T, Cheng X, Xia B, Wen L, Gao Z. Direct identification of complex glycans via a highly sensitive engineered nanopore. J Am Chem Soc. 2024;146(19):13356–13366. 38602480 10.1021/jacs.4c02081

[28] Versloot RCA, Lucas FLR, Yakovlieva L, Tadema MJ, Zhang Y, Wood TM, Martin NI, Marrink SJ, Walvoort MTC, Maglia G. Quantification of protein glycosylation using nanopores. Nano Lett. 2022;22(13):5357–5364. 35766994 10.1021/acs.nanolett.2c01338 PMC9284675

[29] Wang JH, Ma W, Hu ZL, Gao Z, Long YT, Li T, Ying YL. Direct identification of O-glycopeptides by low-temperature assisted nanopore technique. Research. 2025;8:0850. 40917498 10.34133/research.0850 PMC12410930

[30] Yao G, Xia B, Wei F, Wang J, Yang Y, Ma S, Ke W, Li T, Cheng X, Wen L, et al. Glycan sequencing based on glycosidase-assisted nanopore sensing. J Am Chem Soc. 2025;147(2):1721–1731. 39745005 10.1021/jacs.4c12940

[31] Huang G, Willems K, Soskine M, Wloka C, Maglia G. Electro-osmotic capture and ionic discrimination of peptide and protein biomarkers with FraC nanopores. Nat Commun. 2017;8(1):935. 29038539 10.1038/s41467-017-01006-4 PMC5715100

[32] Gu LQ, Cheley S, Bayley H. Electroosmotic enhancement of the binding of a neutral molecule to a transmembrane pore. Proc Natl Acad Sci USA. 2003;100(26):15498–15503. 14676320 10.1073/pnas.2531778100 PMC307596

[33] Bonome EL, Cecconi F, Chinappi M. Electroosmotic flow through an α-hemolysin nanopore. Microfluid Nanofluid. 2017;21(5):96.

[34] Zhang S, Cao Z, Fan P, Sun W, Xiao Y, Zhang P, Wang Y, Huang S. Discrimination of disaccharide isomers of different glycosidic linkages using a modified MspA nanopore. Angew Chem Int Ed Engl. 2024;63(8): Article e202316766. 38116834 10.1002/anie.202316766

[35] Lu W, Zhao X, Li M, Li Y, Zhang C, Xiong Y, Li J, Zhou H, Ye X, Li X, et al. Precise structural analysis of neutral glycans using aerolysin mutant T240R nanopore. ACS Nano. 2024;18(19):12412–12426. 38693619 10.1021/acsnano.4c01571

[36] Kiessling LL, Diehl RC. CH−π interactions in glycan recognition. ACS Chem Biol. 2021;16(10):1884–1893. 34615357 10.1021/acschembio.1c00413 PMC9004545

[37] Asandei A, Apetrei A, Park Y, Hahm KS, Luchian T. Investigation of single-molecule kinetics mediated by weak hydrogen bonds within a biological nanopore. Langmuir. 2011;27(1):19–24. 21128603 10.1021/la104264f

[38] Stoddart D, Heron AJ, Klingelhoefer J, Mikhailova E, Maglia G, Bayley H. Nucleobase recognition in ssDNA at the central constriction of the α-hemolysin pore. Nano Lett. 2010;10(9):3633–3637. 20704324 10.1021/nl101955a PMC2935931

[39] Manara RM, Tomasio S, Khalid S. The nucleotide capture region of alpha hemolysin: Insights into nanopore design for DNA sequencing from molecular dynamics simulations. Nanomaterials. 2015;5(1):144–153. 28347003 10.3390/nano5010144 PMC5312860

[40] De Biase PM, Ervin EN, Pal P, Samoylova O, Markosyan S, Keehan MG, Barrall GA, Noskov SY. What controls open-pore and residual currents in the first sensing zone of alpha-hemolysin nanopore? Combined experimental and theoretical study. Nanoscale. 2016;8(22):11571–11579. 27210516 10.1039/c6nr00164e

[41] Tettamanti G, Bonali F, Marchesini S, Zambotti V. A new procedure for the extraction, purification and fractionation of brain gangliosides. Biochim Biophys Acta. 1973;296(1):160–170. 4693502 10.1016/0005-2760(73)90055-6

[42] Galleguillos D, Wang Q, Steinberg N, Zaidi A, Shrivastava G, Dhami K, Daskhan GC, Schmidt EN, Dworsky-Fried Z, Giuliani F, et al. Anti-inflammatory role of GM1 and other gangliosides on microglia. J Neuroinflammation. 2022;19(1):9. 34991625 10.1186/s12974-021-02374-x PMC8739653

[43] Hynds DL, Summers M, Van Brocklyn J, O’Dorisio MS, Yates AJ. Gangliosides inhibit platelet-derived growth factor-stimulated growth, receptor phosphorylation, and dimerization in neuroblastoma SH-SY5Y cells. J Neurochem. 1995;65(5):2251–2258. 7595514 10.1046/j.1471-4159.1995.65052251.x

[44] Moremen KW, Ramiah A, Stuart M, Steel J, Meng L, Forouhar F, Moniz HA, Gahlay G, Gao Z, Chapla D, et al. Expression system for structural and functional studies of human glycosylation enzymes. Nat Chem Biol. 2018;14(2):156–162. 29251719 10.1038/nchembio.2539 PMC5774587

[45] Xu Z, Liu Y, Liu J, Ma W, Zhang Z, Chapla DG, Wen L, Moremen KW, Yi W, Li T. Integrated chemoenzymatic synthesis of a comprehensive sulfated ganglioside glycan library to decipher functional sulfoglycomics and sialoglycomics. Nat Chem. 2024;16(6):881–892. 38844638 10.1038/s41557-024-01540-x PMC12382592

[46] Waterhouse A, Bertoni M, Bienert S, Studer G, Tauriello G, Gumienny R, Heer FT, de Beer TAP, Rempfer C, Bordoli L, et al. SWISS-MODEL: Homology modelling of protein structures and complexes. Nucleic Acids Res. 2018;46(W1):W296–W303. 29788355 10.1093/nar/gky427 PMC6030848

[47] Cai Z, Peng H, Sun S, He J, Luo F, Huang Y. DeepKa web server: High-throughput protein p*K*_a_ prediction. J Chem Inf Model. 2024;64(8):2933–2940. 38530291 10.1021/acs.jcim.3c02013

[48] Shen M, Kortzak D, Ambrozak S, Bhatnagar S, Buchanan I, Liu R, Shen J. KaMLs for predicting protein p*K*_a_ values and ionization states: Are trees all you need? J Chem Theory Comput. 2025;21(3):1446–1458. 39882632 10.1021/acs.jctc.4c01602 PMC12323819

[49] Anandakrishnan R, Aguilar B, Onufriev AV. *H*++ 3.0: Automating p*K* prediction and the preparation of biomolecular structures for atomistic molecular modeling and simulations. Nucleic Acids Res. 2012;40(W1):W537–W541. 22570416 10.1093/nar/gks375 PMC3394296

[50] Olsson MH, Søndergaard CR, Rostkowski M, Jensen JH. PROPKA3: Consistent treatment of internal and surface residues in empirical p*K*_a_ predictions. J Chem Theory Comput. 2011;7(2):525–537. 26596171 10.1021/ct100578z

[51] Bochkov AY, Toukach PV. CSDB/SNFG Structure Editor: An online glycan builder with 2D and 3D structure visualization. J Chem Inf Model. 2021;61(10):4940–4948. 34595926 10.1021/acs.jcim.1c00917

[52] Wu EL, Cheng X, Jo S, Rui H, Song KC, Dávila-Contreras EM, Qi Y, Lee J, Monje-Galvan V, Venable RM, et al. CHARMM-GUI *Membrane Builder* toward realistic biological membrane simulations. J Comput Chem. 2014;35(27):1997–2004. 25130509 10.1002/jcc.23702 PMC4165794

[53] Hamel M, Henault M, Hyjazie H, Morin N, Bayly C, Skorey K, Therien AG, Mancini J, Brideau C, Kargman S. Discovery of novel P2Y14 agonist and antagonist using conventional and nonconventional methods. J Biomol Screen. 2011;16(9):1098–1105. 21821827 10.1177/1087057111415525

[54] Huang J, Rauscher S, Nawrocki G, Ran T, Feig M, de Groot BL, Grubmüller H, MacKerell AD Jr. CHARMM36m: An improved force field for folded and intrinsically disordered proteins. Nat Methods. 2017;14(1):71–73. 27819658 10.1038/nmeth.4067 PMC5199616

[55] Guvench O, Mallajosyula SS, Raman EP, Hatcher E, Vanommeslaeghe K, Foster TJ, Jamison FW II, Mackerell AD Jr. CHARMM additive all-atom force field for carbohydrate derivatives and its utility in polysaccharide and carbohydrate–protein modeling. J Chem Theory Comput. 2011;7(10):3162–3180. 22125473 10.1021/ct200328p PMC3224046

[56] Berendsen HJC, van der Spoel D, van Drunen R. GROMACS: A message-passing parallel molecular dynamics implementation. Comput Phys Commun. 1995;91(1):43–56.

[57] Liang J, Inoue A, Ikuta T, Xia R, Wang N, Kawakami K, Xu Z, Qian Y, Zhu X, Zhang A, et al. Structural basis of lysophosphatidylserine receptor GPR174 ligand recognition and activation. Nat Commun. 2023;14(1):1012. 36823105 10.1038/s41467-023-36575-0 PMC9950150

[58] Bussi G, Donadio D, Parrinello M. Canonical sampling through velocity rescaling. J Chem Phys. 2007;126(1): Article 014101. 17212484 10.1063/1.2408420

[59] Bernetti M, Bussi G. Pressure control using stochastic cell rescaling. J Chem Phys. 2020;153(11): Article 114107. 32962386 10.1063/5.0020514

[60] Lemkul JA, Bevan DR. Assessing the stability of Alzheimer’s amyloid protofibrils using molecular dynamics. J Phys Chem B. 2010;114(4):1652–1660. 20055378 10.1021/jp9110794

[61] Black KA, He S, Jin R, Miller DM, Bolla JR, Clarke OB, Johnson P, Windley M, Burns CJ, Hill AP, et al. A constricted opening in Kir channels does not impede potassium conduction. Nat Commun. 2020;11(1):3024. 32541684 10.1038/s41467-020-16842-0 PMC7295778

[62] Hub JS, de Groot BL, van der Spoel D. g_wham—A free weighted histogram analysis implementation including robust error and autocorrelation estimates. J Chem Theory Comput. 2010;6(12):3713–3720.

[63] Aksimentiev A, Schulten K. Imaging *α*-hemolysin with molecular dynamics: Ionic conductance, osmotic permeability, and the electrostatic potential map. Biophys J. 2005;88(6):3745–3761. 15764651 10.1529/biophysj.104.058727 PMC1305609

[64] Bouysset C, Fiorucci S. ProLIF: A library to encode molecular interactions as fingerprints. J Cheminform. 2021;13(1):72. 34563256 10.1186/s13321-021-00548-6 PMC8466659

[65] Hudson KL, Bartlett GJ, Diehl RC, Agirre J, Gallagher T, Kiessling LL, Woolfson DN. Carbohydrate–aromatic interactions in proteins. J Am Chem Soc. 2015;137(48):15152–15160. 26561965 10.1021/jacs.5b08424 PMC4676033

