# Supplementary material for: Single-Molecule Discrimination of Multisialylated Ganglioside Oligosaccharides Using an Engineered Nanopore
Source: Research (Wash D C). 2026 May 19;9:1286. doi: 10.34133/research.1286 (PMC13184820; doi:10.34133/research.1286)

Supporting Information

Nanopore Sensor Designed for Multisialylated Ganglioside Oligosaccharides

Guangda Yao[1†], Boyang Ren[2,1†], Daigui Zhu[3,4†], Jianling Tan[1,5†], Jingjing Hou[1,6], Yuan Ma[4], Zhengyu Hang[1], Zhuojia Xu[4], Zhaobing Gao[1,5*], Tiehai Li[3,4*], Bingqing Xia[1,5*]

[1]State Key Laboratory of Drug Research, Shanghai Institute of Materia Medica, Chinese Academy of Sciences, Shanghai, 201203, China.
[2]Tianjin University of Traditional Chinese Medicine, Tianjin 301617, China.
[3]School of Chinese Materia Medica, Nanjing University of Chinese Medicine, Nanjing 210023, China.
[4]State Key Laboratory of Chemical Biology, Shanghai Institute of Materia Medica, Chinese Academy of Sciences, Shanghai 201203, China.
[5]University of Chinese Academy of Sciences, Beijing, 100049, China.
[6]School of Pharmacy, Fudan University, Shanghai 201203, China

*Address correspondence to: Bingqing Xia; xiabingqing@simm.ac.cn and Tiehai Li; tiehaili@simm.ac.cn and Zhaobing Gao; zbgao@simm.ac.cn

†These authors contributed equally to this work.

Table of Contents

1. Supporting figures 3

Figure S1. Purification and characterization of wild-type (WT) α-hemolysin (α-HL) nanopore and their mutants. 3

Figure S2. Representative current traces and scatter plots of GT1c sensed by α-HL (WT) and its mutants individually. 5

Figure S3. Ion selectivity of the α-hemolysin (WT) and M113R mutant. 6

Figure S4. Modular simulation and analysis of GT1c in α-HL (M113R) nanopore systems. 7

Figure S5. Representative traces and scatter plot of GT1c detection by α-HL (M113R) in low-concentration electrolyte buffer. 9

Figure S6. Representative traces and scatter plot of GT1c detection by α-HL (M113R/K147Y) in low-concentration electrolyte buffer. 10

Figure S7. Schematic diagram of current blockage event extraction and feature definition. 1

Figure S8. Detection of GT1c under gradient voltages using α-HL (M113R/K147Y). 2

Figure S9. Detection of GT1c at gradient concentrations using α-HL (M113R/K147Y). 4

Figure S10. Modular simulation and analysis of GT1c in M113R/K147Y systems. 5

Figure S11. The temporal variation of interactions in M113R/K147Y system over the course of GT1c translocating under NPT using umbrella sampling or NVT ensemble using constant-velocity SMD. 6

Figure S12. Analysis of different initial glycan position in M113R and M113R/K147Y systems 7

Figure S13. Analysis of GT1c conformation in M113R and M113R/K147Y systems. 9

Figure S14. Detection of structural isomers using α-HL (M113R/K147Y). 10

Figure S15. Statistical analysis and class-wise feature patterns of standardized nanopore event descriptors. 11

Figure S16. Validation-set performance comparison of candidate classifiers for glycan identification from nanopore event features. 13

Figure S17. Single-event resolved scatter plot of structural isomers via UMAP Clustering. 14

Figure S18. Detection of GQ1c and GP1c using α-HL (M113R/K147Y). 15

Figure S19. Learning Curve of the MLP Classifier (10-Fold Cross-Validation). 16

Figure S20. Detection of glycan mixture using α-HL (M113R/K147Y). 17

Figure S21. Single-event resolved scatter plot of six glycan mixtures via UMAP Clustering. 18

Figure S22. Detection of lysate-free referencee and lysate using α-HL (M113R/K147Y). 19

Figure S23. Detection of glycan mixture and lysate using α-HL (M113R/K147Y). 20

Figure S24. Single-event resolved scatter plot of six glycan mixtures in cell lysate via UMAP Clustering. 21

Figure S25. Single-event resolved scatter plot of six glycan mixtures in brain tissue lysate via UMAP Clustering. 22

2. Supporting tables 23

Table S1. Detection Frequency, Fitted Dwell Time, and Fitted $\Delta I_1/I_0$ of GT1c by WT and Individual Mutants. 23

Table S2. Definitions of six current signal features. 24

Table S3. Fitted dwell time values for GT1c detection under voltage gradients. 25

Table S4. Gaussian fitted $\Delta I_1/I_0$ values for GT1c across concentration gradients. 26

Table S5. Interactions of GT1c and residues of α-HL in M113R and M113R/K147Y systems of MD simulations. 27

Table S6. The pKa values calculated for titratable residues of sensing region in α-HL (M113R) and α-HL (M113R/K147Y). 28

3. Definition and formulas for the six features. 30

4. Synthesis and characterization of Ganglioside Oligosaccharides 32

(1) General materials 32

(2) Enzymatic Synthesis methods of ganglioside glycans 32

(3) NMR Spectra 36

1. Supporting figures

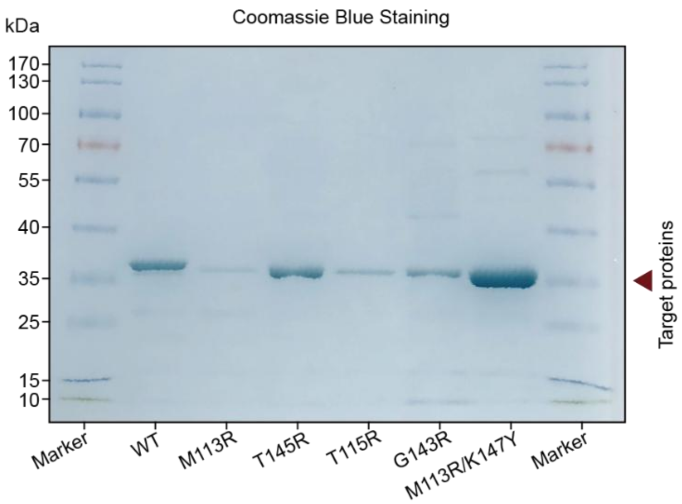

Figure S1. Purification and characterization of wild-type (WT) α-hemolysin (α-HL) nanopore and their mutants. Coomassie brilliant blue staining of wild-type α-hemolysin (WT) and its mutants obtained through expression using *E coli* and purification by Ni-NTA affinity column. The lanes from left to right are: Marker, α-HL (WT), α-HL (M113R), α-HL (T145R), α-HL (T115R), α-HL (G143R), α-HL (M113R/K147Y), and Marker. The molecular weight of the α-HL (WT) monomer is 35.5 kDa, and the target protein is indicated by a red triangle**.**

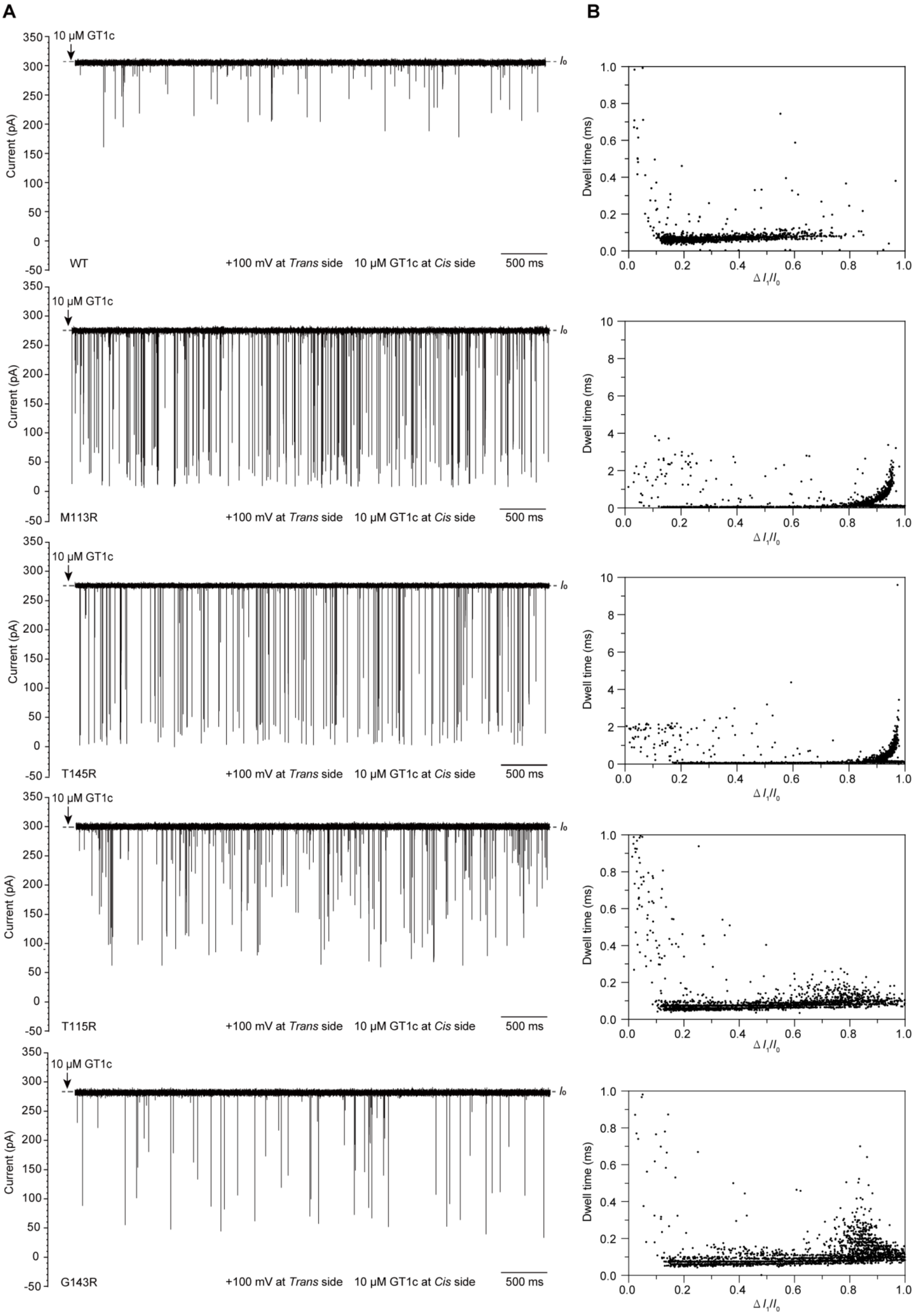
A
B
10 μM GT1c
Current (pA)
I₀
WT
M113R
T145R
T115R
G143R
+100 mV at Trans side
10 μM GT1c at Cis side
500 ms
Dwell time (ms)
Δ I₁/I₀

Figure S2. Representative current traces and scatter plots of GT1c sensed by α-HL (WT) and its mutants individually. (A) Representative ionic current traces of GT1c sensed by α-HL (WT), α-HL (M113R), α-HL (T145R), α-HL (T115R), and α-HL (G143R) nanopores. (B) Scatter plots of Dwell time versus $\Delta I_1/I_0$ derived from the current blockage events extracted from the traces in (A); each scatter plot contains at least 3000 events. All nanopore experiments were performed in symmetric electrolyte solution (3 M KCl, 10 mM citric acid, pH=5.0), with the *Cis* side grounded and a potential of +100 mV applied to the *Trans* side. Each glycan was added to the *Cis* side to a final concentration of 10 μM. n ≥ 3.

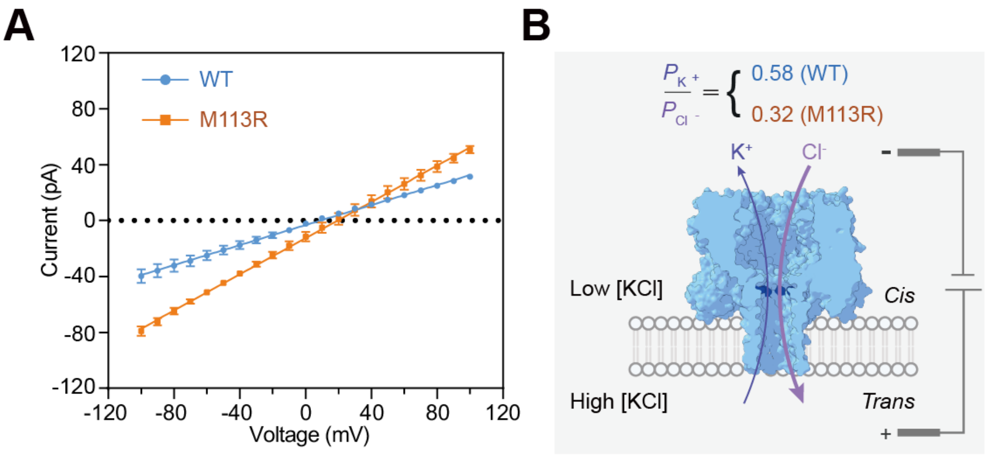

Figure S3. Ion selectivity of the α-hemolysin (WT) and M113R mutant.

(A) Reversal potential measurements, which indicated that both α-hemolysin (WT) and the M113R mutant are anion-selective. Reversal potentials were determined under asymmetric electrolyte conditions (1 M KCl in the *Trans* reservoir and 0.1 M KCl in the *Cis* reservoir), with both solutions buffered in 10 mM citric acid at pH 5.0. (B) Ion selectivity calculation and schematic illustration. The ion selectivity was calculated using the Goldman–Hodgkin–Katz equation. A constant voltage was applied to the *Trans* side.

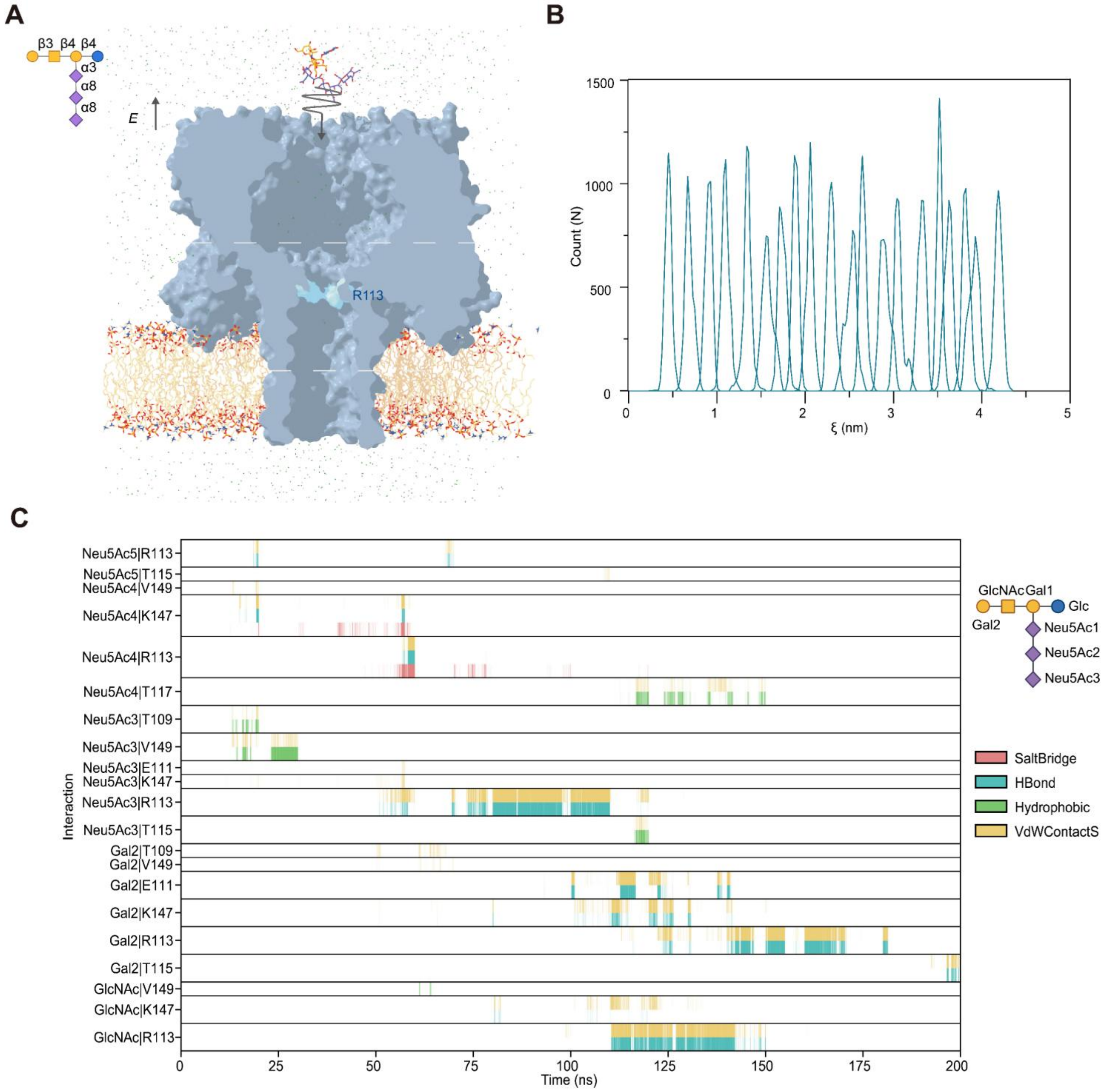

Figure S4. Modular simulation and analysis of GT1c in α-HL (M113R) nanopore systems.
(A) All-atom model where an α-HL pore (Marked in blue) confines GT1c. Glc, GalNAc, Gal, and Neu5Ac are shown as blue, orange, orange, and purple sticks, respectively. Potassium ions, chloride ions and phospholipids bilayer are shown as purple spheres, green spheres and tan sticks, respectively. Glycan displaced along the pore axis by means of a harmonic force. Umbrella sampling analysis was conducted over the R113 (blue surface), indicated by the two white dashed lines. *E* indicates the applied constant electric field. (B) 20 histograms, a total of 200-ns simulation, were extracted from umbrella simulations over the course of GT1c translocating through R113 in systems of M113R. ξ was defined as the distance between COMs of two pulling groups, Neu5Ac and the lipid bilayer. (C) The temporal variation of interactions that occurred with a frequency exceeding 0.5% are displayed. The types of interactions are denoted at the right of the figure.

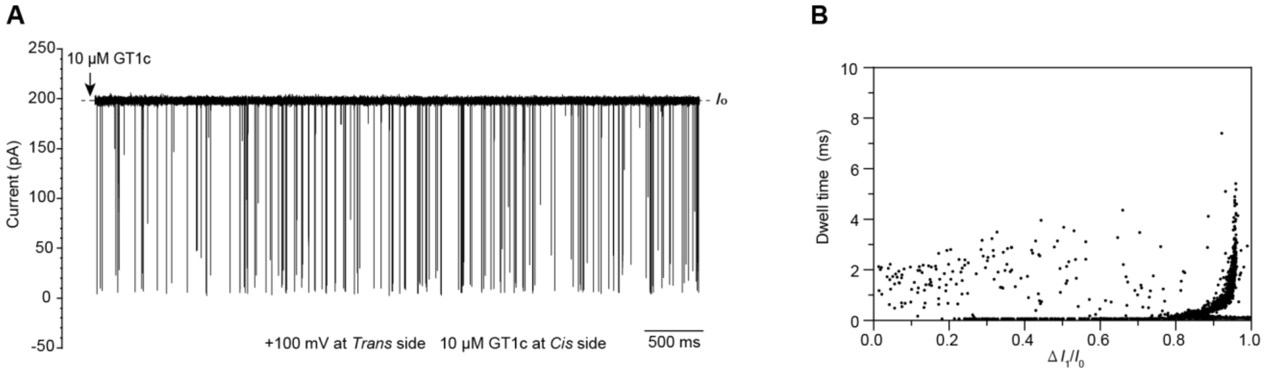

Figure S5. Representative traces and scatter plot of GT1c detection by α-HL (M113R) in low-concentration electrolyte buffer.

(A) Representative ionic current traces of GT1c sensed by α-HL (M113R) nanopores in a symmetric electrolyte solution of 2 M KCl (pH=5.0). (B) Scatter plots of Dwell time versus $\Delta I_1/I_0$ derived from the current blockage events extracted from the traces in (A). All nanopore experiments were performed in symmetric electrolyte solution (2 M KCl, 10 mM citric acid, pH=5.0), with the *Cis* side grounded and a potential of +100 mV applied to the *Trans* side. Each glycan was added to the *Cis* side to a final concentration of 10 μM. n ≥ 3, and each scatter plot contains at least 3000 events.

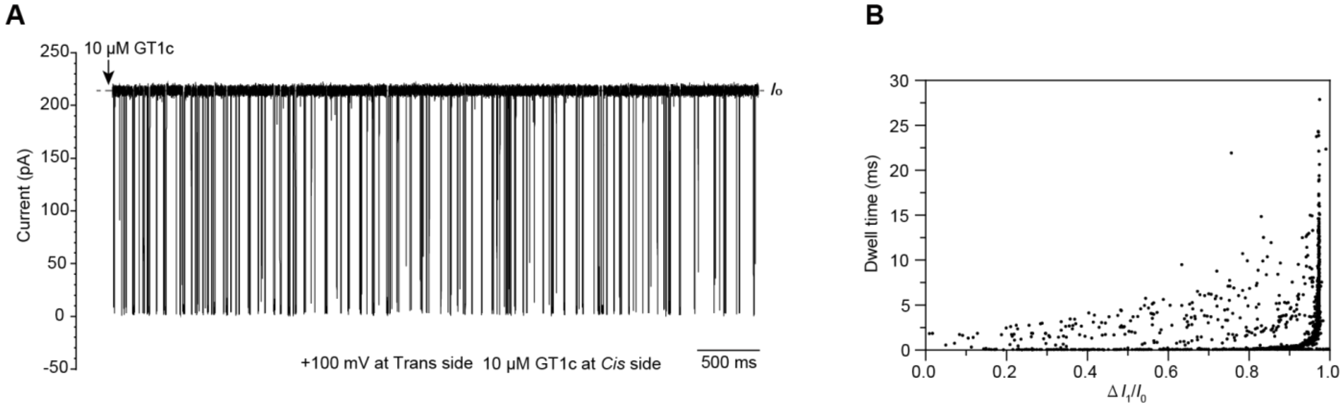

Figure S6. Representative traces and scatter plot of GT1c detection by α-HL (M113R/K147Y) in low-concentration electrolyte buffer.

(A) Representative ionic current traces of GT1c sensed by α-HL (M113R/K147Y) nanopores in a symmetric electrolyte solution of 2 M KCl (pH=5.0). (B) Scatter plots of Dwell time versus $\Delta I_1/I_0$ derived from the current blockage events extracted from the traces in (A). All nanopore experiments were performed in a symmetric electrolyte solution (2 M KCl, 10 mM citric acid, pH=5.0), with the *Cis* side grounded and a potential of +100 mV applied to the *Trans* side. Each glycan was added to the *Cis* side to a final concentration of 10 μM. n ≥ 3, and each scatter plot contains at least 3000 events.

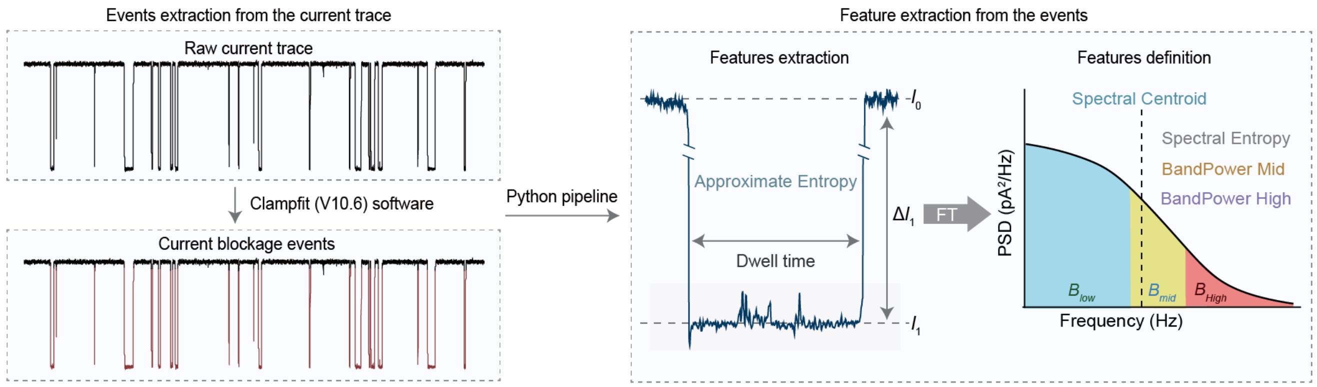

Figure S7. Schematic diagram of current blockage event extraction and feature definition.

The data analysis workflow consists of two main stages: (Left) identification and extraction of individual current blockage events from raw traces using Clampfit (V10.6), followed by (Right) automated calculation of event parameters via a customized Python pipeline, with Fourier Transform (FT) employed for frequency-domain analysis. Abbreviation: open-pore current (I_0), average blockage current (I_1), I_1-I_0 (ΔI_1).

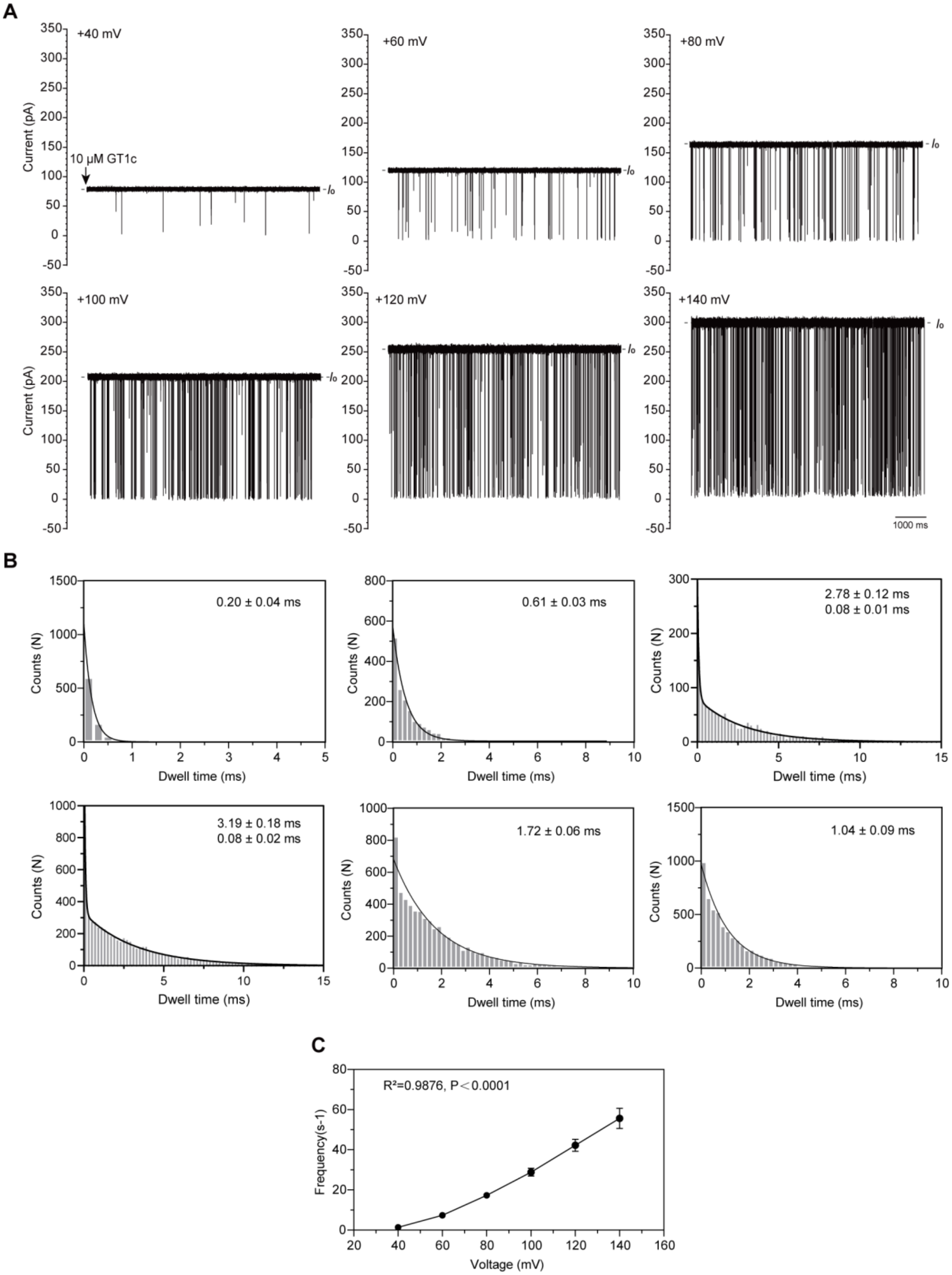

Figure S8. Detection of GT1c under gradient voltages using α-HL (M113R/K147Y).

(A) Representative ionic current traces of the GT1c oligosaccharide captured by α-HL (M113R/K147Y) under different voltages. The voltage range spans from +40 mV to +160 mV with 20 mV increments. (B) Frequency distribution histograms of Dwell time with exponential fitting for events in (A). (C) Line plot of event frequency versus the voltage applied to the Trans side. All nanopore experiments were performed in a symmetric electrolyte solution (2 M KCl, 10

mM citric acid, pH=5.0), with the *Cis* side grounded and a series of gradient voltages applied to the *Trans* side. Each glycan sample was added to the *Cis* side to a final concentration of 10 μM. For each condition, n ≥ 3

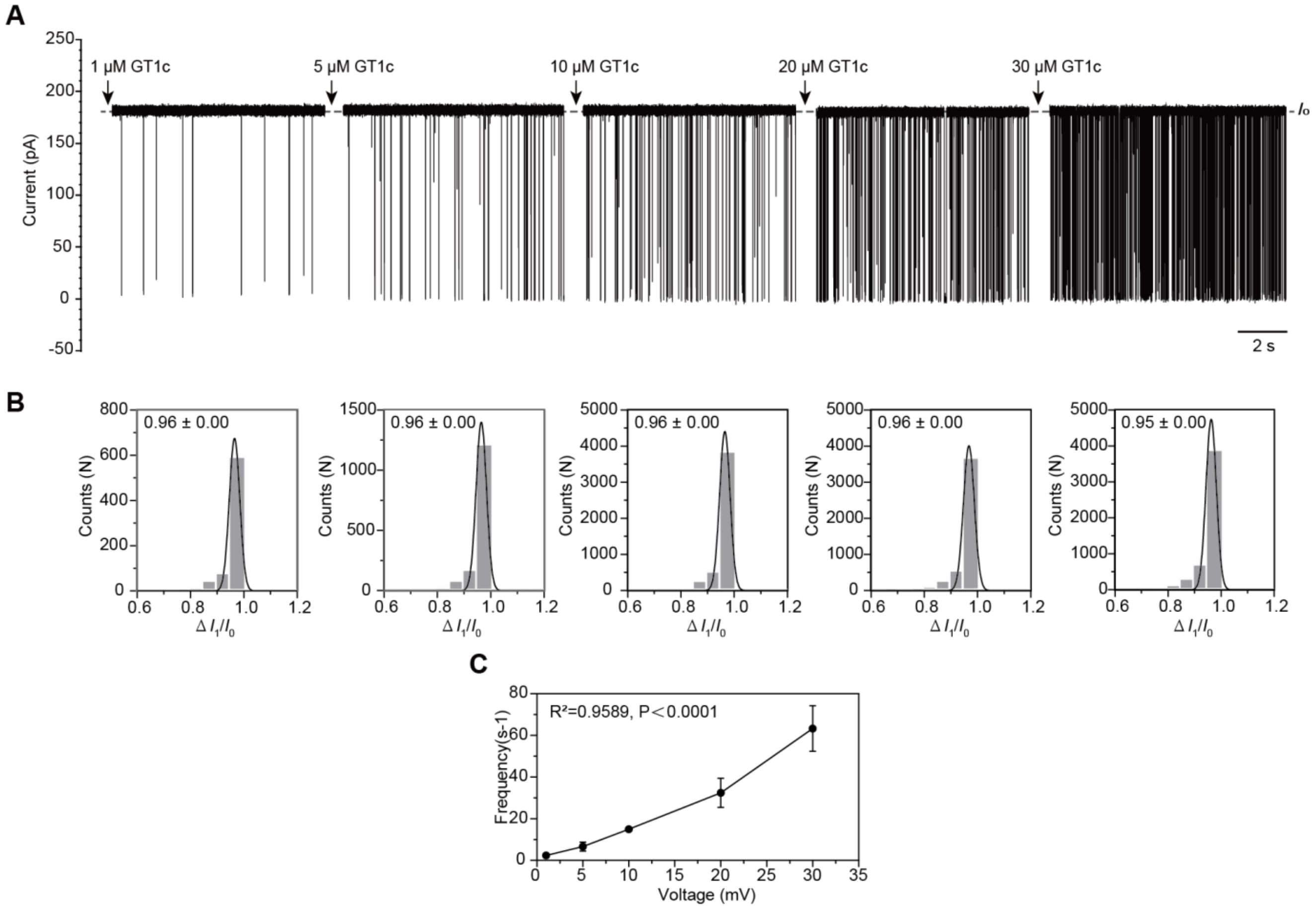

Figure S9. Detection of GT1c at gradient concentrations using α-HL (M113R/K147Y).

(A) Representative ionic current traces of the oligosaccharide moiety of GT1c oligosaccharide detected by α-HL (M113R/K147Y) under different glycan concentrations. Concentration gradients correspond to 1 μM, 5 μM, 10 μM, 20 μM, and 30 μM. (B) Frequency distribution histograms with exponential fitting of $\Delta I_1/I_0$ for events in (A) The histograms show approximately 5–10 visible bins, exhibiting a Gaussian-like distribution trend. (C) Line plot of event frequency versus glycan concentration. All nanopore experiments were performed in a symmetric electrolyte solution (2 M KCl, 10 mM citric acid, pH=5.0), with the *Cis* side grounded and a potential of +100 mV applied to the *Trans* side. Each glycan was added to the *Cis* side. n ≥ 3.

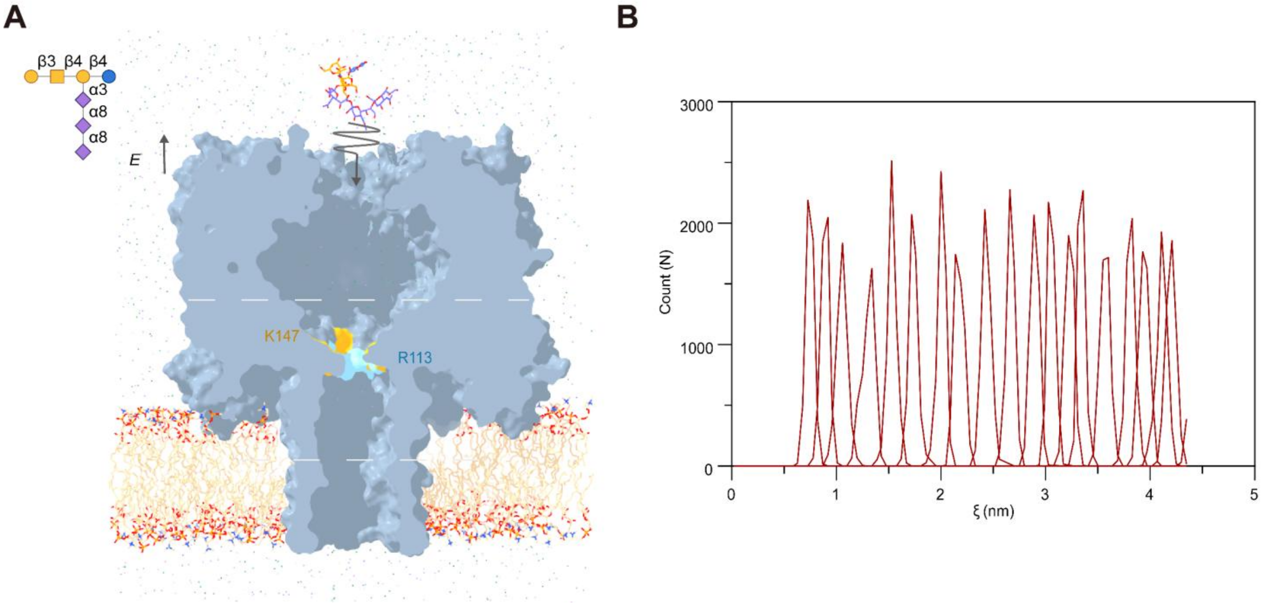

Figure S10. Modular simulation and analysis of GT1c in M113R/K147Y systems.

(A) All-atom model where an α-HL pore (blue) confines GT1c. Glc, GalNAc, Gal, and Neu5Ac are shown as blue, orange, orange, and purple sticks, respectively. Potassium ions, chloride ions and phospholipids bilayer are shown as purple spheres, green spheres and tan sticks, respectively. Glycan displaced along the pore axis by means of a harmonic force. Umbrella sampling analysis was conducted over the R113 (blue surface) and Y147 (goldenrod surface), indicated by the two white dashed lines. *E* indicate the applied constant electric field. (B) 20 histograms, a total of 200-ns simulation, were extracted from umbrella simulations over the course of GT1c translocating in systems of M113R/K147Y. ξ was defined as the distance between COMs of two pulling groups, N-acetylneuraminic acid (Neu5Ac) and the planar lipid bilayer.

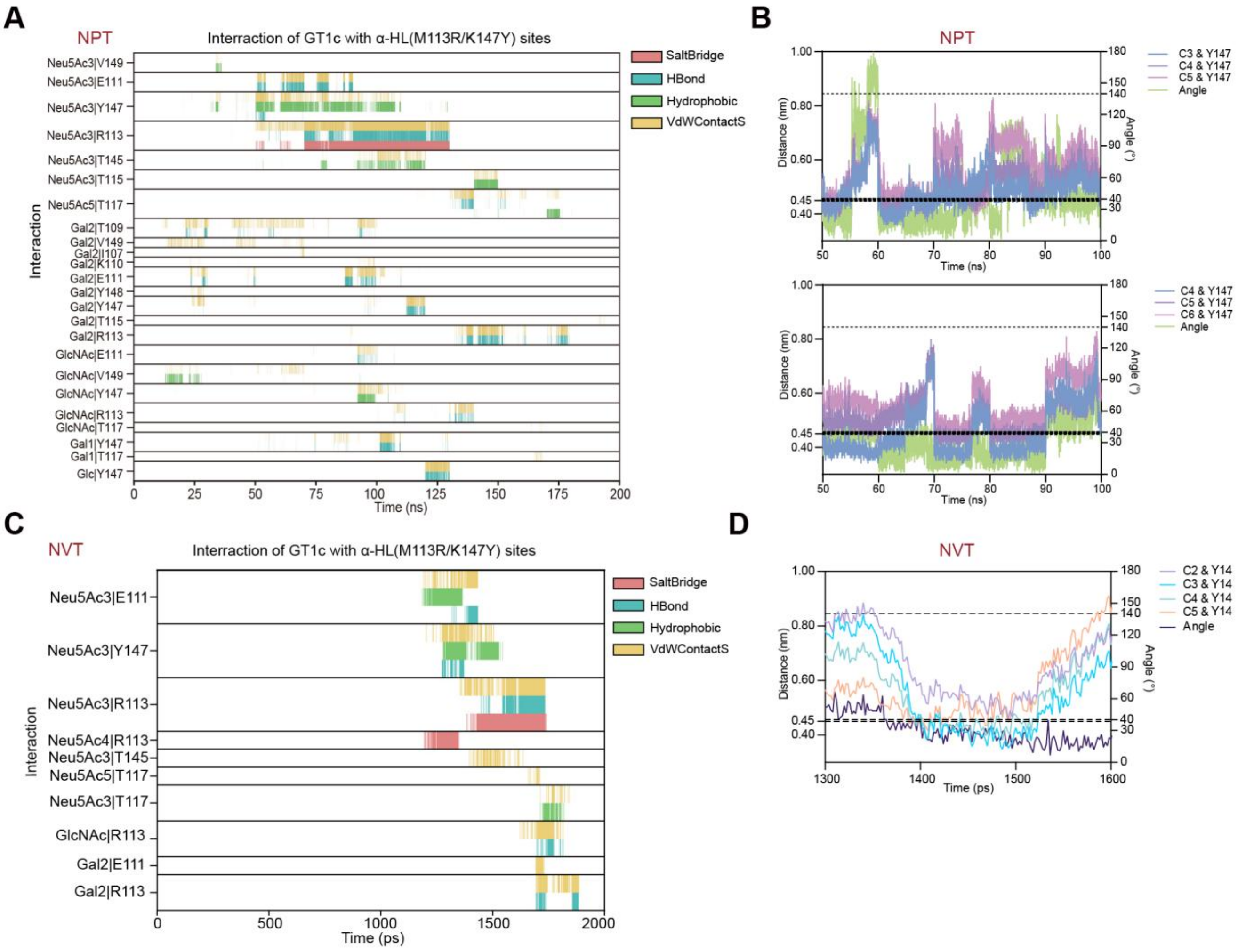

Figure S11. The temporal variation of interactions in M113R/K147Y system over the course of GT1c translocating under NPT using umbrella sampling or NVT ensemble using constant-velocity SMD.

(A) Interactions that occurred with a frequency exceeding 0.5% are displayed in M113R and M113R/K147Y systems under NPT ensemble for production, respectively. The types of interactions are denoted at the right of the figure. (B) CH–π geometry during the 50 - 100 ns interval in M113R/K147Y systems under NPT ensemble for production is shown. Distance between C-H groups of pyranose ring and COM of the tyrosine aromatic ring, and angle between pyranose ring and aromatic ring. Neu5Ac1 and Y147 of chain G (upper); Neu5Ac2 and Y147 of chain E (bottom). A distance cutoff of 0.45 nm and an angle cutoff of 40° (dashed lines) were used to define CH–π interaction. (C) Interactions that occurred with a frequency exceeding 0.5% are displayed in M113R/K147Y systems under NVT ensemble for production. The types of interactions are denoted at the right of the figure. (D) CH–π geometry during the 1300 - 1600 ps interval in M113R/K147Y systems under NVT ensemble for production is shown. Distance between the C-H groups of the Neu5Ac1 pyranose ring and the center of mass of the aromatic ring of Y147 in chain G, and angle between the pyranose ring and the aromatic ring.

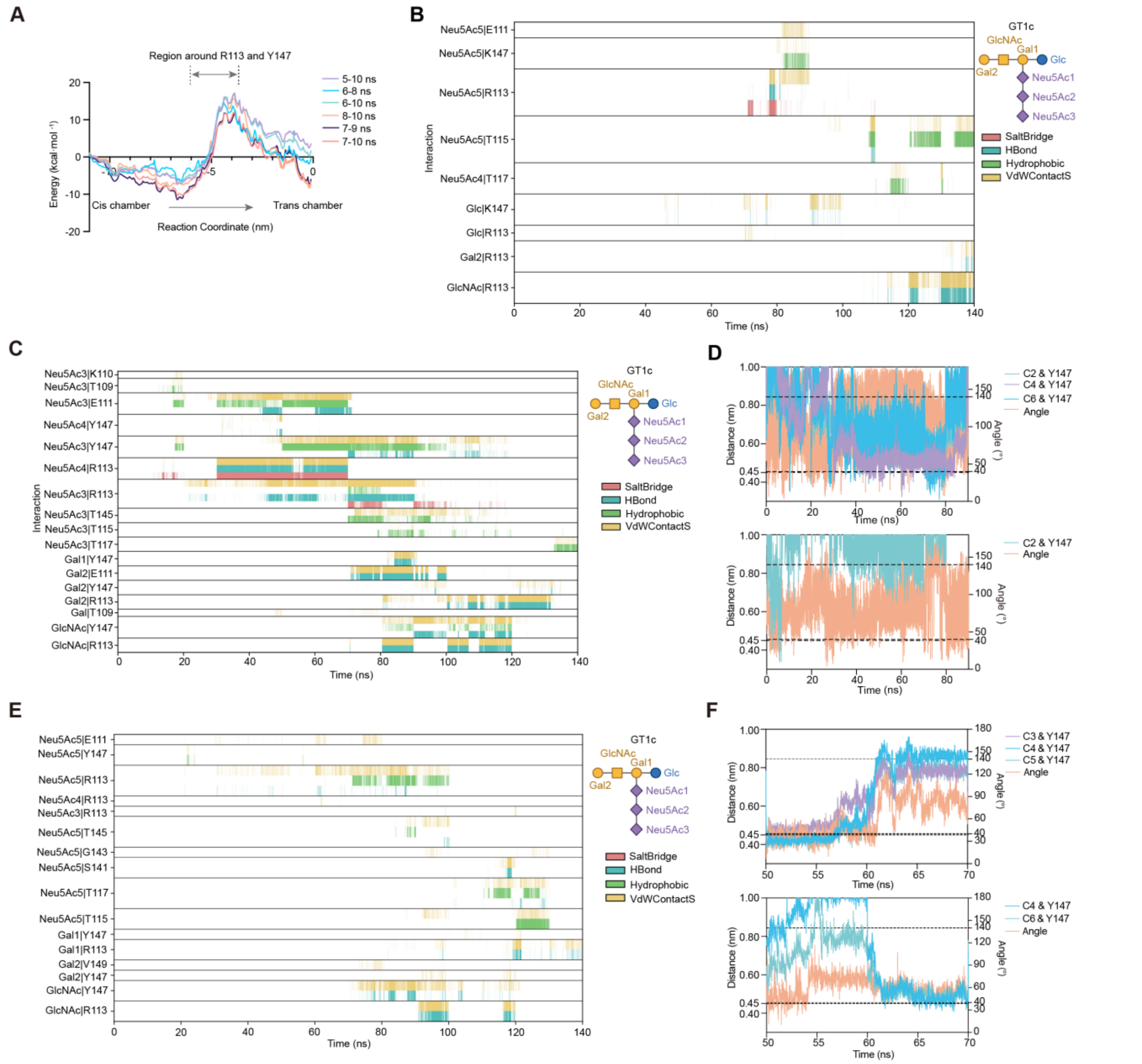

Figure S12. Analysis of different initial glycan position in M113R and M113R/K147Y systems.

(A) The PMFs are shown as a function of the coordination of Neu5Ac along the z axis from the Cis chamber to the Trans chamber of the nanopore with unchanged initial glycan position in M113R/K147Y system. Lines of different colors represent the PMF calculated over different sampling periods. The position of the largest free energy barrier is comparable in each. (B-C) The temporal variation of interactions that occurred with a frequency exceeding 0.5% are displayed over the course of GT1c translocating with the initial position aligned along the pore z-axis in M113R and M113R/K147Y systems, respectively. The types of interactions are denoted at the right of the figure. (D) CH-π geometry during the 0 - 90 ns interval is shown in M113R/K147Y system with the initial position aligned along the pore z-axis. Distance between C-H groups of pyranose ring and COM of the tyrosine aromatic ring, and angle between pyranose ring and aromatic ring. Neu5Ac3 and Y147 of chain C (top); Neu5Ac3 and Y147 of chain D (bottom). (E)The temporal variation of interactions that occurred with a frequency exceeding 0.5% are displayed in M113R/K147Y system over the course of GT1c translocating with the initial position placed off the pore z-axis. The types of interactions are denoted at the right of the figure. (F) CH-π geometry during the 50 - 70 ns interval is

shown in M113R/K147Y system with the initial position placed off the pore z-axis. Distance between C-H groups of pyranose ring and COM of the tyrosine aromatic ring, and angle between pyranose ring and aromatic ring. Neu5Ac2 and Y147 of chain B (top); Neu5Ac3 and Y147 of chain B (bottom). A distance cutoff of 0.45 nm and an angle cutoff of 40° (dashed lines) were used to define CH–π interaction.

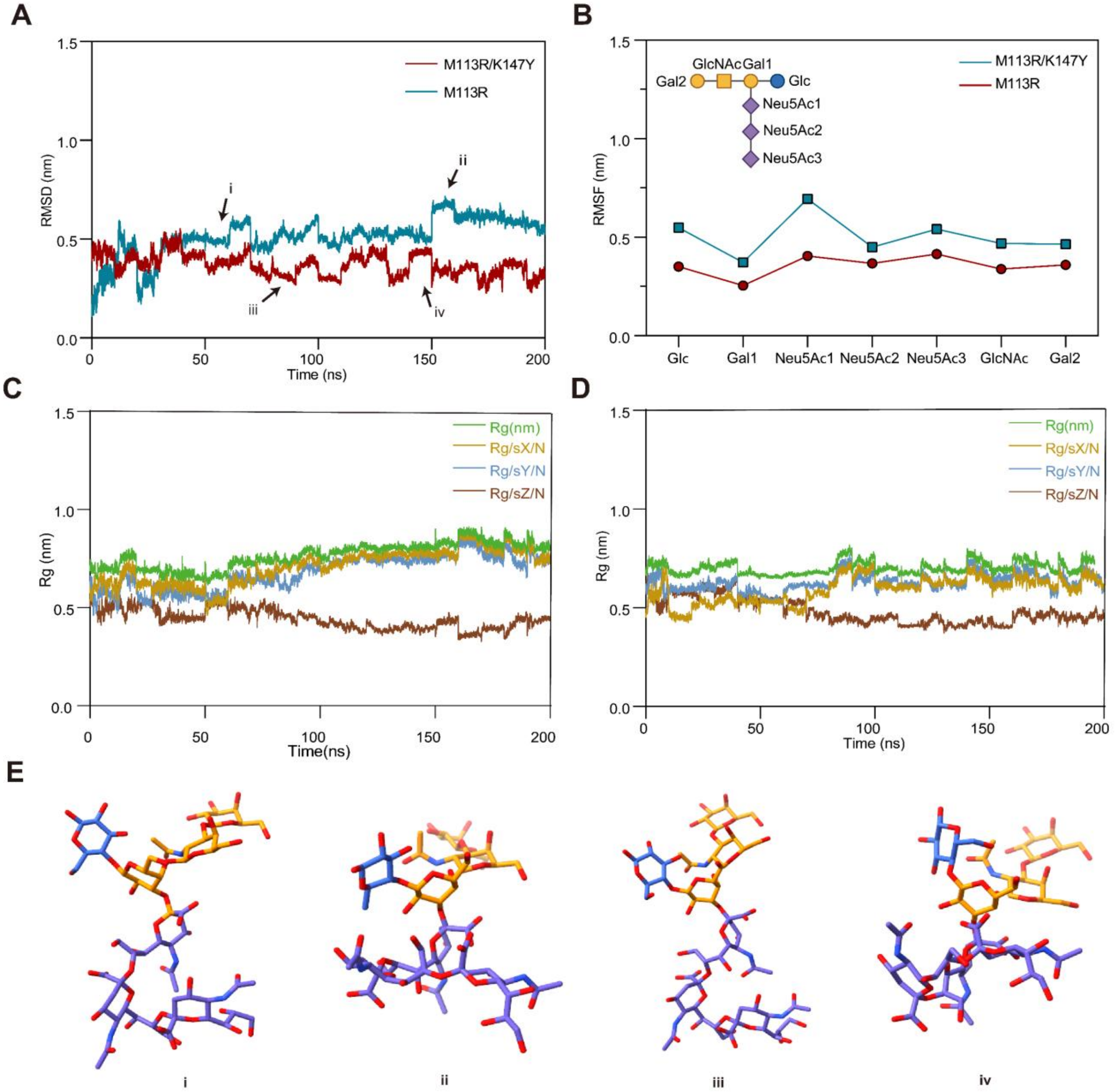

Figure S13. Analysis of GT1c conformation in M113R and M113R/K147Y systems.

(A) Time course plot for GT1c RMSD change in M113R (marked in blue) and M113R/K147Y (marked in red) systems. RMSD, root mean square deviation. Black arrows with labels i - iv denote GT1c with low- and high-RMSD in two systems, respectively. (B) GT1c RMSF in M113R (blue) and M113R/K147Y (red) systems. RMSF, root mean square fluctuation. (C, D) Time course plot for GT1c Rg change in M113R. Rg, radius of gyration. Rg/sX/N, Rg/sY/N, and Rg/sZ/N are defined as the components of Rg along the x-, y-, and z-axes, respectively. (E) Conformations i - ii and iii - iv, as indicated in (A), of GT1c in M113R and M113R/K147Y systems, respectively.

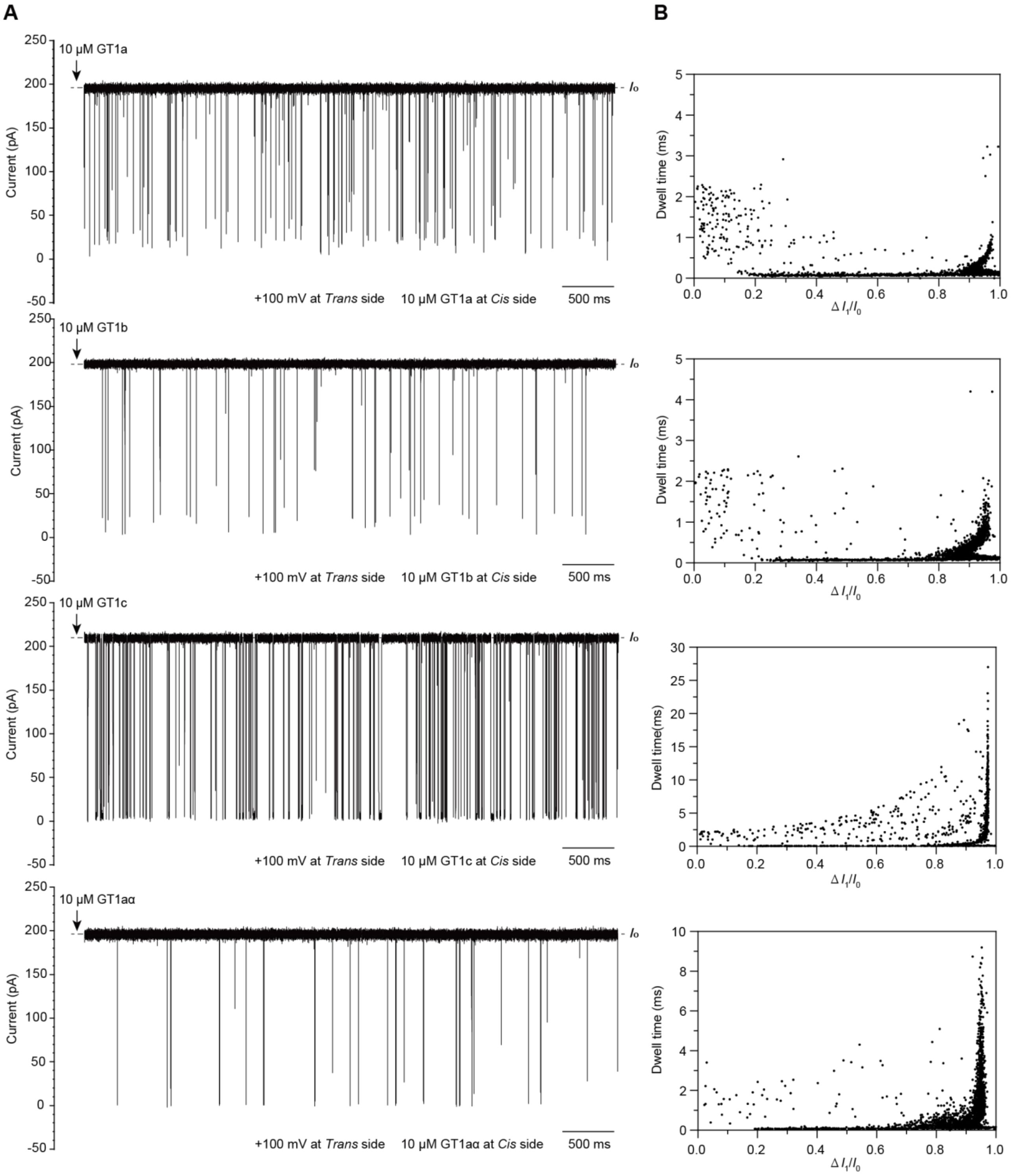

Figure S14. Detection of structural isomers using α-HL (M113R/K147Y).

(A) Representative ionic current traces of GT1a, GT1b, GT1c and GT1aα sensed by α-HL (M113R/K147Y) nanopores in a symmetric electrolyte solution of 2 M KCl (pH=5.0). (B) Scatter plots of Dwell time versus $\Delta I_1/I_0$ derived from the current blockage events extracted from the traces in (A). All nanopore experiments were performed in symmetric electrolyte solution (2 M KCl, 10 mM citric acid, pH=5.0), with the *Cis* side grounded and a potential of +100 mV applied to the *Trans* side. Each glycan was added to the *Cis* side to a final concentration of 10 μM. n ≥ 3. Each scatter plot contains at least 3000 events.

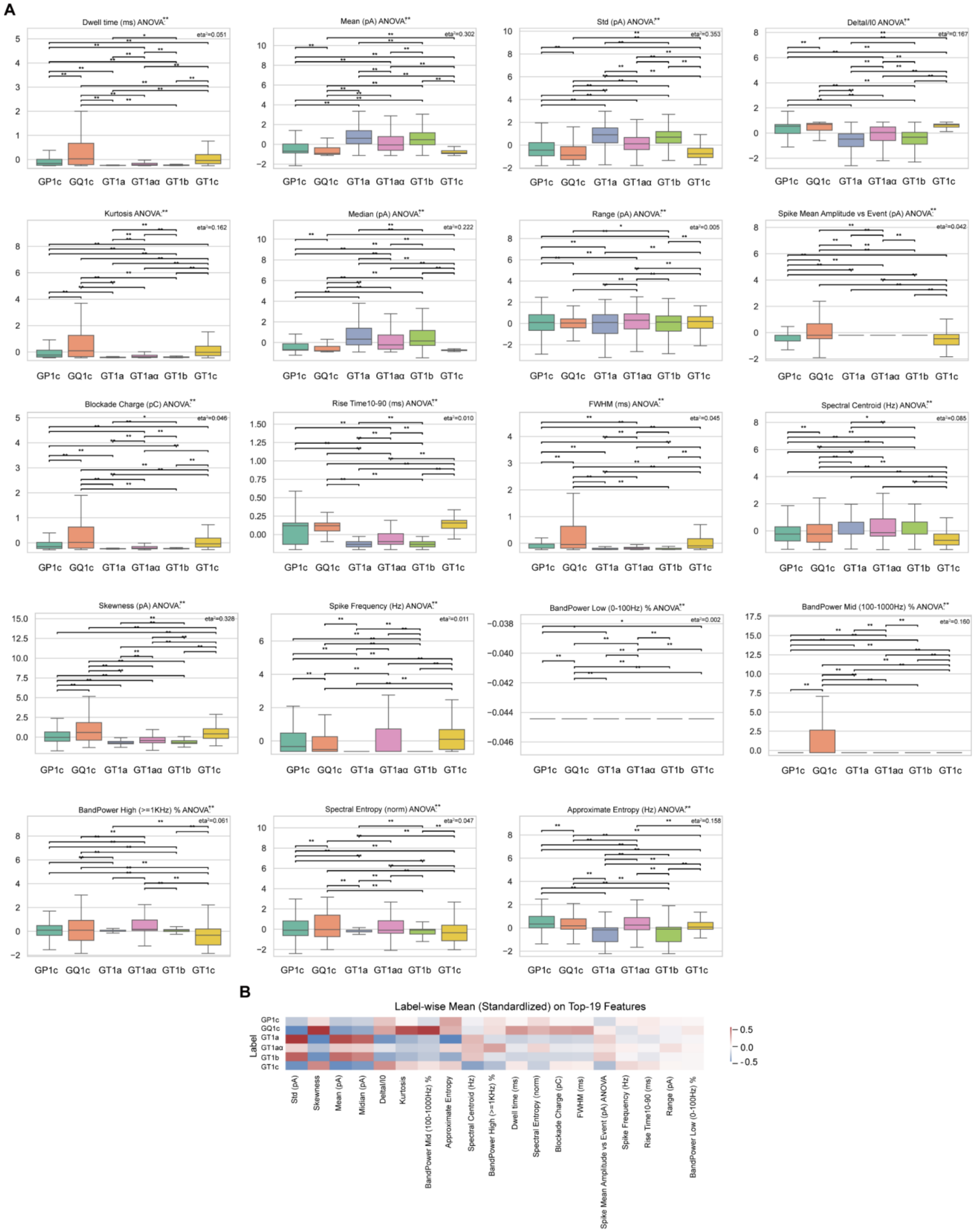

Figure S15. Statistical analysis and class-wise feature patterns of standardized nanopore event descriptors.

(A) Pairwise distribution comparison of standardized nanopore event features across analyte classes. Boxplots show the

distributions of z-score standardized feature values for each class (colors denote classes; outliers are not displayed). For each feature, a one-way ANOVA was performed across classes and the resulting *p* values were corrected for multiple testing using the Benjamini–Hochberg FDR procedure. Pairwise class comparisons were further conducted using Welch's *t*-test (two-sided) with FDR correction, and significant differences are annotated above the corresponding class pairs ($p<0.05$, "*"; $p<0.01$, "**"; *ns*, not significant). For each nanopore blockade event, a multidimensional feature set spanning time-domain, morphological, and frequency-domain characteristics was extracted. Boxplots summarize the feature distributions for the six oligosaccharide classes (GP1c, GQ1c, GT1a, GT1aα, GT1b, and GT1c). Boxes represent the interquartile range (IQR) with the median indicated by the central line; whiskers extend to 1.5×IQR. For each panel, the upper-right annotation reports the one-way ANOVA significance level and the effect size (η^2), reflecting the discriminative contribution of the corresponding feature. Only significant differences are annotated above the corresponding class pairs, not significant is not shown ($p < 0.05$, "*"; $p< 0.01$, "**"; $p< 0.001$, "***"). (B) Heatmap of class-wise mean profiles for top-ranked standardized features. Heatmap displays the class-wise mean of z-score standardized feature values for the top-ranked features (ranked by ANOVA F-statistic/weight). Rows correspond to classes and columns to features; values represent the mean standardized feature magnitude within each class, facilitating comparison of discriminative patterns across analytes.

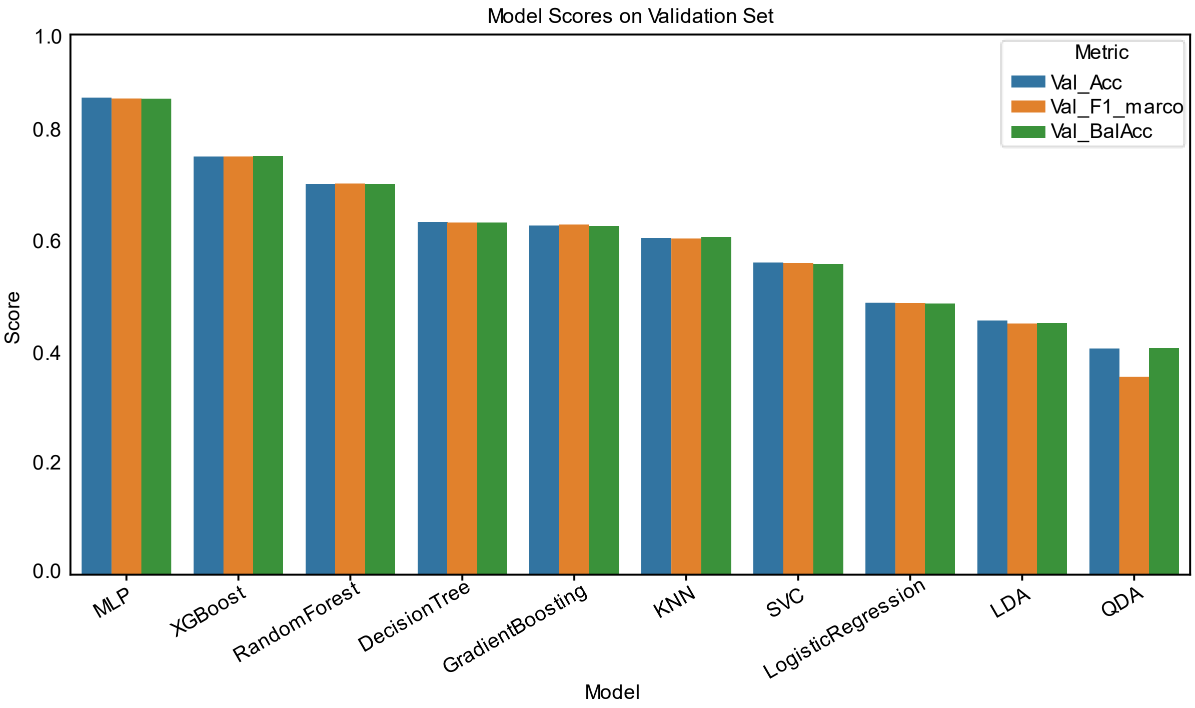

Figure S16. Validation-set performance comparison of candidate classifiers for glycan identification from nanopore event features.

Bar plot summarizes the predictive performance of multiple supervised classifiers (MLP, XGBoost, Random Forest, Decision Tree, Gradient Boosting, KNN, SVC, Logistic Regression, LDA, and QDA) evaluated on the held-out validation set. For each model, three metrics are reported: validation accuracy (Val_Acc), macro-averaged F1 score (Val_F1_macro), and balanced accuracy (Val_BalAcc). All models were trained using the same feature set and preprocessing pipeline to ensure comparability. The MLP achieved the highest overall validation performance among the tested models.

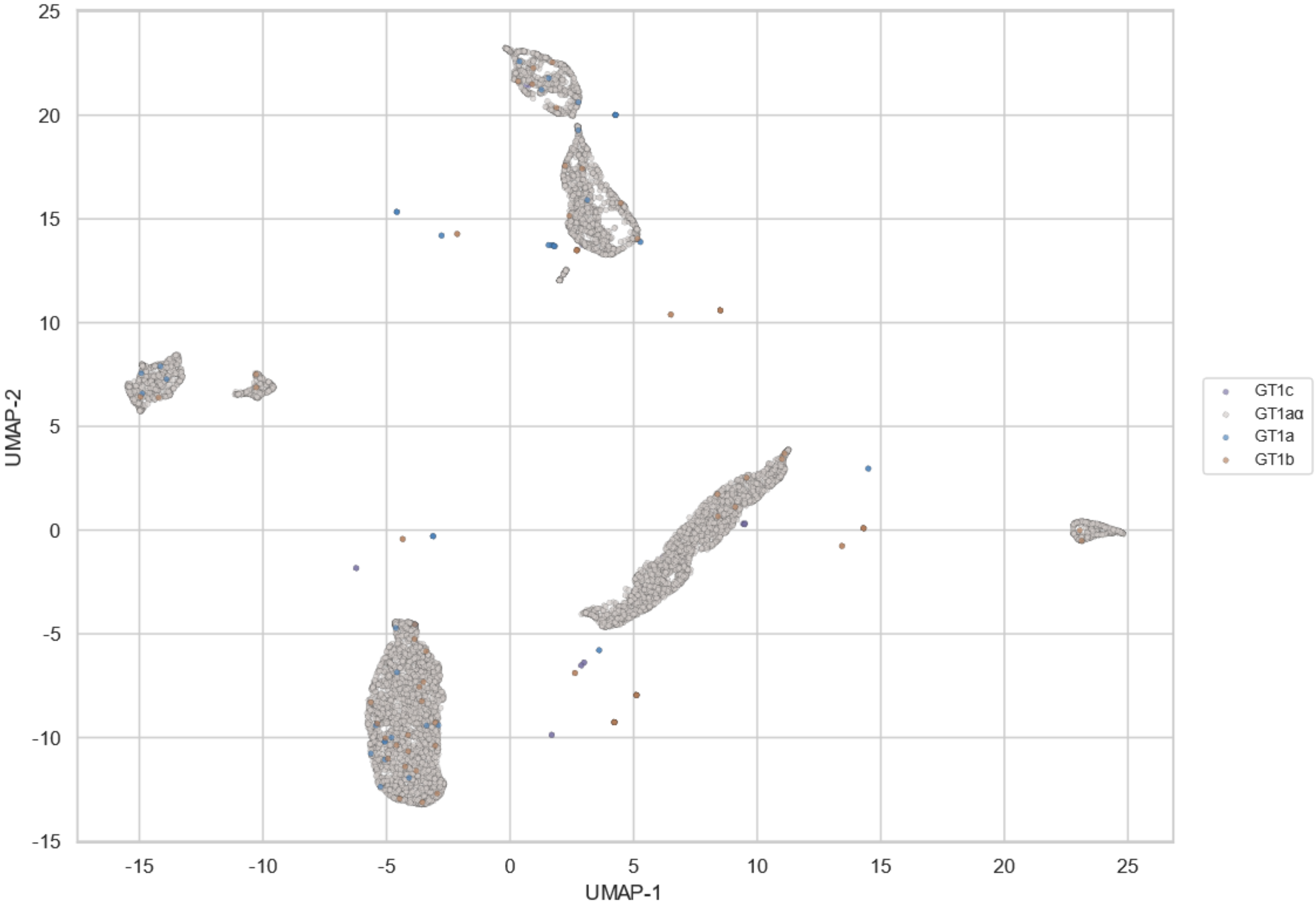

Figure S17. Single-event resolved scatter plot of structural isomers via UMAP Clustering. Dimensionality-reduced scatter plot of the nanopore data obtained from a mixture of the four ganglioside oligosaccharide isomers, processed and predicted by the machine learning model. Dimensionality-reduced scatter plot from four mixture glycan and is colored by the trained model-assigned class label. The two-dimensional embedding was generated using UMAP applied to the standardized feature vectors used for inference. The emergence of distinct, well-separated clusters corresponding to each predicted component indicates that the trained classifier can resolve multi-component mixtures at the single-event level and enables composition deconvolution in complex samples.

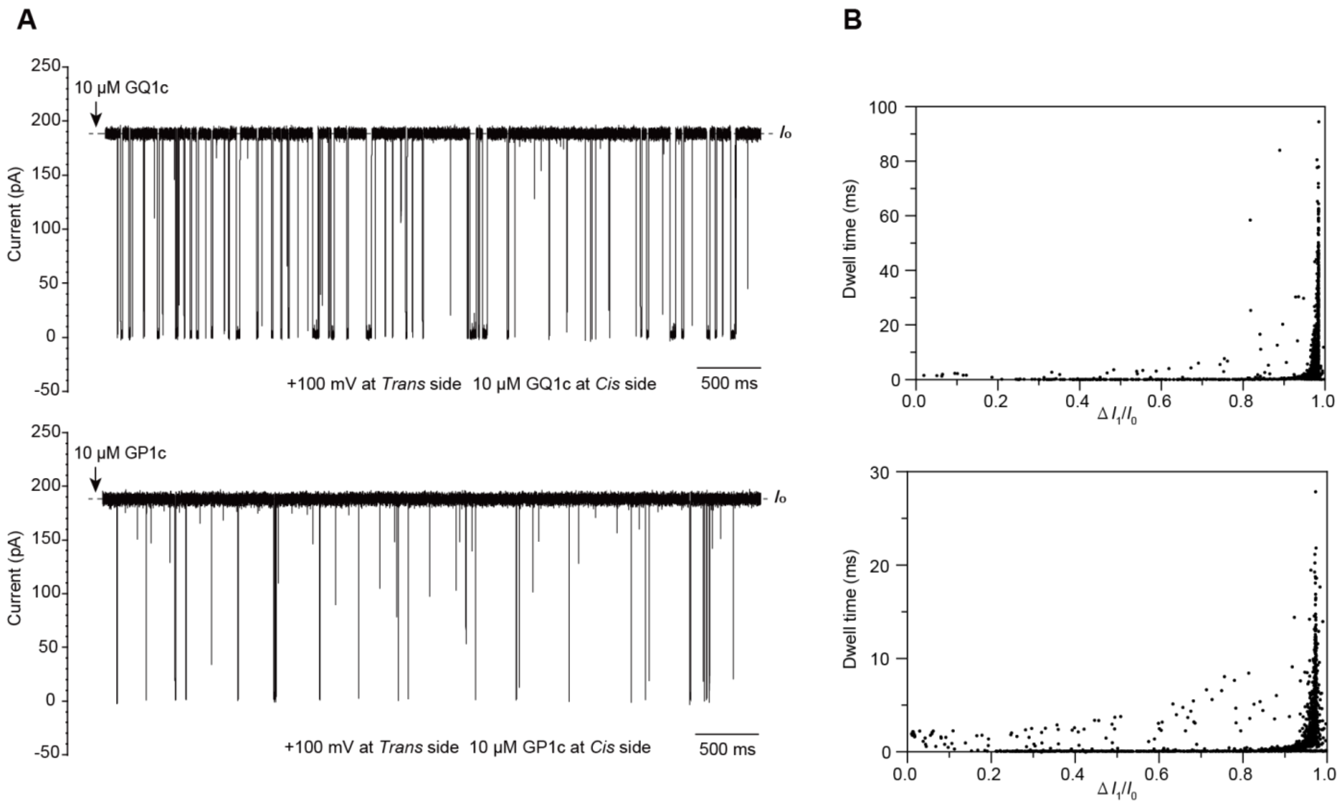

Figure S18. Detection of GQ1c and GP1c using α-HL (M113R/K147Y).

(A) Representative ionic current traces of GQ1c and GP1c sensed by α-HL (M113R/K147Y) nanopores in a symmetric electrolyte solution of 2 M KCl (pH=5.0). (B) Scatter plots of Dwell time versus $\Delta I_1/I_0$ derived from the current blockage events extracted from the traces in (A). All nanopore experiments were performed in symmetric electrolyte solution (2 M KCl, 10 mM citric acid, pH=5.0), with the *Cis* side grounded and a potential of +100 mV applied to the *Trans* side. Each glycan was added to the *Cis* side to a final concentration of 10 μM. n ≥ 3. each scatter plot contains at least 3000 events.

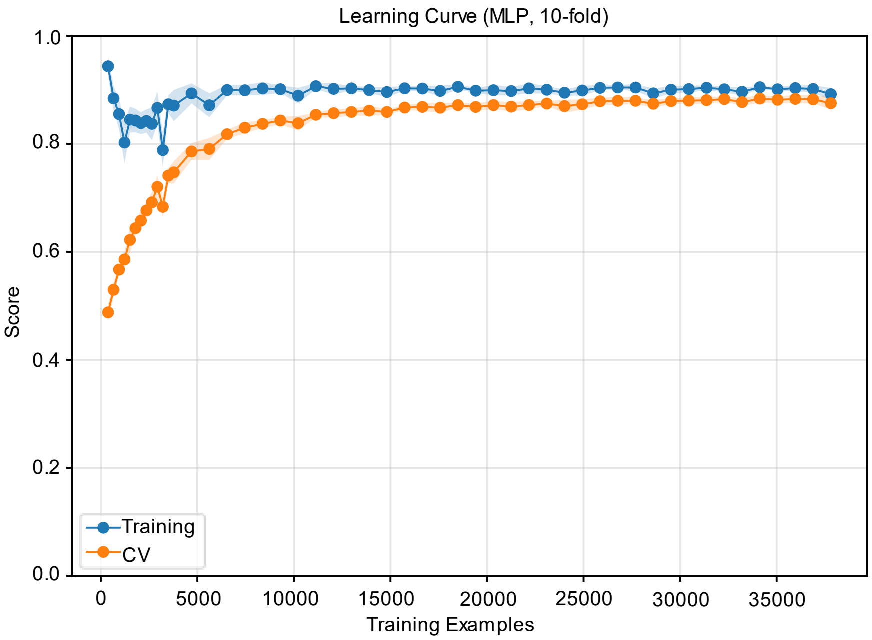

Figure S19. Learning Curve of the MLP Classifier (10-Fold Cross-Validation).

Learning curve of the MLP model showing training score and cross-validation (CV) score as a function of the number of training examples. Scores were estimated using 10-fold stratified cross-validation, and the shaded regions indicate variability across folds (mean ± standard deviation). The curve demonstrates rapid improvement with increasing sample size and approaches a plateau at larger training sizes, indicating stable generalization performance under the current feature representation and preprocessing pipeline.

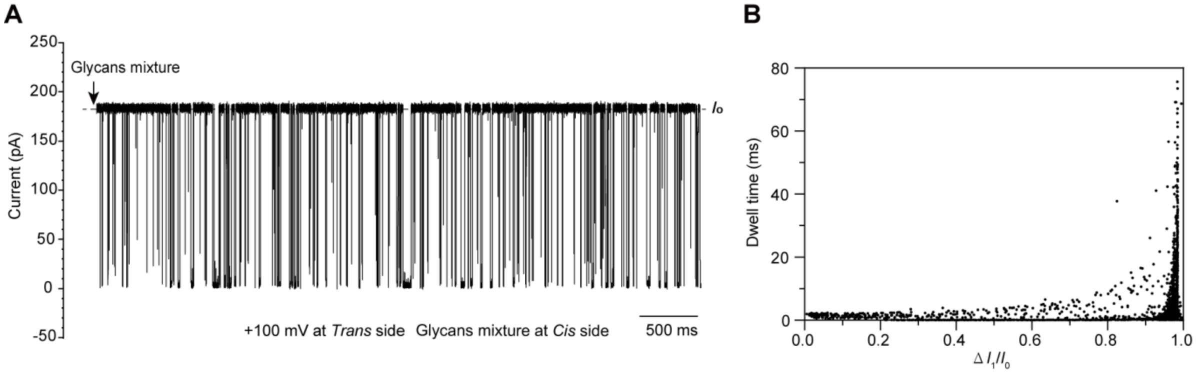

Figure S20. Detection of glycan mixture using α-HL (M113R/K147Y).

(A) Representative ionic current traces of a glycan mixture sensed by α-HL (M113R/K147Y) nanopores in a symmetric electrolyte solution of 2 M KCl (pH 5.0). The six glycans include GT1a, GT1b, GT1c, GT1aα, GQ1c, and GP1c. (B) Scatter plots of Dwell time versus $\Delta I_1/I_0$ derived from the current blockage events extracted from the traces in (A). All nanopore experiments were performed in symmetric electrolyte solution (2 M KCl, 10 mM citric acid, pH=5.0), with the *Cis* side grounded and a potential of +100 mV applied to the *Trans* side. Each glycan was added to the Cis side, each at a final concentration of 5 μM. n ≥ 3. each scatter plot contains at least 3000 events.

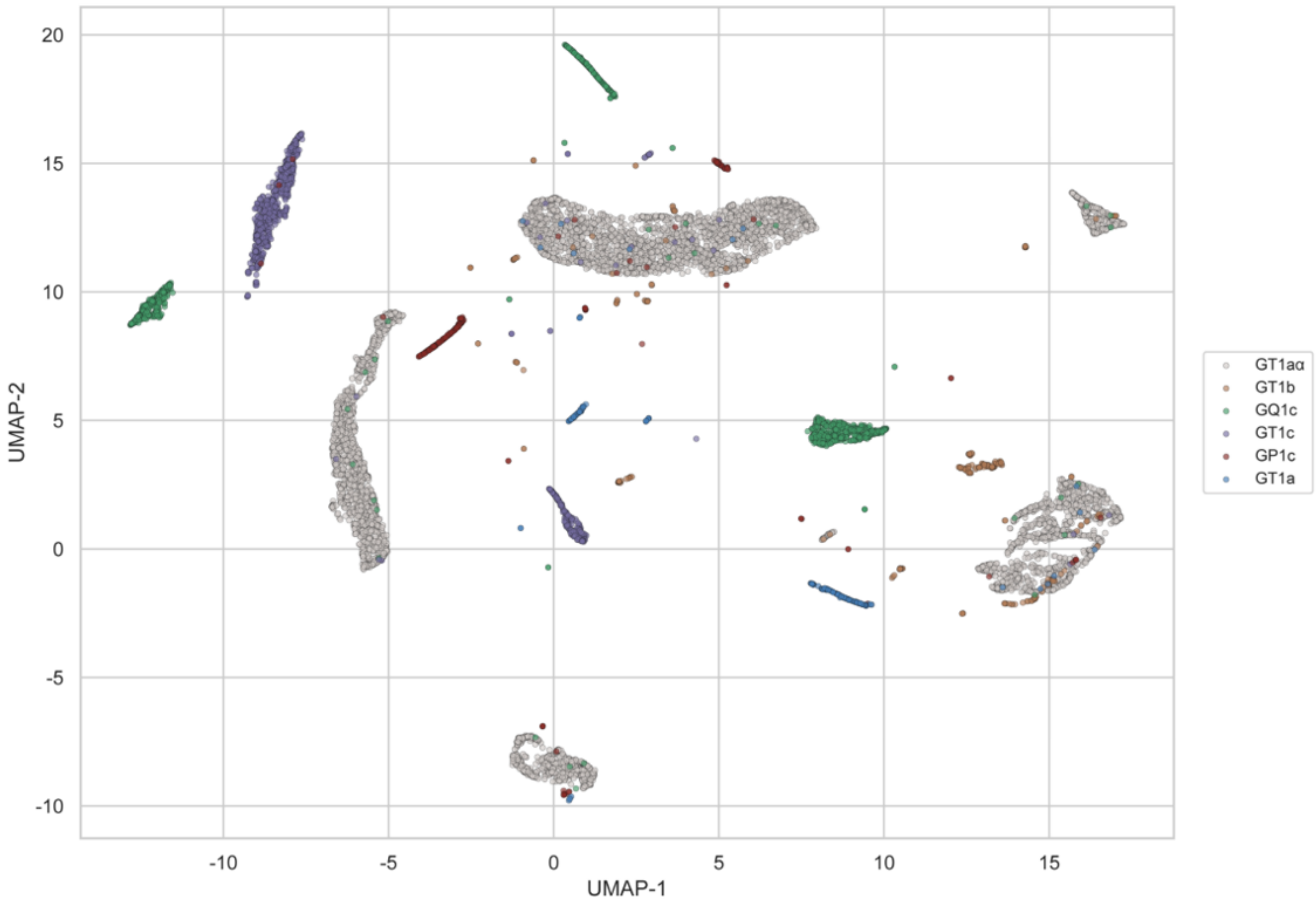

Figure S21. Single-event resolved scatter plot of six glycan mixtures via UMAP Clustering.
Dimensionality-reduced scatter plot from six mixture glycan and is colored by the trained model-assigned class label. The two-dimensional embedding was generated using UMAP applied to the standardized feature vectors used for inference. The emergence of distinct, well-separated clusters corresponding to each predicted component indicates that the trained classifier can resolve multi-component mixtures and enables composition deconvolution in complex samples.

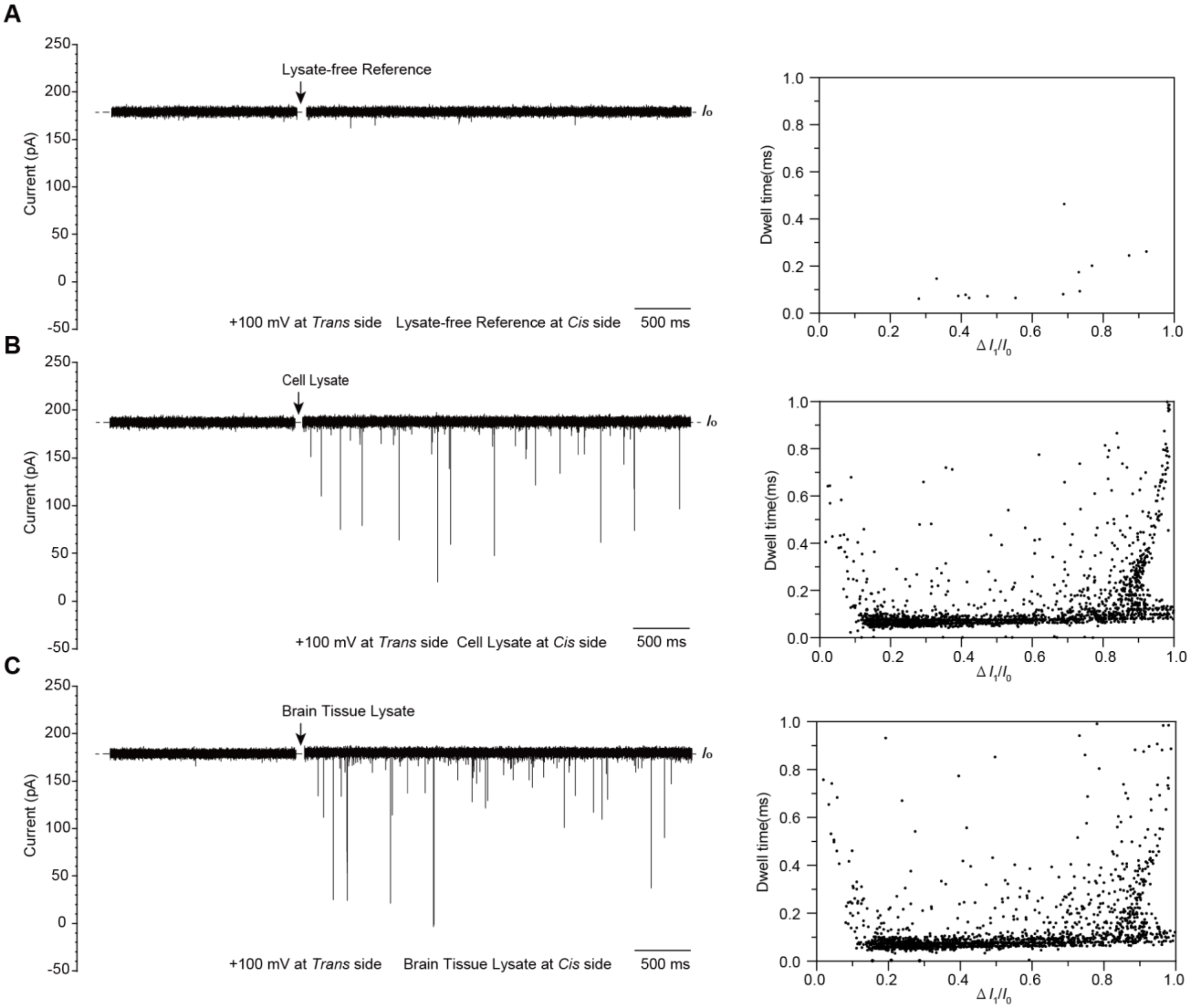

Figure S22. Detection of lysate-free referencee and lysate using α-HL (M113R/K147Y).

(A) Representative ionic current traces (left) of lysate-free reference sensed by α-HL (M113R/K147Y) nanopores in a symmetric electrolyte solution of 2 M KCl (pH=5.0). Scatter plots (right) of Dwell time versus $\Delta I_1/I_0$ derived from the current blockage events extracted from the traces. (B) Representative ionic current traces (left) of cell lysate sensed by α-HL (M113R/K147Y) nanopores in a symmetric electrolyte solution of 2 M KCl (pH=5.0). Scatter plots (right) of Dwell time versus $\Delta I_1/I_0$ derived from the current blockage events extracted from the traces. (C) Representative ionic current traces (left) of brain tissue lysate sensed by α-HL (M113R/K147Y) nanopores in a symmetric electrolyte solution of 2 M KCl (pH=5.0). Scatter plots (right) of Dwell time versus $\Delta I_1/I_0$ derived from the current blockage events extracted from the traces. All nanopore experiments were performed in symmetric electrolyte solution (2 M KCl, 10 mM citric acid, pH=5.0), with the *Cis* side grounded and a potential of +100 mV applied to the *Trans* side. Both lysates were added to the detection system at 5% (v/v). n ≥ 3. Each scatter plot corresponding to the lysate-added condition contains at least 3000 events.

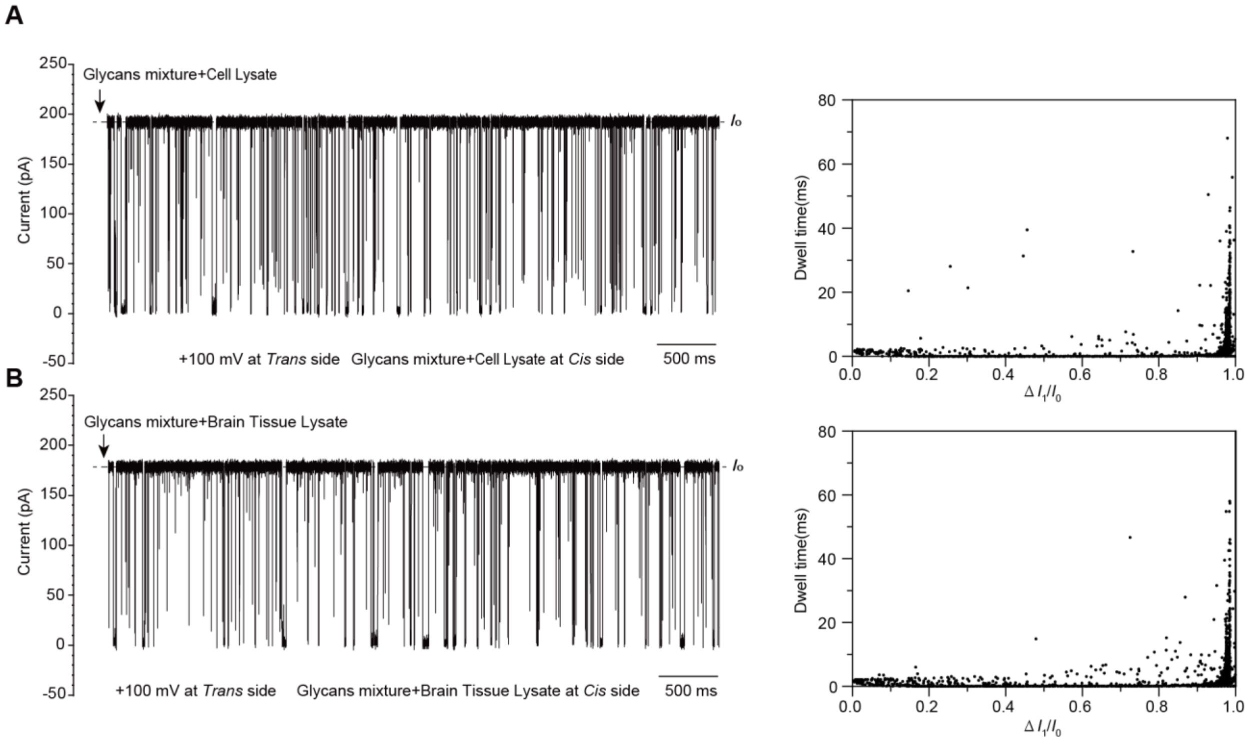

Figure S23. Detection of glycan mixture and lysate using α-HL (M113R/K147Y).

(A) Representative ionic current traces (left) of a glycan mixture and cell lysate sensed by α-HL (M113R/K147Y) nanopores in a symmetric electrolyte solution of 2 M KCl (pH=5.0). Scatter plots (right) of Dwell time versus $\Delta I_1/I_0$ derived from the current blockage events extracted from the traces. (B) Representative ionic current traces (left) of a glycan mixture and brain tissue lysate sensed by α-HL (M113R/K147Y) nanopores in a symmetric electrolyte solution of 2 M KCl (pH=5.0). Scatter plots (right) of Dwell time versus $\Delta I_1/I_0$ derived from the current blockage events extracted from the traces. All nanopore experiments were performed in symmetric electrolyte solution (2 M KCl, 10 mM citric acid, pH=5.0), with the *Cis* side grounded and a potential of +100 mV applied to the *Trans* side. Each glycan was added to the Cis side, each at a final concentration of 5 μM, and both lysates were added to the detection system at 5% (v/v). n ≥ 3. each scatter plot contains at least 3000 events.

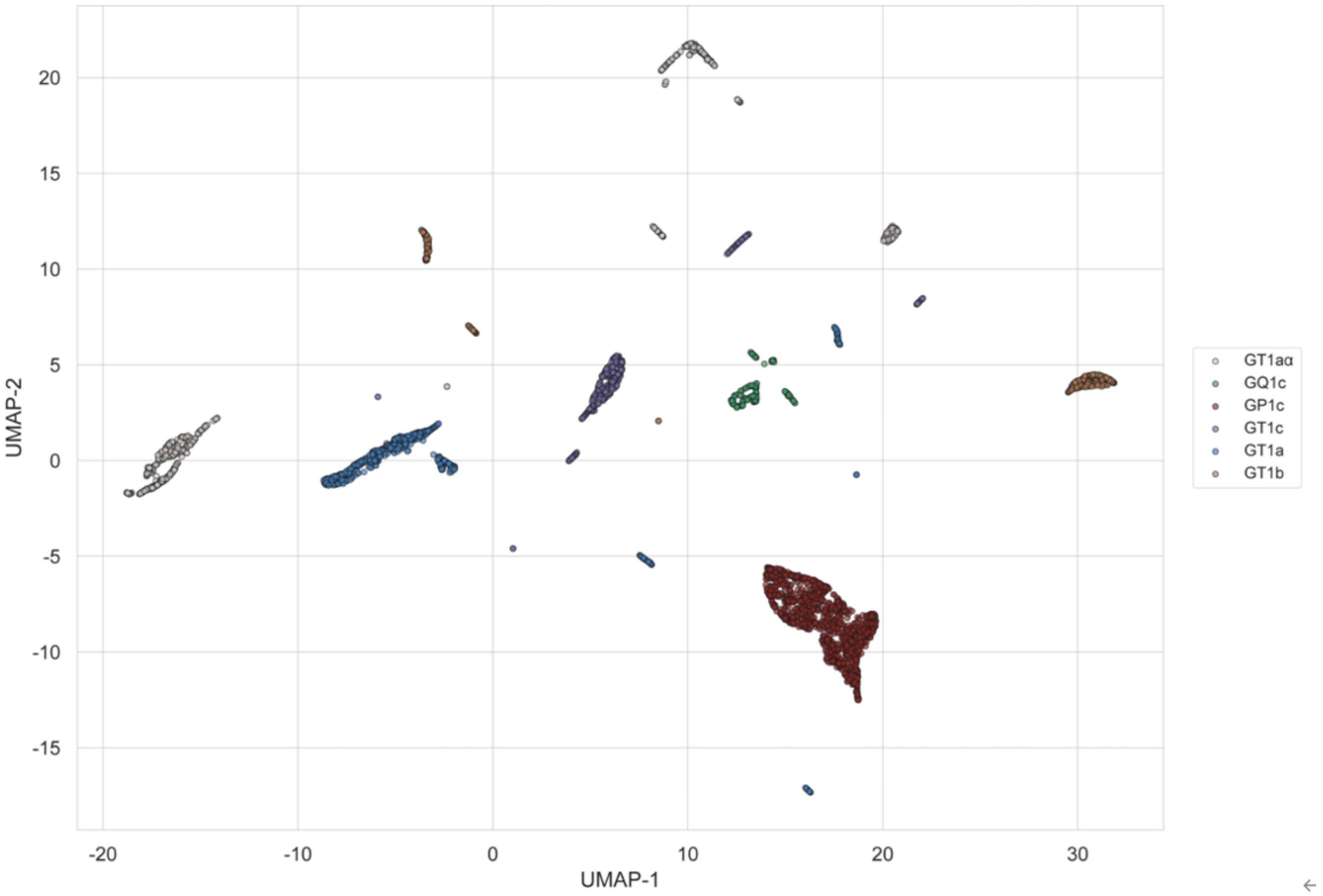

Figure S24. Single-event resolved scatter plot of six glycan mixtures in cell lysate via UMAP Clustering. Dimensionality-reduced scatter plot from six mixture glycan with interference of cell lysate and is colored by the trained model-assigned class label. The two-dimensional embedding was generated using UMAP applied to the standardized feature vectors used for inference. The emergence of distinct, well-separated clusters corresponding to each predicted component indicates that the trained classifier can resolve multi-component mixtures at the single-event level and enables composition deconvolution in complex samples.

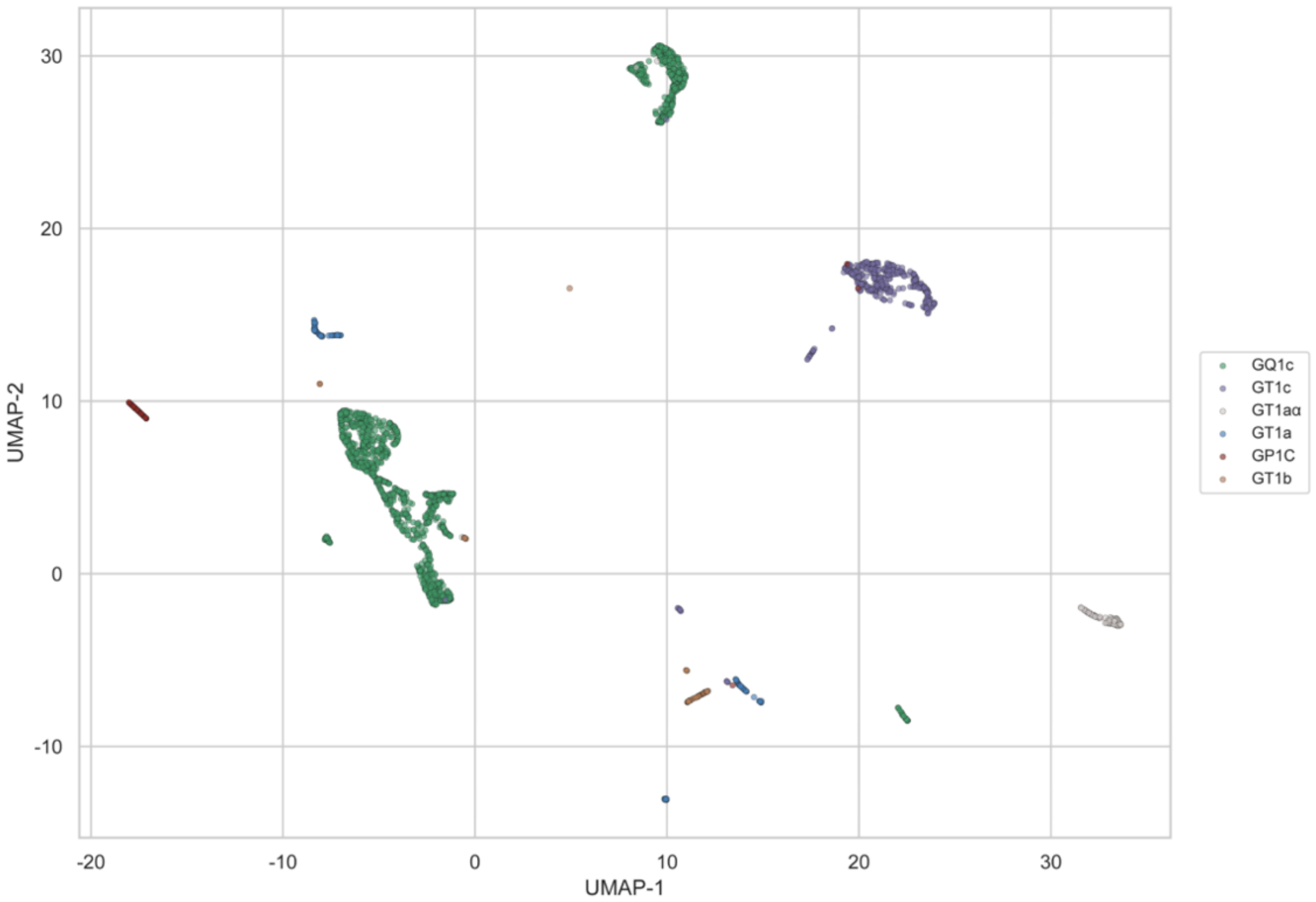

Figure S25. Single-event resolved scatter plot of six glycan mixtures in brain tissue lysate via UMAP Clustering. Dimensionality-reduced scatter plot from six mixture glycan with interference of brain tissue lysate and is colored by the trained model-assigned class label. The two-dimensional embedding was generated using UMAP applied to the standardized feature vectors used for inference. The emergence of distinct, well-separated clusters corresponding to each predicted component indicates that the trained classifier can resolve multi-component mixtures at the single-event level and enables composition deconvolution in complex samples.

2. Supporting tables

Table S1. Detection Frequency, Fitted Dwell Time, and Fitted $\Delta I_1/I_0$ of GT1c by WT and Individual Mutants.

nanopore†	Capture frequency / event s^{-1}*	Dwell time / ms*	$\Delta I_1/I_0$*
WT	6.86±0.72	0.03±0.01	0.23±0.01
M113R	45.04±1.74	0.40±0.01	0.89±0.00
T145R	21.78±4.33	0.31±0.03	0.92±0.00
T115R	29.87±2.64	0.05±0.01	0.42±0.02
G143R	9.73±2.08	0.08±0.01	0.84±0.01

†The measurement was in 3M KCl, 10 mM citric acid, under an applied voltage of +100mV, at 24±1℃.

*(Mean ± SD)

Table S2. Definitions of five current signal features.

Feature	Formula	Definition and Physical Meaning
Spectral Centroid	$\boldsymbol{f_c} = \frac{\boldsymbol{\Sigma_{k=1}^{K} f_k P_{xx}(f_k)}}{\boldsymbol{\Sigma_{k=1}^{K} P_{xx}(f_k) + \varepsilon}}$	The power spectral density (PSD)-weighted mean frequency representing the spectral "center of mass" and indicating the relative contribution of high-frequency components in the event.
BandPower Mid	$\boldsymbol{BP_{mid}}(\%) = \boldsymbol{100} \times \frac{\boldsymbol{f_{10}^{1000} P_{xx}(f)}\, \mathrm{d}\boldsymbol{f}}{\int_{0}^{f_N} \boldsymbol{P_{xx}(f)}\, \mathrm{d}\boldsymbol{f} + \boldsymbol{\varepsilon}}$	The fraction of total power within 100-1000 Hz, reflecting the contribution of mid-timescale (sub-millisecond to millisecond) fluctuations.
BandPower High	$\boldsymbol{BP_{high}}(\%) = \boldsymbol{100} \times \frac{\boldsymbol{f_{1000}^{H} P_{xx}(f)}\, \mathrm{d}\boldsymbol{f}}{\int_{0}^{f_N} \boldsymbol{P_{xx}(f)}\, \mathrm{d}\boldsymbol{f} + \boldsymbol{\varepsilon}}$ $\boldsymbol{f_H} = \mathbf{min}(\mathbf{10,000}, \boldsymbol{f_N})$	The proportion of power in the high-frequency band (⩾1kHz), indicating the strength of rapid variations, spikes, or high-frequency jitter.
Spectral Entropy	$\boldsymbol{H}_{\text{spec}} = \frac{-\sum_{k=1}^{K} \boldsymbol{p_k} \, \mathbf{ln} \boldsymbol{p_k}}{\mathbf{ln}\, \boldsymbol{K}}$	The Shannon entropy of the normalized PSD treated as a probability distribution, measuring the dispersion of spectral energy.
Approximate Entropy	$\mathbf{ApEn}(\boldsymbol{m}, \boldsymbol{r}, \boldsymbol{N}) = \boldsymbol{\phi}^{(m)}(\boldsymbol{r}) - \boldsymbol{\phi}^{(\boldsymbol{m}+\mathbf{1})}(\boldsymbol{r}).$	(ApEn) A complexity metric based on time-series similarity statistics, quantifying temporal irregularity and unpredictability of the signal.

Table S3. Fitted dwell time values for GT1c detection under voltage gradients.

Voltage[†] /mV	Dwell time / ms*
40	0.20±0.04
60	0.61±0.03
80	2.78±0.12
	0.08±0.01
100	3.19±0.18
	0.08±0.02
120	1.72±0.06
140	1.04±0.09

[†]The measurement was in 2 M KCl, 10 mM citric acid, under an applied voltage of +100 mV, at 24±1℃.

[*](Mean ± SD)

Table S4. Gaussian fitted $\Delta I_1/I_0$ values for GT1c across concentration gradients.

Glycan concentration † /μM	$\Delta I_1/I_0$*
1	0.96±0.00
5	0.96±0.00
10	0.96±0.00
20	0.96±0.00
30	0.95±0.00

†The measurement was in 2M KCl, 10 mM citric acid, under an applied voltage of +100mV, at 24±1℃.

*(Mean ± SD)

Table S5. Interactions of GT1c and residues of α-HL in M113R and M113R/K147Y systems of MD simulations. (Only the most frequently interacting residues are shown.)

Residues	R113			Y147			E111			T117	
Interaction	VdWContact	HBond	Anionic	VdWContact	HBond	Anionic	HBond	VdWContact	HBond	VdWContact	Hydrophobic
M113R	42.5%	36.84%	2.57%	8.68%	/	1.82%	3.48%	4.62%	3.25%	1.56%	3.58%
M113R/K147Y	38.96%	27.71%	27.7%	24.48%	23.29%	/	9.74%	14.74%	11.84%	4.64%	2.16%

Table S6. The pKa values calculated for titratable residues of sensing region in α-HL (M113R) and α-HL (M113R/K147Y).

Chains of α-HL	Residues	H++	PropKa	DeepKa	KaML-ESM
**M113R**					
Chain A	E111	2.5	4.00	2.57	4.17
	D127	1.3	1.96	3.33	4.30
	D128	2.8	3.01	3.39	3.81
	H144	5.8	5.24	6.48	6.20
Chain B	E111	1.6	3.55	2.85	4.18
	D127	1.4	2.45	3.31	4.32
	D128	2.5	2.17	3.40	3.82
	H144	5.6	5.13	6.62	6.21
Chain C	E111	2.7	3.96	2.44	4.17
	D127	1.0	2.47	3.29	4.33
	D128	2.5	2.21	3.61	3.80
	H144	5.7	5.20	7.17	6.21
Chain D	E111	2.2	3.23	2.47	4.18
	D127	3.6	2.51	3.45	4.32
	D128	2.4	2.22	3.42	3.81
	H144	5.3	5.26	6.57	6.22
Chain E	E111	2.0	4.35	2.61	4.18
	D127	1.9	1.96	3.19	4.29
	D128	2.6	3.20	3.37	3.80
	H144	5.7	5.02	6.41	6.23
Chain F	E111	1.6	3.71	2.55	4.17
	D127	1.6	2.11	3.21	4.31
	D128	3.1	3.08	3.67	3.81
	H144	5.1	5.27	6.19	6.21
Chain G	E111	1.8	3.08	2.54	4.18
	D127	1.4	1.99	3.59	4.31
	D128	3.1	3.22	3.28	3.81
	H144	5.7	5.31	6.39	6.21
**M113R/K147Y**					
Chain A	E111	3.3	4.81	4.17	4.18
	D127	1.4	1.95	3.45	4.31
	D128	2.7	3.03	3.51	3.81

	H144	5.8	5.19	6.30	6.20
Chain B	E111	3.6	4.86	4.05	4.18
	D127	1.4	2.46	3.41	4.31
	D128	2.4	2.17	3.48	3.80
	H144	5.8	5.08	6.60	6.22
Chain C	E111	3.6	4.78	3.66	4.17
	D127	1.1	2.47	3.37	4.31
	D128	2.4	2.21	3.22	3.81
	H144	5.8	5.20	6.77	6.20
Chain D	E111	2.7	4.53	3.80	4.18
	D127	3.6	2.51	3.66	4.30
	D128	2.6	2.22	3.33	3.81
	H144	5.9	5.23	6.46	6.21
Chain E	E111	3.9	4.53	4.07	4.18
	D127	1.6	1.96	3.44	4.31
	D128	2.9	3.20	3.24	3.81
	H144	5.8	4.94	6.69	6.21
Chain F	E111	4.1	4.90	3.80	4.17
	D127	1.2	2.10	3.72	4.32
	D128	3.1	3.09	3.09	3.82
	H144	5.6	5.19	6.39	6.21
Chain G	E111	2.3	4.41	3.76	4.18
	D127	1.4	1.99	3.35	4.31
	D128	3.1	3.22	3.30	3.81
	H144	5.5	5.05	6.72	6.21

3. Definition and formulas for the six features.

1. Spectral Centroid (Hz)

Definition: The spectral centroid is the "center of mass" of the spectrum, computed as the PSD-weighted mean frequency

$$f_c = \frac{\Sigma_{k=1}^{K} f_k P_{xx}(f_k)}{\Sigma_{k=1}^{K} P_{xx}(f_k) + \varepsilon}$$

where $\{f_k\}_{k=1}^{K}$are the Welch frequency bins, $P_{xx}(f_k)$is the PSD value at f_k, and $\varepsilon$is a small constant for numerical stability (here $\varepsilon = 10^{-12}$).

Interpretation (nanopore events). Larger $f_c$indicates that the event contains relatively more high-frequency content (sharper transitions, faster fluctuations, or spike-like components), whereas smaller $f_c$suggests slower, smoother dynamics dominated by low-frequency components.

2. BandPower Mid (100–1000 Hz) %

Definition: Relative mid-band power is the fraction of total PSD power lying between 100 and 1000 Hz:

$$BP_{mid}(\%) = 100 \times \frac{f_{10}^{1000} P_{xx}(f)\, \mathrm{d}f}{\int_0^{f_N} P_{xx}(f)\, \mathrm{d}f + \varepsilon}$$

In implementation, the integrals are approximated by trapezoidal numerical integration over the discrete Welch frequency grid.

Interpretation. $\mathbf{BP}_{\mathrm{mid}}$summarizes how strongly the event's energy is concentrated in mid-frequency dynamics (e.g., sub-millisecond to millisecond-scale fluctuations) relative to the overall event power. As a percentage feature, it is less sensitive to absolute event amplitude scaling than raw power.

3. BandPower High (≥ 1 kHz) %

Definition: elative high-band power is computed as:

$$BP_{high}(\%) = 100 \times \frac{f_{1000}^{H} P_{xx}(f)\, \mathrm{d}f}{\int_0^{f_N} P_{xx}(f)\, \mathrm{d}f + \varepsilon}$$

$$f_H = \min(10{,}000, f_N)$$

The upper limit $f_H$enforces the Nyquist constraint and, when $f_s > 20$kHz, caps the high band at 10 kHz to keep the definition consistent across recordings.

Interpretation. Larger $\mathbf{BP}_{\mathrm{high}}$indicates stronger fast components (rapid edges, high-frequency jitter, or transient spikes) within the event waveform. When comparing across datasets, this feature is only meaningful if $f_s$is sufficiently high such that $f_N \geq 1$kHz.

4. Spectral Entropy (normalized)

Definition. Spectral entropy treats the normalized PSD as a discrete probability distribution over frequency and computes Shannon entropy. Let

$$p_k = \frac{P_{xx}(f_k) + \varepsilon}{\Sigma_{j=1}^{k} l P_{xx}(f_i) + \varepsilon}$$

Then the normalized spectral entropy is

$$H_{\mathrm{spec}} = \frac{-\sum_{k=1}^{K} p_k \ln p_k}{\ln K}$$

so that $\mathbf{H}_{\text{spec}} \in [\mathbf{0}, \mathbf{1}]$

Interpretation.

- $\boldsymbol{H}_{\text{spec}} \to \mathbf{0}$: power concentrated in a narrow frequency region (more "tonal" / structured spectrum).
- $\boldsymbol{H}_{\text{spec}} \to \mathbf{1}$: power spread broadly across frequencies (more "noise-like" / broadband spectrum).

  For nanopore events, higher spectral entropy typically reflects more heterogeneous fluctuations within the event waveform.

5. Approximate Entropy (ApEn)

Definition. Approximate entropy quantifies temporal irregularity/unpredictability in a time series and was introduced by Pincus. For an event waveform $\boldsymbol{x}[\mathbf{1}], \dots, \boldsymbol{x}[\boldsymbol{N}]$, define $\boldsymbol{m}$-length embedded vectors

$$\mathbf{u}_{\boldsymbol{i}}^{(\boldsymbol{m})} = \left[\boldsymbol{x}[\boldsymbol{i}], \boldsymbol{x}[\boldsymbol{i}+\mathbf{1}], \dots, \boldsymbol{x}[\boldsymbol{i}+\boldsymbol{m}-\mathbf{1}]\right], \boldsymbol{i} = \mathbf{1}, \dots, \boldsymbol{N}-\boldsymbol{m}+\mathbf{1}$$

Define the Chebyshev (maximum) distance

$$\boldsymbol{d} \quad \left(\mathbf{u}_{\boldsymbol{i}}^{(\boldsymbol{m})}, \mathbf{u}_{\boldsymbol{j}}^{(\boldsymbol{m})}\right) = \max_{\mathbf{0} \le \boldsymbol{\ell} < \boldsymbol{m}} \mid \boldsymbol{x}[\boldsymbol{i}+\boldsymbol{\ell}] - \boldsymbol{x}[\boldsymbol{j}+\boldsymbol{\ell}] \mid.$$

For a tolerance $\boldsymbol{r} > \mathbf{0}$, compute

$$\boldsymbol{C}_{\boldsymbol{i}}^{(\boldsymbol{m})}(\boldsymbol{r}) = \frac{\mathbf{1}}{\boldsymbol{N}-\boldsymbol{m}+\mathbf{1}} \# \left\{\boldsymbol{j} \in [\mathbf{1}, \boldsymbol{N}-\boldsymbol{m}+\mathbf{1}] : \boldsymbol{d}(\mathbf{u}_{\boldsymbol{i}}^{(\boldsymbol{m})}, \mathbf{u}_{\boldsymbol{j}}^{(\boldsymbol{m})}) \le \boldsymbol{r}\right\}.$$

Then

$$\boldsymbol{\phi}^{(\boldsymbol{m})}(\boldsymbol{r}) = \frac{\mathbf{1}}{\boldsymbol{N}-\boldsymbol{m}+\mathbf{1}} \sum_{\boldsymbol{i}=\mathbf{1}}^{\boldsymbol{N}-\boldsymbol{m}+\mathbf{1}} \ln\left(\boldsymbol{C}_{\boldsymbol{i}}^{(\boldsymbol{m})}(\boldsymbol{r}) + \boldsymbol{\varepsilon}\right),$$

and approximate entropy is

$$\mathbf{ApEn}(\boldsymbol{m}, \boldsymbol{r}, \boldsymbol{N}) = \boldsymbol{\phi}^{(\boldsymbol{m})}(\boldsymbol{r}) - \boldsymbol{\phi}^{(\boldsymbol{m}+\mathbf{1})}(\boldsymbol{r}).$$

Parameterization used here. $\boldsymbol{m} = \mathbf{2}$, and $\boldsymbol{r} = \mathbf{0.2}\,\boldsymbol{\sigma}$, where $\boldsymbol{\sigma}$is the sample standard deviation of the event waveform (computed within-event). If $\boldsymbol{N} < \boldsymbol{m} + \mathbf{2}$, ApEn is reported as NaN (insufficient samples).

Interpretation. Larger ApEn indicates greater irregularity and lower short-range predictability (more complex, less repetitive dynamics). Smaller ApEn indicates more regular, repeatable patterns within the event.

Short-event handling: PSD features require a minimum event length (here $\boldsymbol{N} \ge \mathbf{8}$); otherwise features are NaN. ApEn requires $\boldsymbol{N} \ge \boldsymbol{m} + \mathbf{2}$.

Numerical stability: $\boldsymbol{\varepsilon} = \mathbf{10^{-12}}$is added where needed to avoid division by zero or $\ln(\mathbf{0})$.

Comparability across sampling rates: All frequency features explicitly respect Nyquist $\boldsymbol{f_N} = \boldsymbol{f_s}/\mathbf{2}$; $\mathbf{BP}_{\text{high}}$is truncated when $\boldsymbol{f_N} < \mathbf{10}$kHz.

4. Synthesis and characterization of Ganglioside Oligosaccharides

(1) General materials

Proton nuclear magnetic resonance (^{1}H-NMR) spectra were recorded on a Bruker Avance 600 (at 600 MHz). Multiplicities were given as singlet (s), doublet (d), doublet of doublets (dd), triplet (t), doublet of triplets (dt), or multiplet (m). Spectra were assigned using ^{1}H-NMR, COSY, HSQC and TOCSY experiments. ESI-MS data was recorded on a Shimadzu LC-MS2020. Size-exclusion chromatography was performed on a Bio-Gel P-2 or P-4 (45-90 μm) column. Cytidine-5′-monophospho-*N*-acetylneuraminic acid (CMP-Neu5Ac) and uridine 5′-diphosphogalactose (UDP-Gal) were purchased from BioChemSyn. Calf Intestine alkaline phosphatase (CIAP, M0525S) was purchased from BioLabs Inc. *Photobacterium phosphoreum* α2,3-sialyltransferase (*Pp*α2,3siaT), *Campylobacter jejuni* β1,3-galactosyltransferase (CgtB) and *Campylobacter jejuni* sialyltransferase II (Cst-II) were expressed in *E. coli* system and purified as described previously.[35] Mammalian sialyltransferases ST6GalNAc5 was expressed as soluble, secreted fusion proteins by transient transfection of HEK293 suspension cultures and purified as described previously.[36]

(2) Enzymatic Synthesis methods of ganglioside glycans

Compound GT1a

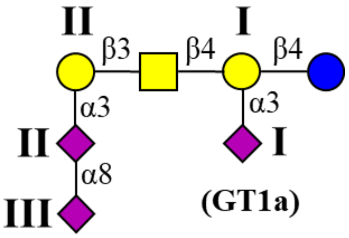

(GT1a)

To a solution of compound GD1a [36] (3.7 mg, 2.7 μmol), CMP-Neu5Ac (2.7 mg, 4.1 μmol) and $MgCl_2$ (20 mM) in Tris-HCl buffer (100 mM, pH=8.0) Cst-II (0.1 mg/mL) was added. Subsequently, the reaction is monitored by ESI-MS every 1 h until optimal conversion was achieved. The reaction mixture was centrifuged, and the resulting supernatant was purified by size-exclusion chromatography (BioGel P-2, 45-90 μm, eluent: 0.1 M NH_4HCO_3). The fractions containing the product were combined and lyophilized to afford compound GT1a as a white amorphous solid (3.5 mg, 77%). ^{1}H NMR (600 MHz, D_2O) δ 4.76 (d, J = 8.7 Hz, 1H, H1-GalNAc), 4.61 (d, J = 7.9 Hz, 1H, H1-Gal-II), 4.54 – 4.50 (m, 2H, H1-Gal-I, H1-Glc), 4.17 – 4.11 (m, 4H), 4.10 – 4.04 (m, 3H), 4.03 – 3.95 (m, 3H), 3.88 (t, J = 10.7 Hz, 4H), 3.85 – 3.80 (m, 6H), 3.80 – 3.70 (m, 13H), 3.67 (dd, J = 10.3, 7.9 Hz, 4H), 3.64 – 3.58 (m, 6H), 3.56 – 3.49 (m, 2H), 3.39 (dd, J = 9.7, 7.9 Hz, 1H), 3.32 (t, J = 8.7 Hz, 1H), 3.16 (t, J = 6.9 Hz, 2H), 2.80 (dd, J = 12.6, 4.6 Hz, 1H, $H3_{(eq)}$-Neu5Ac-III), 2.69 (dd, J = 12.6, 4.7 Hz, 1H, $H3_{(eq)}$-Neu5Ac-I), 2.65 (dd, J = 12.5, 4.5 Hz, 1H, $H3_{(eq)}$-Neu5Ac-II), 2.06 (s, 3H, CH_3CONH), 2.04 – 2.01 (m, 11H, -CH_2-, 3 × CH_3CONH), 1.91 (t, J = 11.9 Hz, 1H, $H3_{(ax)}$-Neu5Ac-I), 1.82 (t, J = 12.1 Hz, 1H, $H3_{(ax)}$-Neu5Ac-II), 1.75 (t, J = 12.2 Hz, 1H, $H3_{(ax)}$-Neu5Ac-III). ESI-MS calcd for $C_{62}H_{101}N_5O_{45}^{2-}$ $[M-2H]^{2-}$ 817.79, found 817.95.

Compound GT1aα

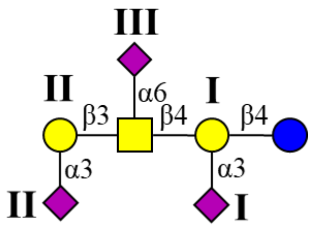

(GT1aα)

To a solution of compound GD1a [36] (2.6 mg, 1.8 μmol), CMP-Neu5Ac (2.0 mg, 2.8 μmol) and $MgCl_2$ (10 mM) in Tris-HCl buffer (100 mM, pH=7.0) was ST6GalNAc5 (0.1 mg/mL) and calf intestine alkaline phosphatase (CIAP, 2 U). Subsequently, the reaction is monitored by ESI-MS every 6 h until optimal conversion was achieved. The reaction mixture was centrifuged, and the resulting supernatant was purified by size-exclusion chromatography (BioGel P-2, 45−90 μm, eluent: 0.1 M NH_4HCO_3). The fractions containing the product were combined and lyophilized to afford compound GT1aα as a white amorphous solid (2.4 mg, 74%). ^{1}H NMR (600 MHz, D_2O) δ 4.72 (d, J = 8.6 Hz, 1H, H1-GalNAc), 4.60 (d, J = 7.9 Hz, 1H, H1-Gal-II), 4.55 – 4.51 (m, 2H, H1-Gal-I, H1-Glc), 4.19 (d, J = 3.1 Hz, 1H), 4.17 – 4.07 (m, 3H), 4.06 – 3.97 (m, 3H), 3.95 (d, J = 3.2 Hz, 1H), 3.92 – 3.87 (m, 4H), 3.85 – 3.77 (m, 8H), 3.75 – 3.71 (m, 3H), 3.68 – 3.60 (m, 9H), 3.57 – 3.50 (m, 3H), 3.37 (dd, J = 9.6, 7.9 Hz, 1H), 3.35 – 3.30 (m, 1H), 3.14 (t, J = 6.9 Hz, 2H), 2.75 (dd, J = 12.5, 4.6 Hz, 1H, $H3_{(eq)}$-Neu5Ac-II), 2.71 (dd, J = 12.4, 4.6 Hz, 1H, $H3_{(eq)}$-Neu5Ac-III), 2.67 (dd, J = 12.5, 4.6 Hz, 1H, $H3_{(eq)}$-Neu5Ac-I), 2.07 – 1.97 (m, 14H, -CH_2-, 4 × CH_3CONH), 1.96 – 1.90 (m, 1H, $H3_{(ax)}$-Neu5Ac-I), 1.80 (t, J = 12.1 Hz, 1H, $H3_{(ax)}$-Neu5Ac-II), 1.64 (t, J = 12.1 Hz, 1H, $H3_{(ax)}$-Neu5Ac-III). ESI-MS calcd for $C_{62}H_{101}N_5O_{45}{}^{2-}$ $[M-2H]^{2-}$ 817.79, found 818.05.

Compound GT1b

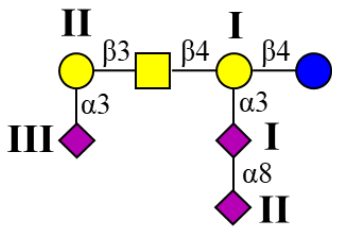

(GT1b)

To a solution of compound GD1b [36] (8.7 mg, 6.5 μmol), CMP-Neu5Ac (6.4 mg, 9.7 μmol) and $MgCl_2$ (20 mM) in Tris-HCl buffer (100 mM, pH=8.0) was added *Pp*α2.3siaT (1.6 mg/mL). Subsequently, the reaction was monitored using ESI every 2 h until optimal conversion is achieved. The reaction mixture was centrifuged, and the resulting supernatant was purified by size-exclusion chromatography (BioGel P-2, 45−90 μm, eluent: 0.1 M NH_4HCO_3). The fractions containing the product were combined and lyophilized to afford compound GT1b as a white amorphous solid (8.7 mg, 82%). ^{1}H NMR (600 MHz, D_2O) δ 4.74 (d, J = 8.4 Hz, 1H, H1-GalNAc), 4.60 (d, J = 7.8 Hz, 1H, H1-Gal-II), 4.53 – 4.49 (m, 2H, H1-Gal-I, H1-Glc), 4.21 – 4.14 (m, 3H), 4.13 – 4.07 (m, 2H), 4.07 – 3.97 (m, 4H), 3.96 – 3.88 (m, 4H), 3.88 – 3.80 (m, 8H), 3.77 (dd, J = 10.7, 3.1 Hz, 5H), 3.74 – 3.69 (m, 5H), 3.66 (dt, J = 5.7, 2.1 Hz, 5H), 3.63 – 3.59 (m, 7H), 3.55 (dd, J = 9.7, 7.8 Hz, 2H), 3.42 (dd, J = 9.9, 7.8 Hz, 1H), 3.32 (t, J = 8.4 Hz, 1H), 3.17 (t, J = 6.7 Hz, 2H), 2.79 – 2.71 (m, 3H,

H3(eq)-Neu5Ac-I, H3(eq)-Neu5Ac-II, H3(eq)-Neu5Ac-III), 2.07 (s, 3H, CH_3CONH), 2.06 – 1.98 (m, 11H, -CH_2-, 3 × CH_3CONH), 1.84 – 1.70 (m, 3H, H3(ax)-Neu5Ac-I, H3(ax)-Neu5Ac-II, H3(ax)-Neu5Ac-III). ESI-MS calcd for $C_{62}H_{101}N_5O_{45}^{2-}$ $[M-2H]^{2-}$ 817.79, found 817.95.

Compound GT1c

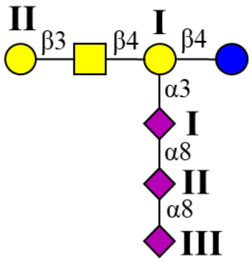

(GT1c)

To a solution of compound GT2 [36] (3.4 mg, 2.3 μmol), UDP-Gal (1.7 mg, 2.8 μmol) and $MgCl_2$ (10 mM) in Tris-HCl buffer (100 mM, pH=7.5) was added CgtB (0.5 mg/mL). Subsequently, the reaction was monitored using ESI every 2 h until optimal conversion is achieved. The reaction mixture was centrifuged, and the resulting supernatant was purified by size-exclusion chromatography (BioGel P-2, 45−90 μm, eluent: 0.1 M NH_4HCO_3). The fractions containing the product were combined and lyophilized to afford compound GT1c as a white amorphous solid (3.0 mg, 80%). ^{1}H NMR (600 MHz, D_2O) δ 4.74 (d, J = 8.8 Hz, 1H, H1-GalNAc), 4.54 – 4.48 (m, 3H, H1-Gal-II, H1-Gal-I, H1-Glc), 4.20 – 4.12 (m, 5H), 4.08 – 3.99 (m, 4H), 3.95 – 3.87 (m, 5H), 3.86 – 3.80 (m, 6H), 3.81 – 3.70 (m, 10H), 3.70 – 3.56 (m, 14H), 3.52 (dd, J = 9.8, 7.7 Hz, 1H), 3.40 (t, J = 9.0 Hz, 1H), 3.33 (t, J = 8.3 Hz, 1H), 3.16 (t, J = 7.0 Hz, 2H), 2.76 (dd, J = 12.4, 4.6 Hz, 1H, H3(eq)-Neu5Ac-III), 2.71 – 2.65 (m, 2H, H3(eq)-Neu5Ac-II, H3(eq)-Neu5Ac-I), 2.08 (s, 3H, CH_3CONH), 2.08 – 1.99 (m, 11H, -CH_2-, 3 × CH_3CONH), 1.82 – 1.66 (m, 3H, H3(ax)-Neu5Ac-II, H3(ax)-Neu5Ac-III, H3(ax)-Neu5Ac-I). ESI-MS calcd for $C_{62}H_{101}N_5O_{45}^{2-}$ $[M-2H]^{2-}$ 817.79, found 817.90.

Compound GQ1c

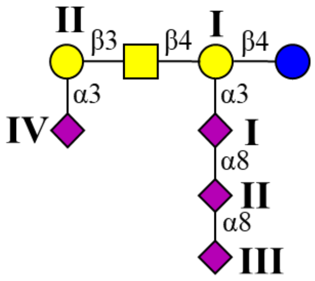

(GQ1c)

To a solution of compound GT1c (2.8 mg, 1.7 μmol), CMP-Neu5Ac (1.7 mg, 2.6 μmol) and $MgCl_2$ (20 mM) in Tris-HCl buffer (100 mM, pH=8.0) was added *Pp*α2.3siaT (1.6 mg/mL). Subsequently, the reaction was monitored using ESI every 2 h until optimal conversion is achieved. The reaction mixture was centrifuged, and the resulting supernatant was purified by size-exclusion chromatography (BioGel P-4, 45−90 μm, eluent: 0.1 M NH_4HCO_3). The fractions containing

the product were combined and lyophilized to afford compound GQ1c as a white amorphous solid (2.7 mg, 82%). ^{1}H NMR (600 MHz, D_2O) δ 4.71 (d, J = 8.5 Hz, 1H, H1-GalNAc), 4.60 (d, J = 7.9 Hz, 1H, H1-Gal-II), 4.53 – 4.49 (m, 2H, H1-Gal-I, H1-Glc), 4.19 (dd, J = 10.5, 2.8 Hz, 2H), 4.12 – 4.08 (m, 3H), 4.07 – 3.98 (m, 5H), 3.96 – 3.92 (m, 3H), 3.90 – 3.82 (m, 13H), 3.77 (t, J = 6.5 Hz, 8H), 3.73 – 3.68 (m, 9H), 3.67 – 3.64 (m, 3H), 3.63 – 3.60 (m, 3H), 3.57 (dd, J = 17.8, 9.1 Hz, 3H), 3.45 – 3.41 (m, 1H), 3.33 (t, J = 8.6 Hz, 1H), 3.17 (t, J = 6.9 Hz, 2H), 2.82 (dd, J = 12.6, 4.6 Hz, 1H, , $H3_{(eq)}$-Neu5Ac-IV), 2.78 – 2.71 (m, 3H, $H3_{(eq)}$-Neu5Ac-III, $H3_{(eq)}$-Neu5Ac-II, $H3_{(eq)}$-Neu5Ac-I), 2.08 – 2.01 (m, 17H, -CH_2-, 5 × CH_3CONH), 1.83 – 1.73 (m, 4H, $H3_{(ax)}$-Neu5Ac-I, $H3_{(ax)}$-Neu5Ac-II, $H3_{(ax)}$-Neu5Ac-III, $H3_{(ax)}$-Neu5Ac-IV). ESI-MS calcd for $C_{73}H_{118}N_6O_{53}^{2-}$ $[M-2H]^{2-}$ 963.34, found 963.70.

Compound GP1c

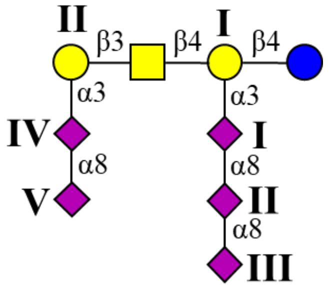

(GP1c)

To a solution of compound GQ1c (2.7 mg, 1.4 μmol), CMP-Neu5Ac (1.4 mg, 2.1 μmol) and $MgCl_2$ (20 mM) in Tris-HCl buffer (100 mM, pH=8.0) was Cst-II (0.1 mg/mL) were added. Subsequently, the reaction is monitored by ESI-MS every 1 h until optimal conversion was achieved. The reaction mixture was centrifuged, and the resulting supernatant was purified by size-exclusion chromatography (BioGel P-4, 45−90 μm, eluent: 0.1 M NH_4HCO_3). The fractions containing the product were combined and lyophilized to afford compound GP1c as a white amorphous solid (2.4 mg, 77%). ^{1}H NMR (600 MHz, D_2O) δ 4.72 (d, J = 8.1 Hz, 1H, H1-GalNAc), 4.61 (d, J = 8.0 Hz, 1H, H1-Gal-II), 4.52 (d, J = 8.0 Hz, 1H, H1-Gal-I), 4.49 (d, J = 7.9 Hz, 1H, H1-Glc), 4.22 – 4.11 (m, 7H), 4.12 – 4.02 (m, 5H), 4.03 – 3.96 (m, 2H), 3.97 – 3.88 (m, 6H), 3.88 – 3.81 (m, 9H), 3.83 – 3.77 (m, 4H), 3.78 – 3.71 (m, 4H), 3.69 (q, J = 4.5 Hz, 6H), 3.69 – 3.64 (m, 5H), 3.66 – 3.61 (m, 8H), 3.61 – 3.56 (m, 3H), 3.43 (dd, J = 9.9, 7.8 Hz, 1H), 3.33 (t, J = 8.5 Hz, 1H), 3.18 – 3.14 (m, 2H), 2.79 – 2.75 (m, 3H, $H3_{(eq)}$-Neu5Ac-II, $H3_{(eq)}$-Neu5Ac-III, $H3_{(eq)}$-Neu5Ac-V), 2.71 – 2.65 (m, 2H, $H3_{(eq)}$-Neu5Ac-I, $H3_{(eq)}$-Neu5Ac-IV), 2.13 – 1.96 (m, 20H, -CH_2-, 6 × CH_3CONH), 1.80 – 1.68 (m, 5H, $H3_{(ax)}$-Neu5Ac-I, $H3_{(ax)}$-Neu5Ac-II, $H3_{(ax)}$-Neu5Ac-III, $H3_{(ax)}$-Neu5Ac-IV, $H3_{(ax)}$-Neu5Ac-V). ESI-MS calcd for $C_{84}H_{135}N_7O_{61}^{2-}$ $[M-2H]^{2-}$ 1108.89, found 1109.10.

(3) NMR Spectra

^{1}H NMR (600 M, D_2O) of compound GT1a

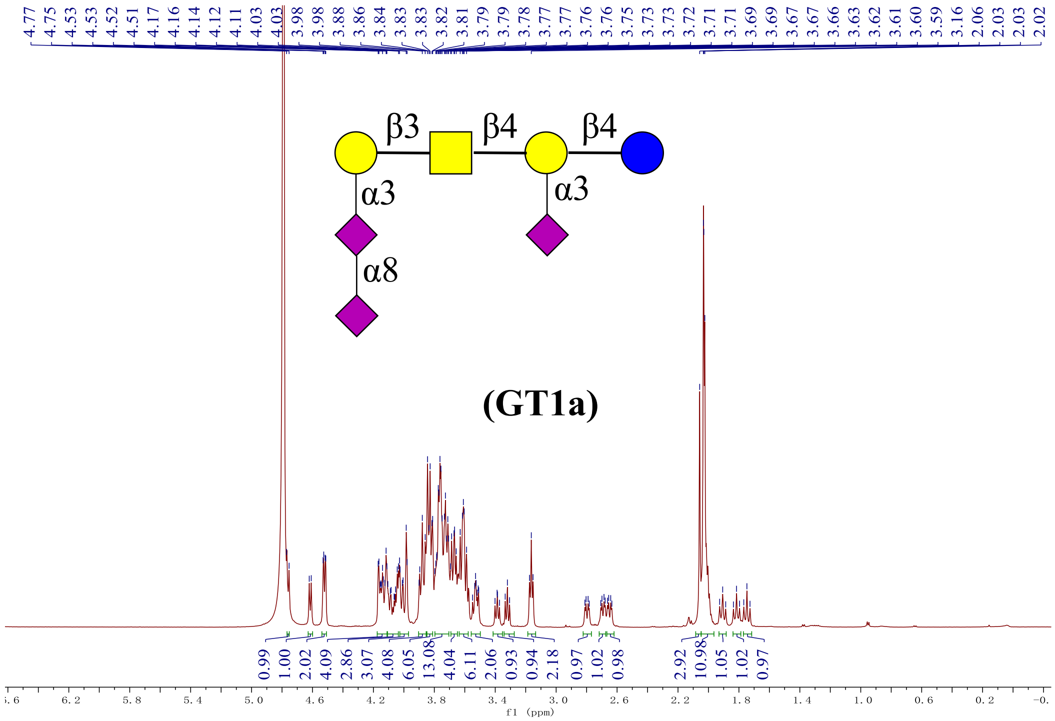

COSY of compound GT1a

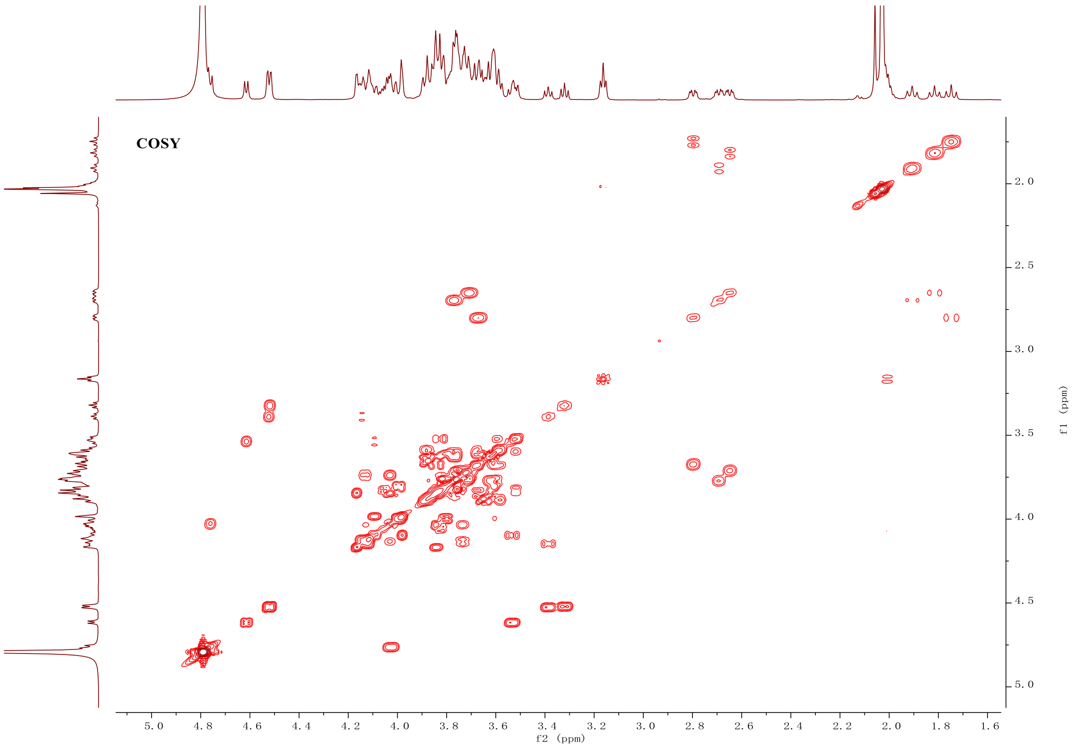

HSQC of compound GT1a

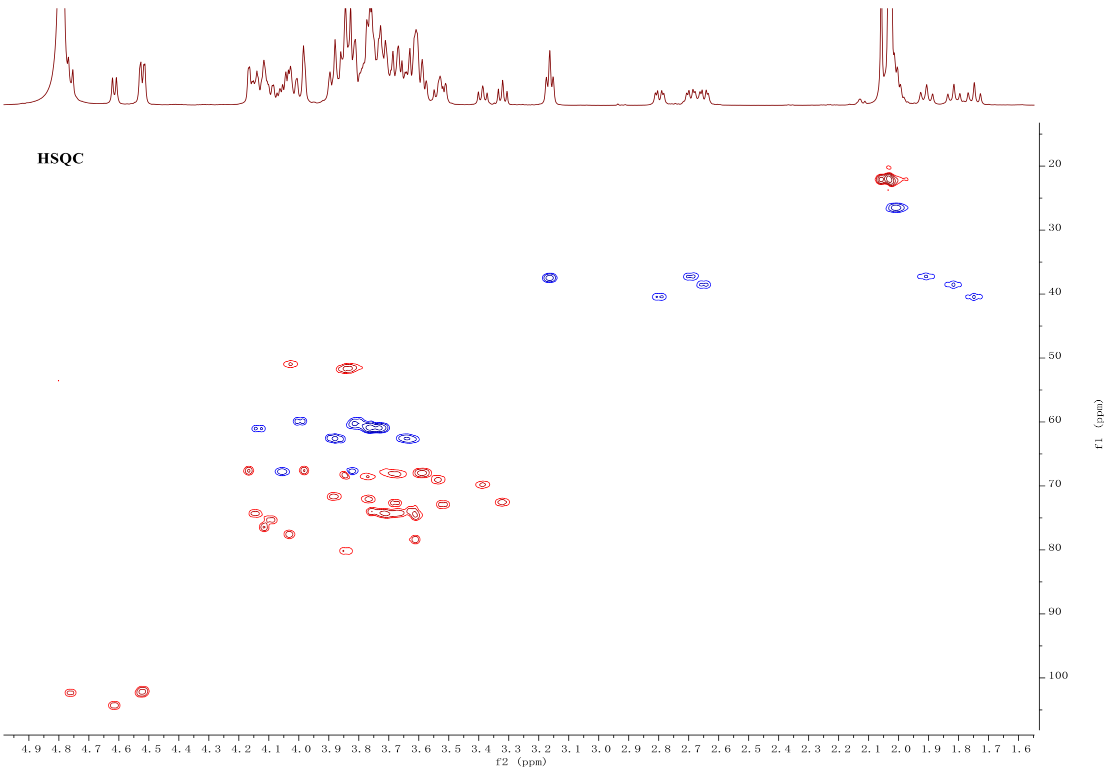

TOCSY of compound GT1a

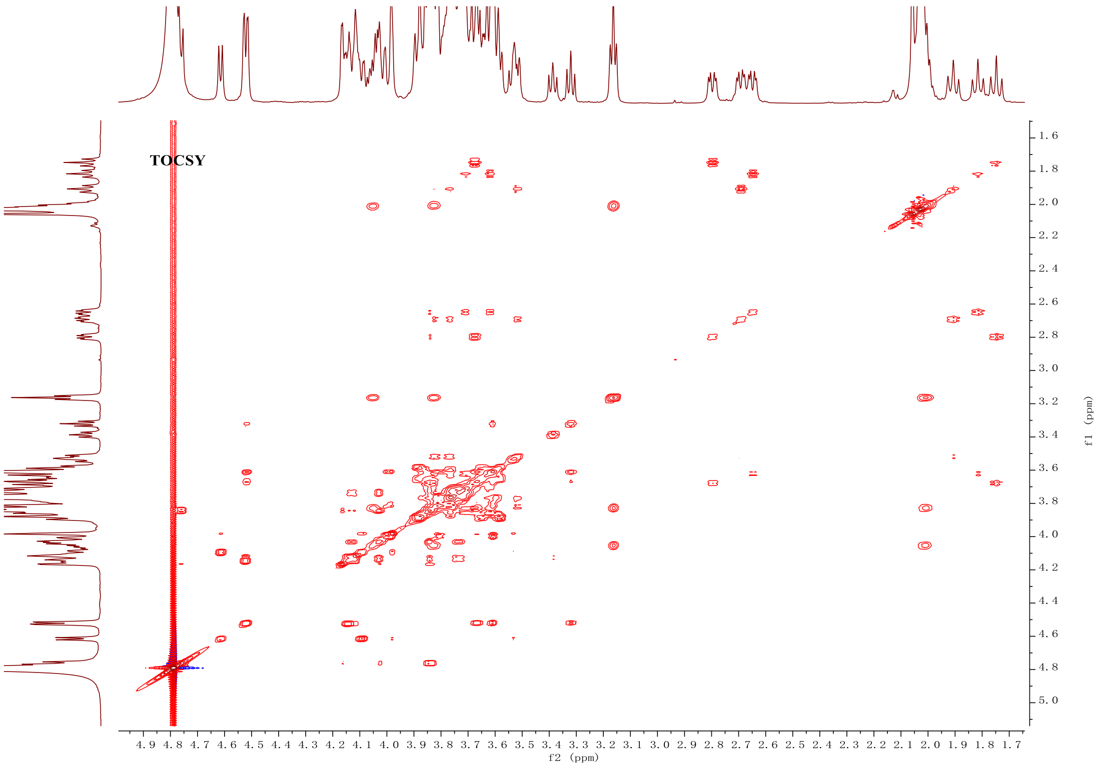

^{1}H NMR (600 M, D_2O) of compound GT1aα

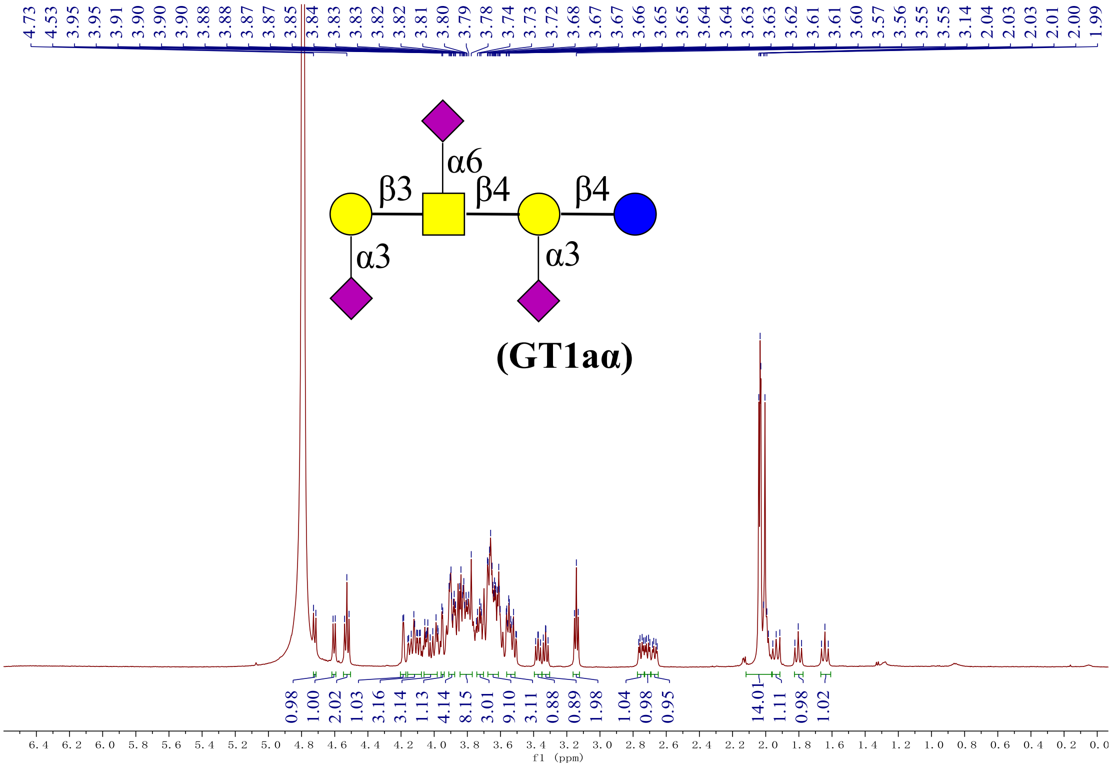

COSY of compound GT1aα

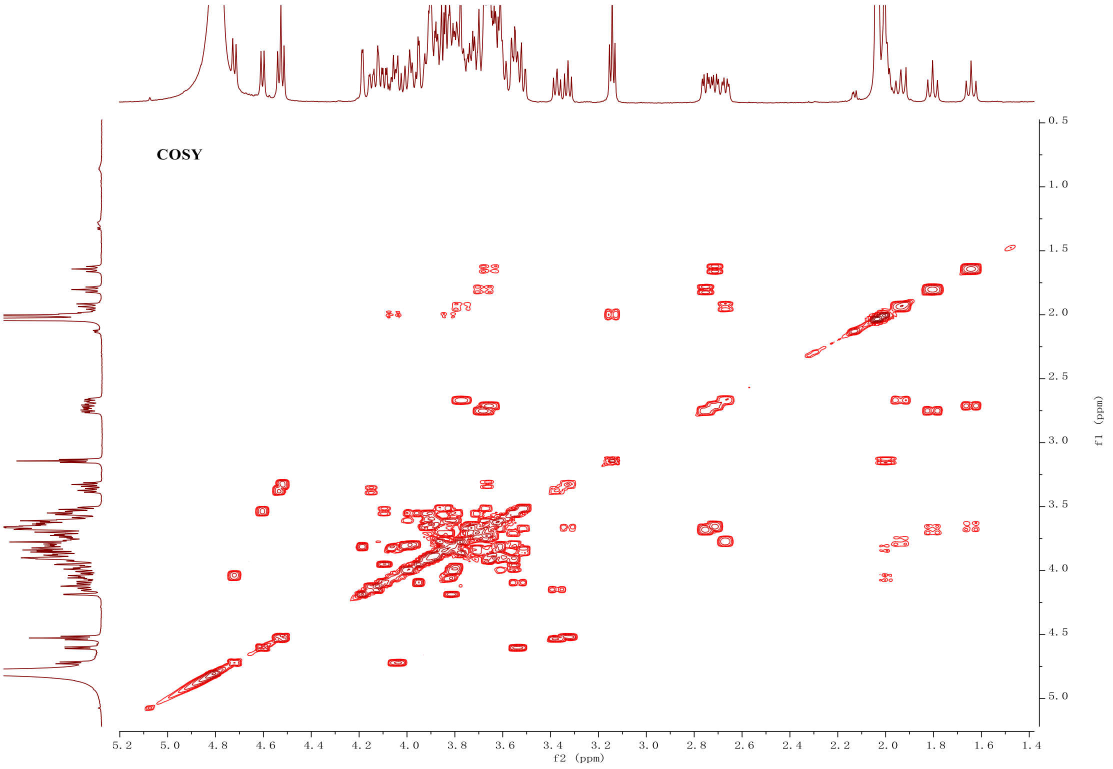

HSQC of compound GT1aα

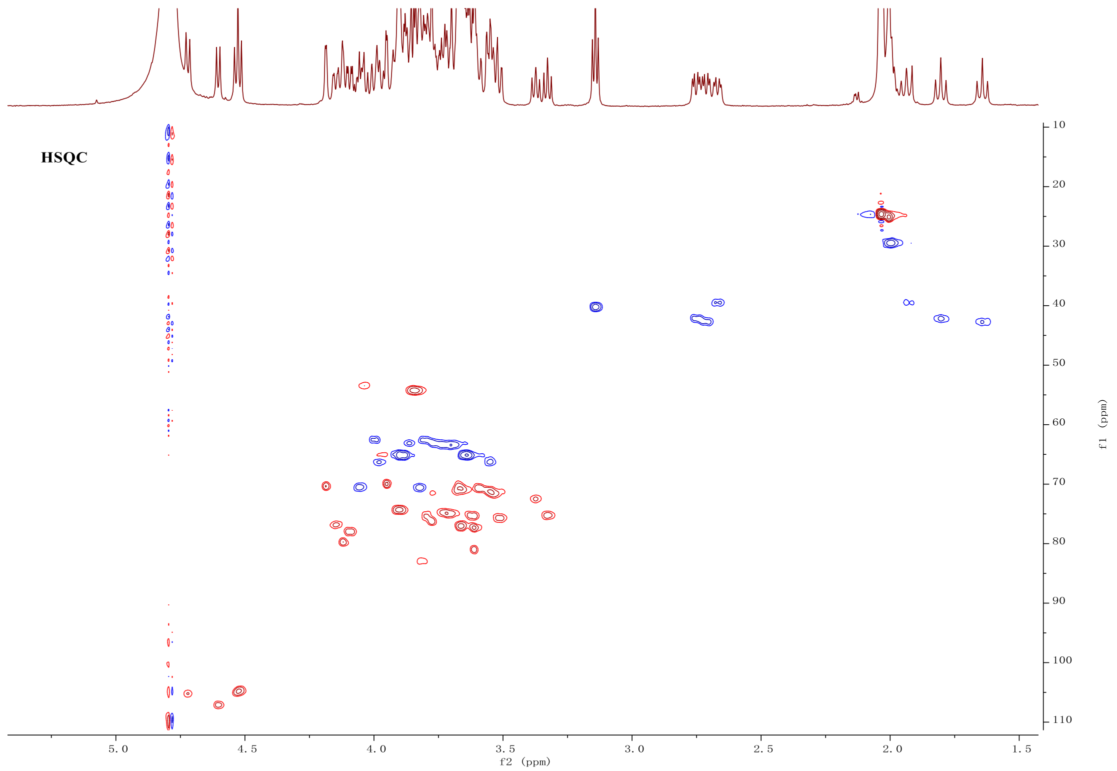

TOCSY of compound GT1aα

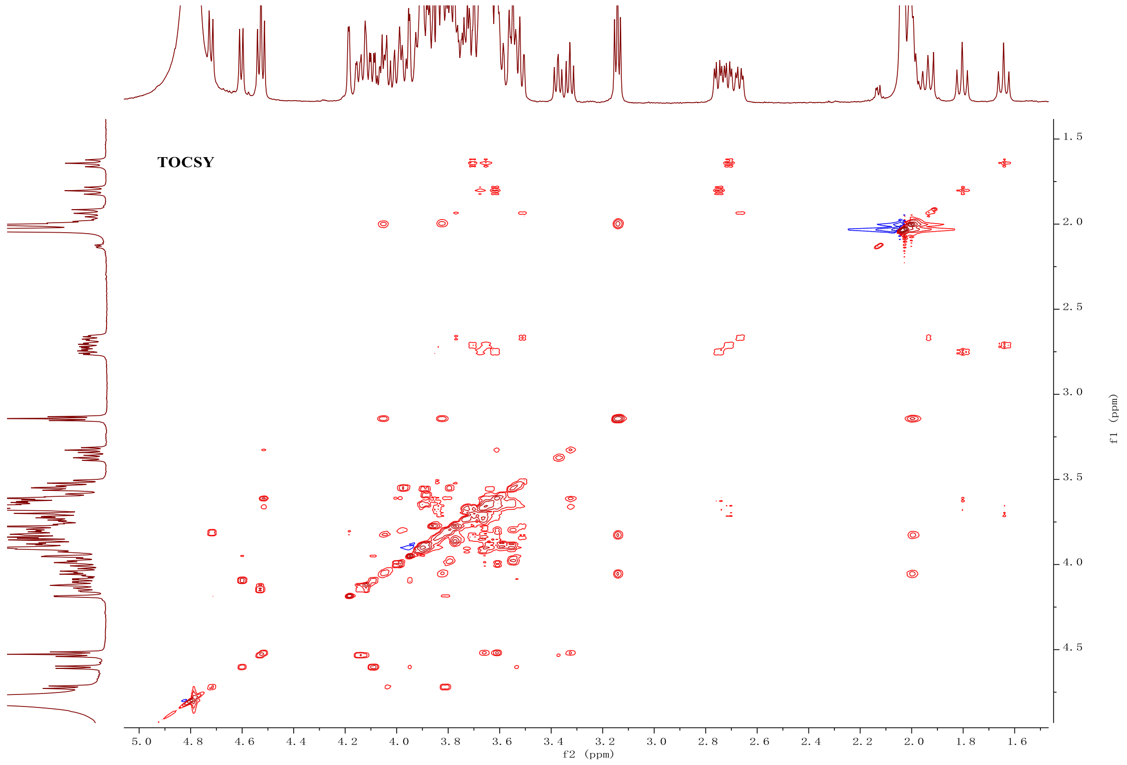

^{1}H NMR (600 M, D_2O) of compound GT1b

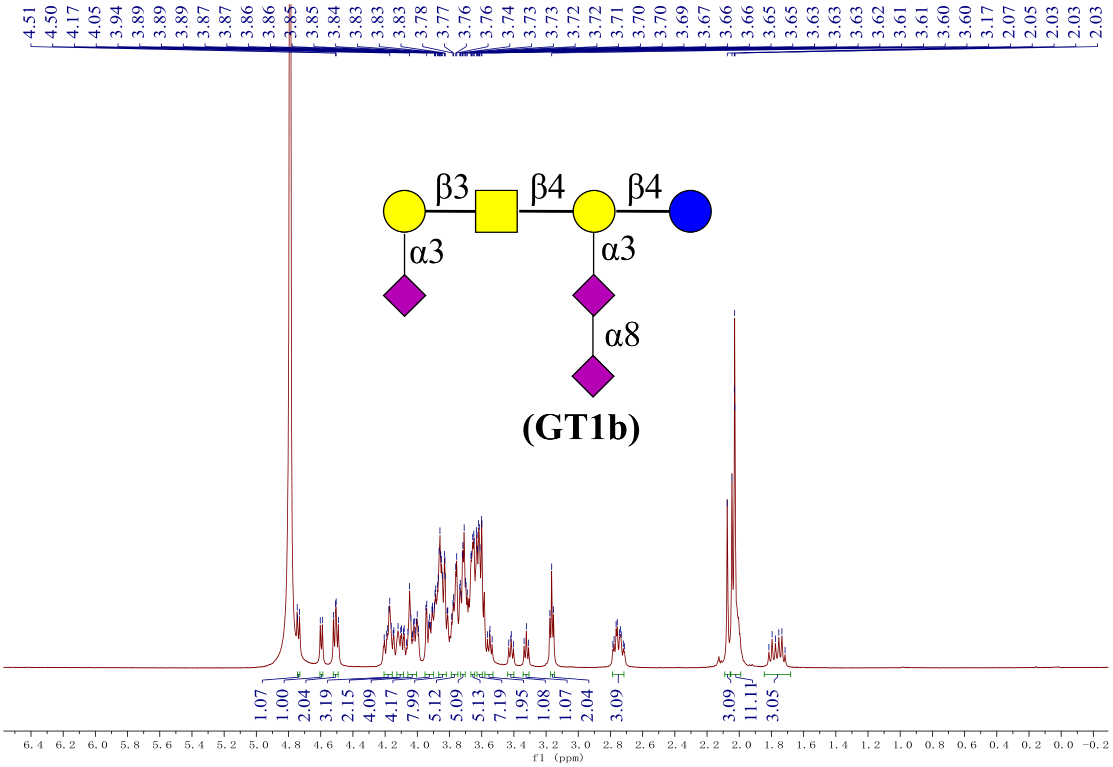

COSY of compound GT1b

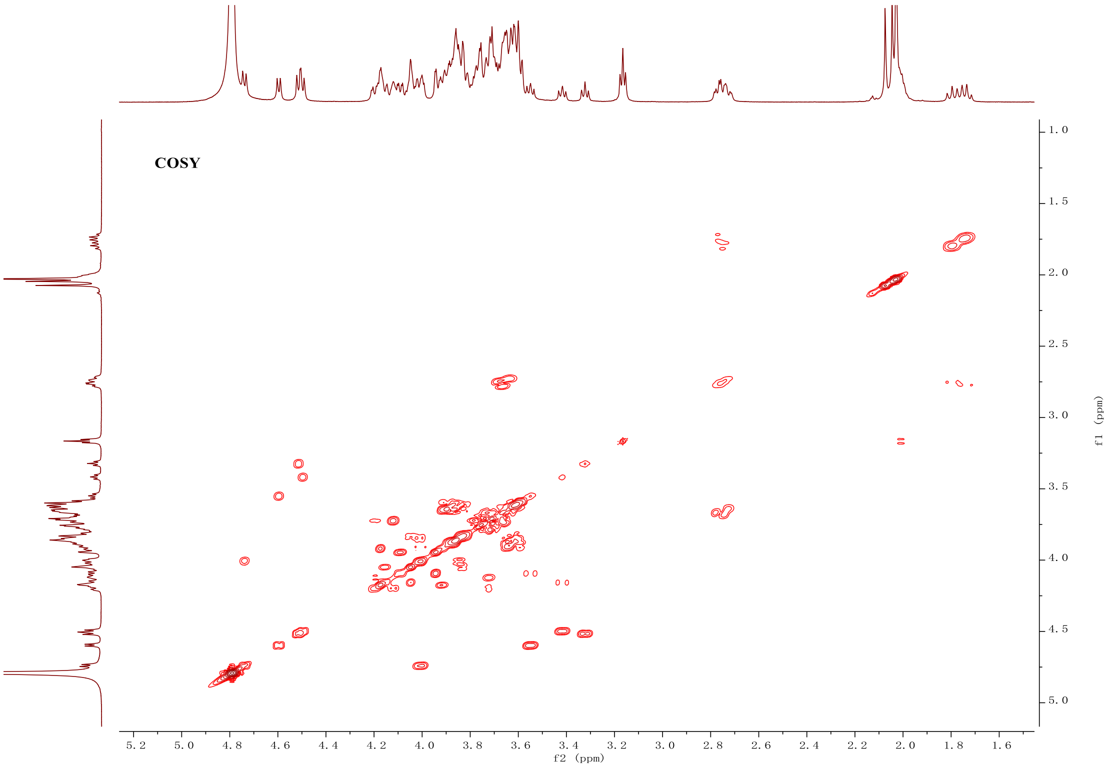

HSQC of compound GT1b

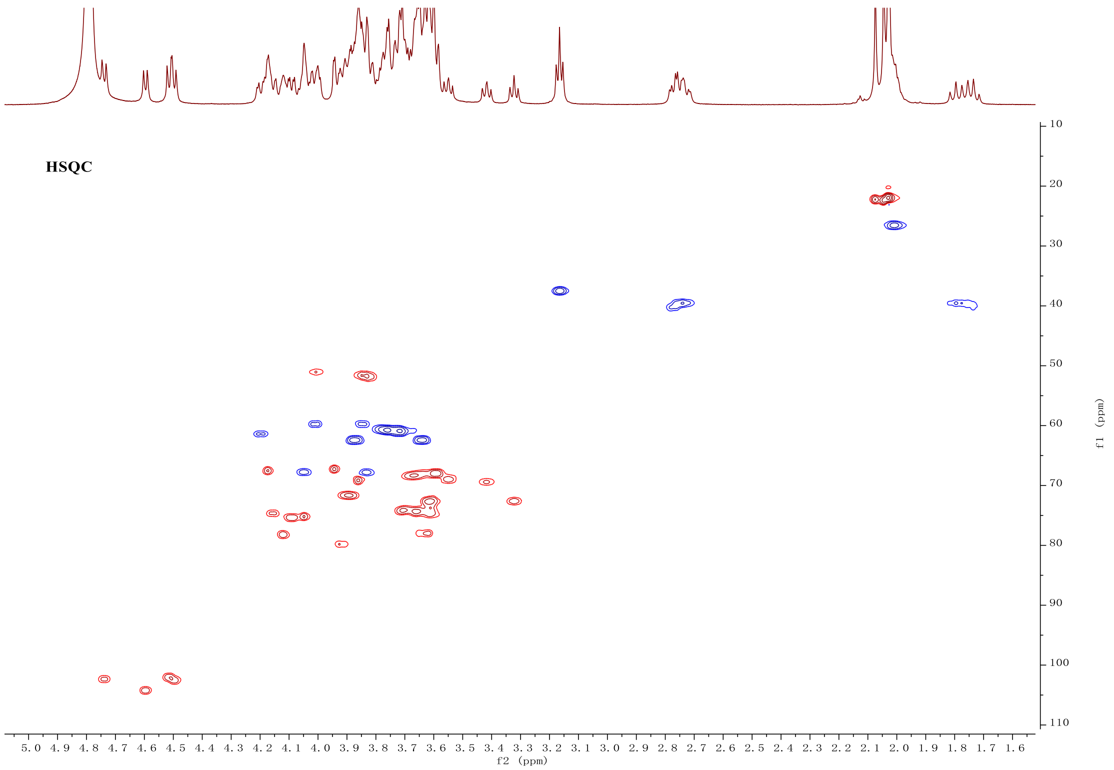

TOCSY of compound GT1b

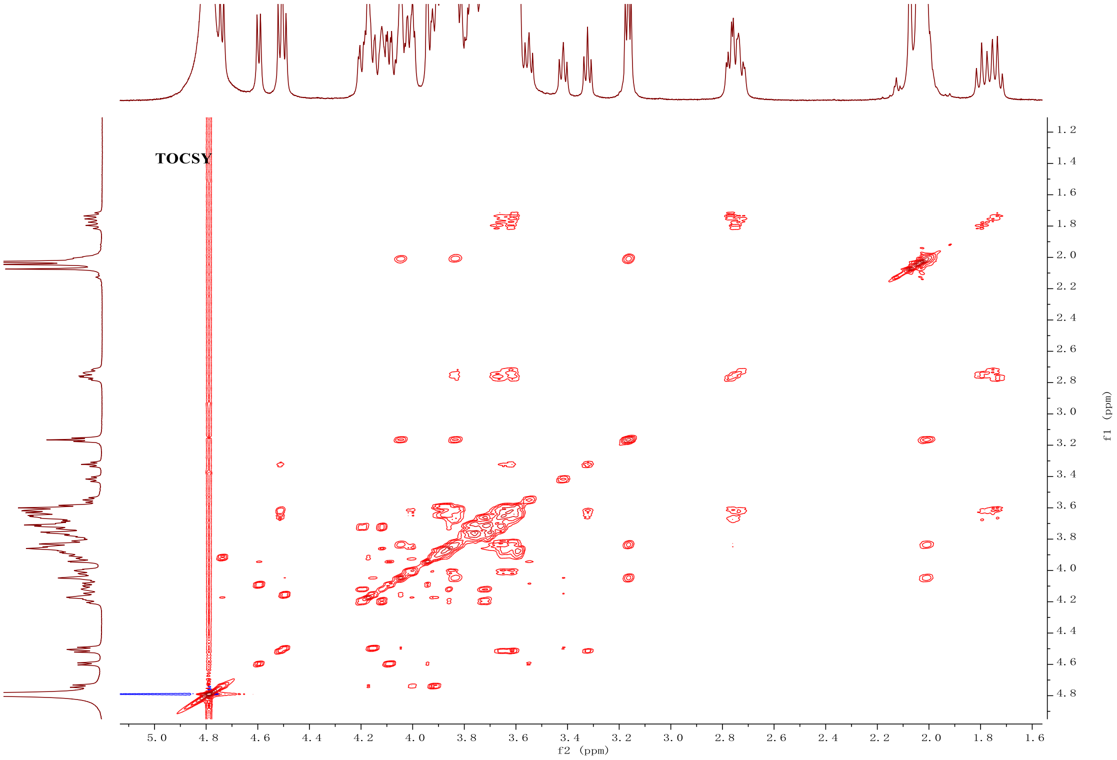

^{1}H NMR (600 M, D_2O) of compound GT1c

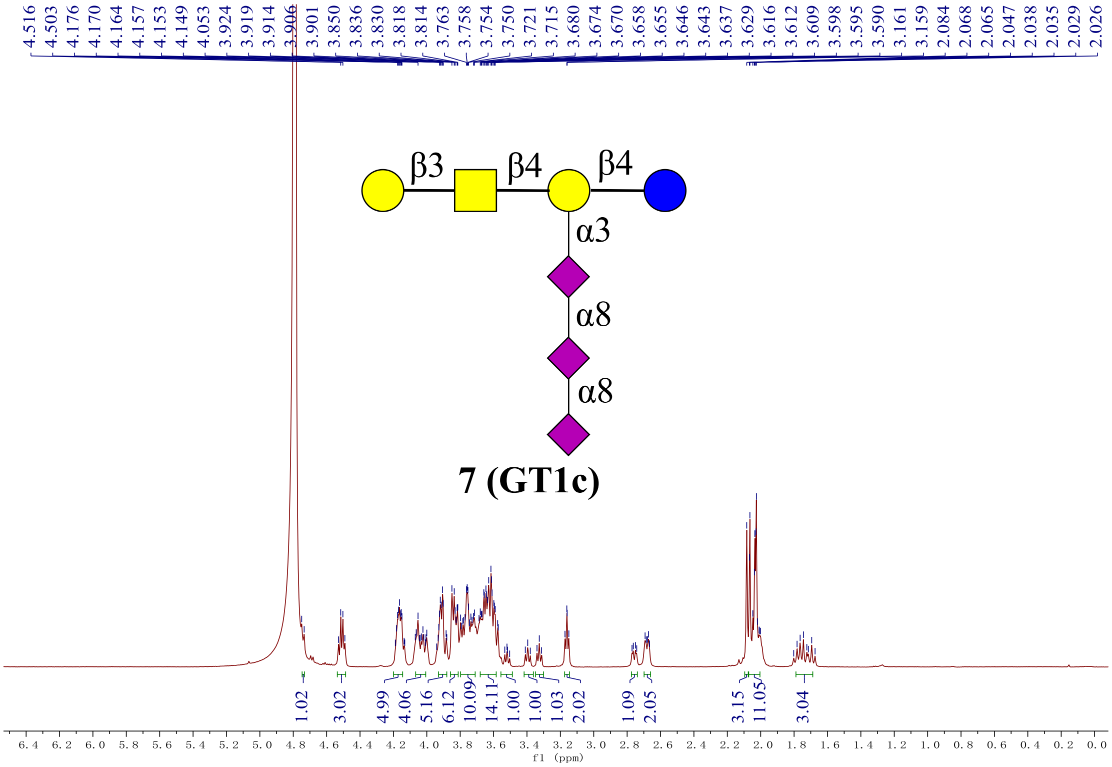

COSY of compound GT1c

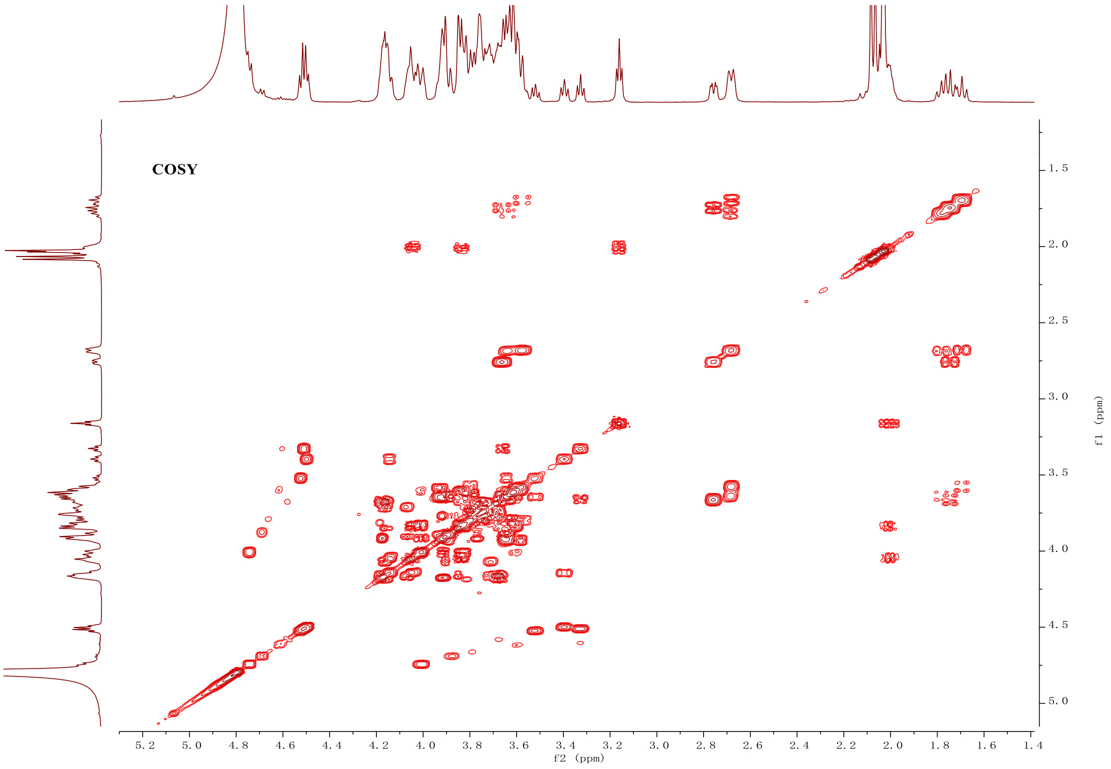

HSQC of compound GT1c

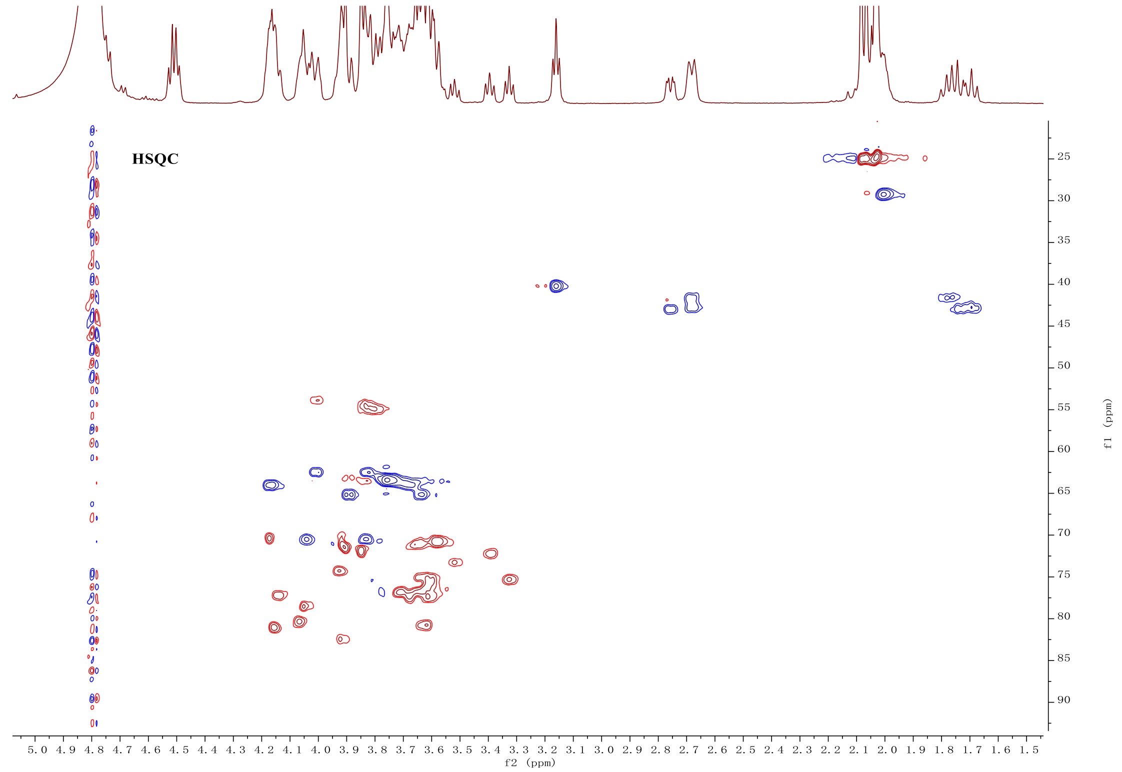

TOCSY of compound GT1c

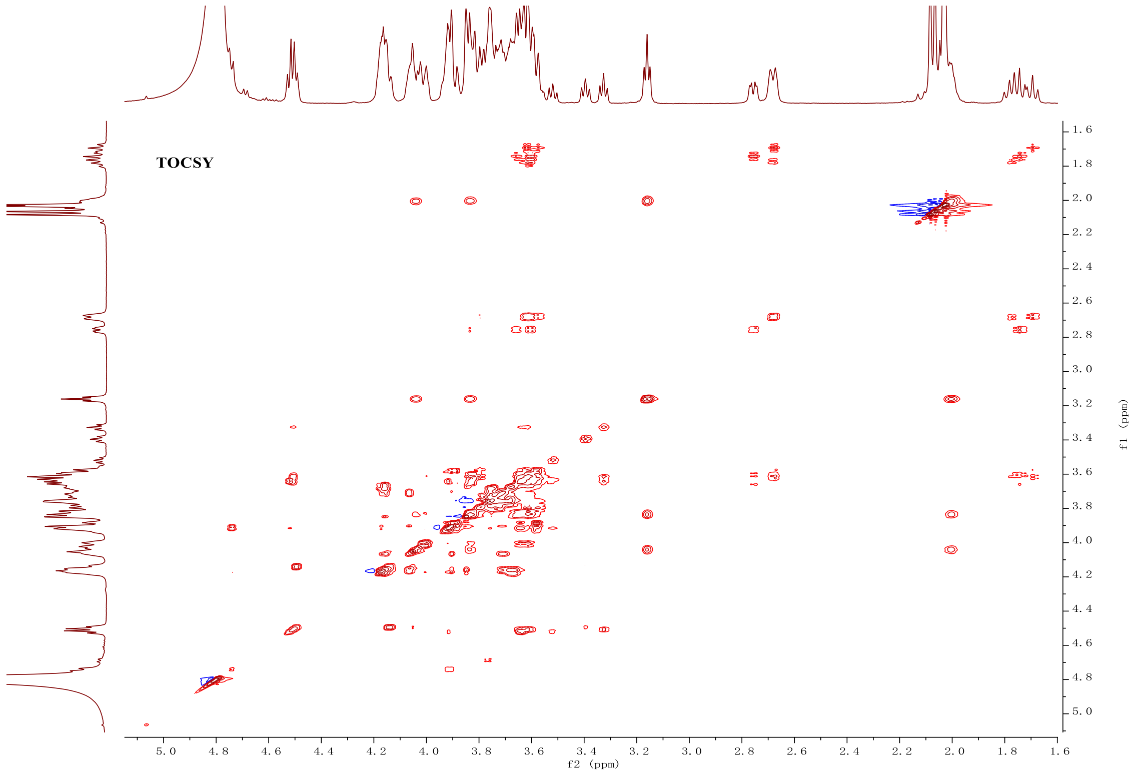

^{1}H NMR (600 M, D_2O) of compound GQ1c

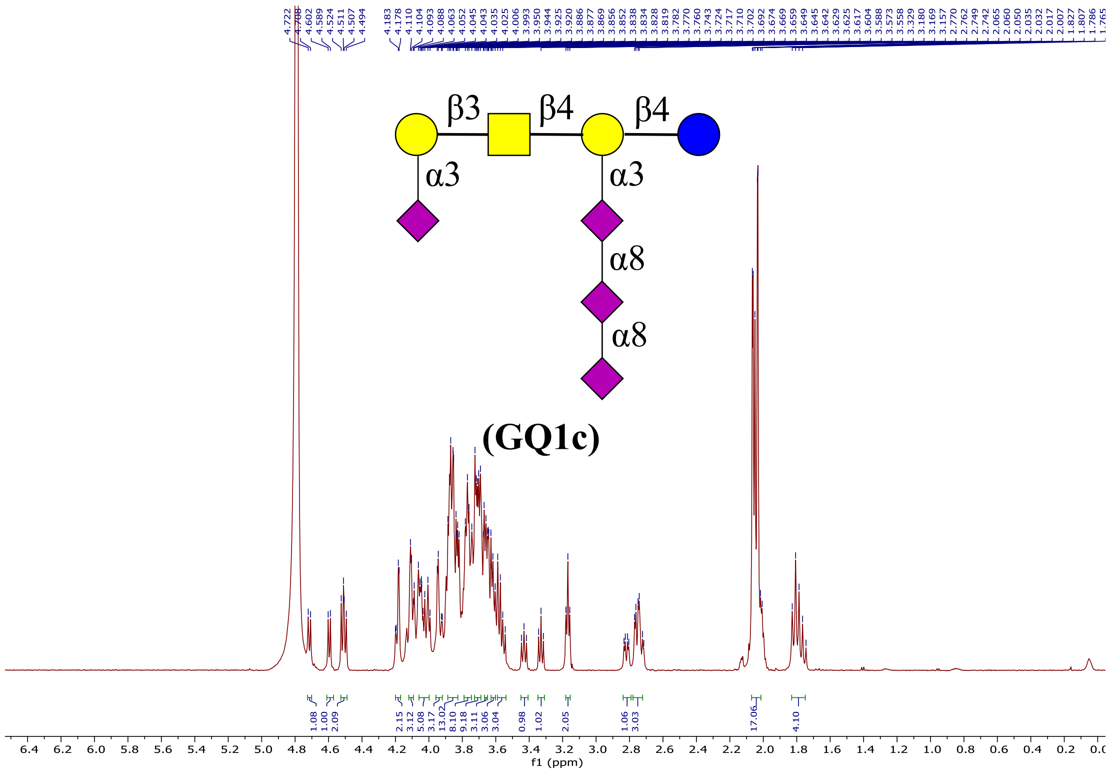

COSY of compound GQ1c

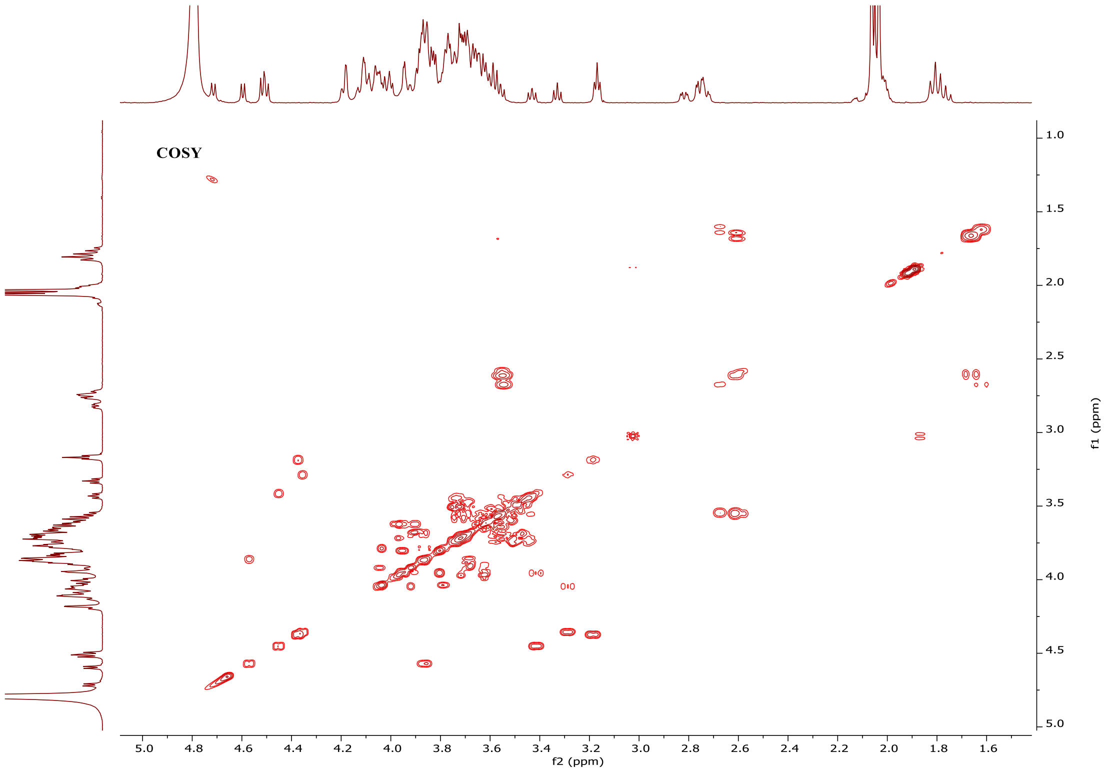

HSQC of compound GQ1c

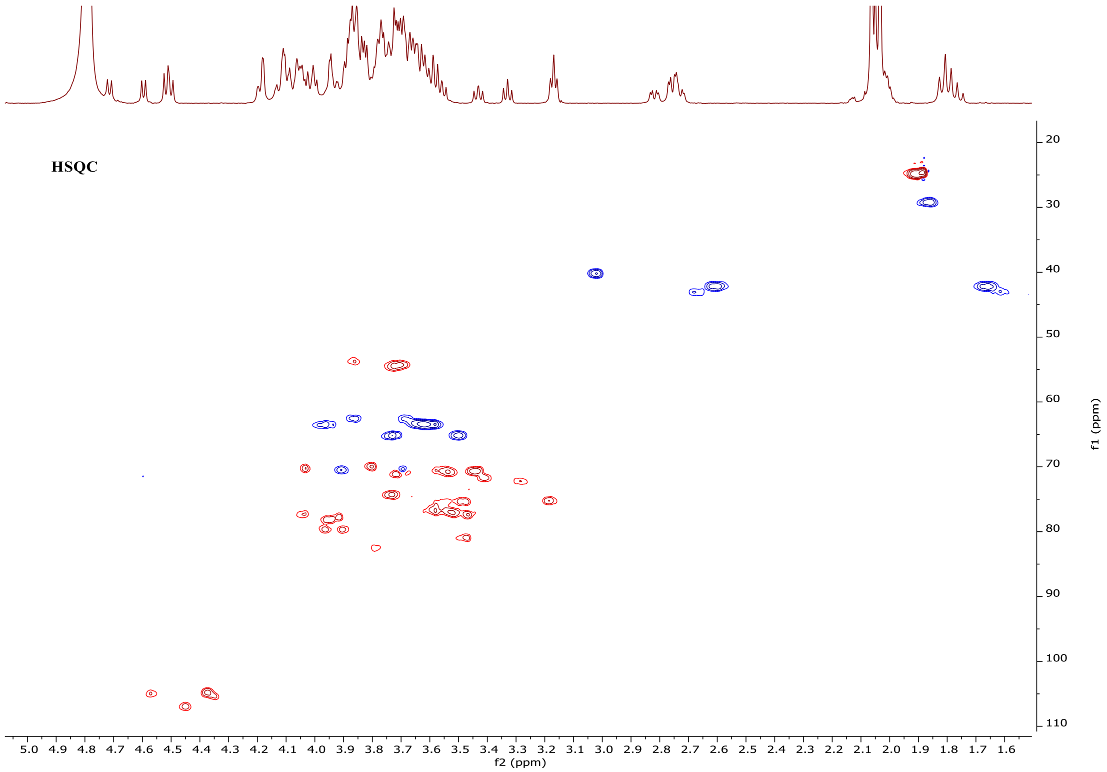

TOCSY of compound GQ1c

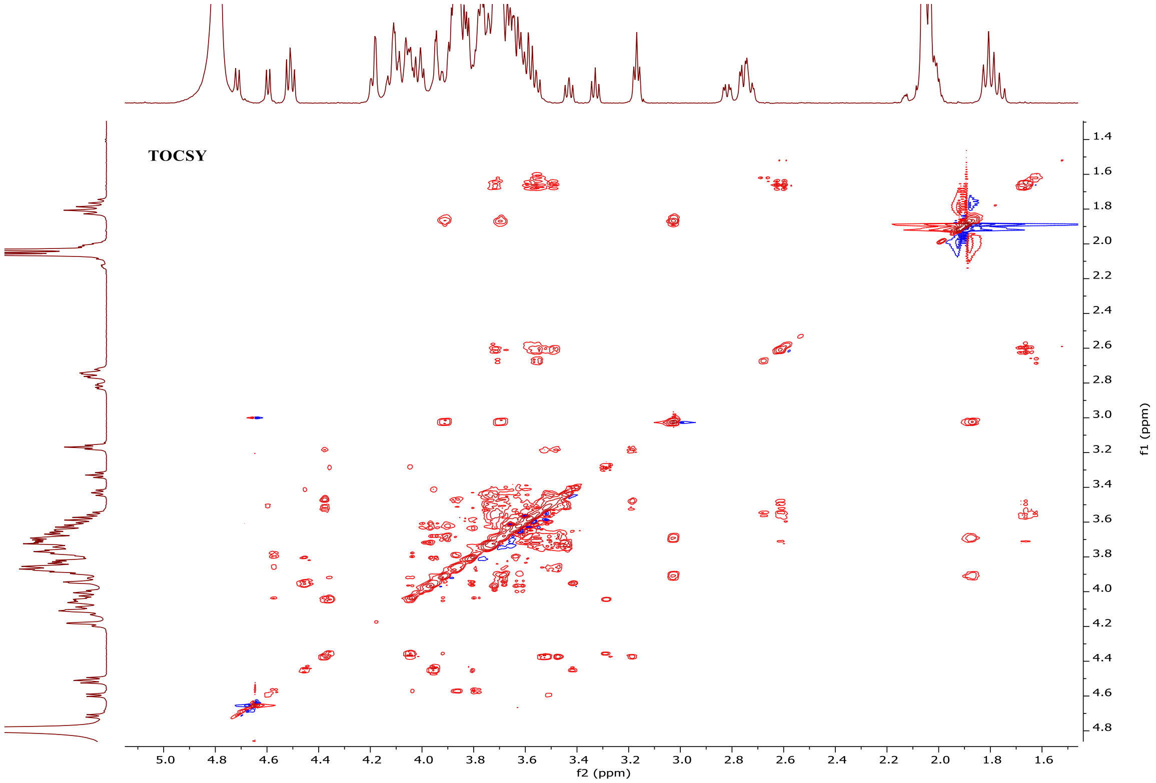

^{1}H NMR (600 M, D_2O) of compound GP1c

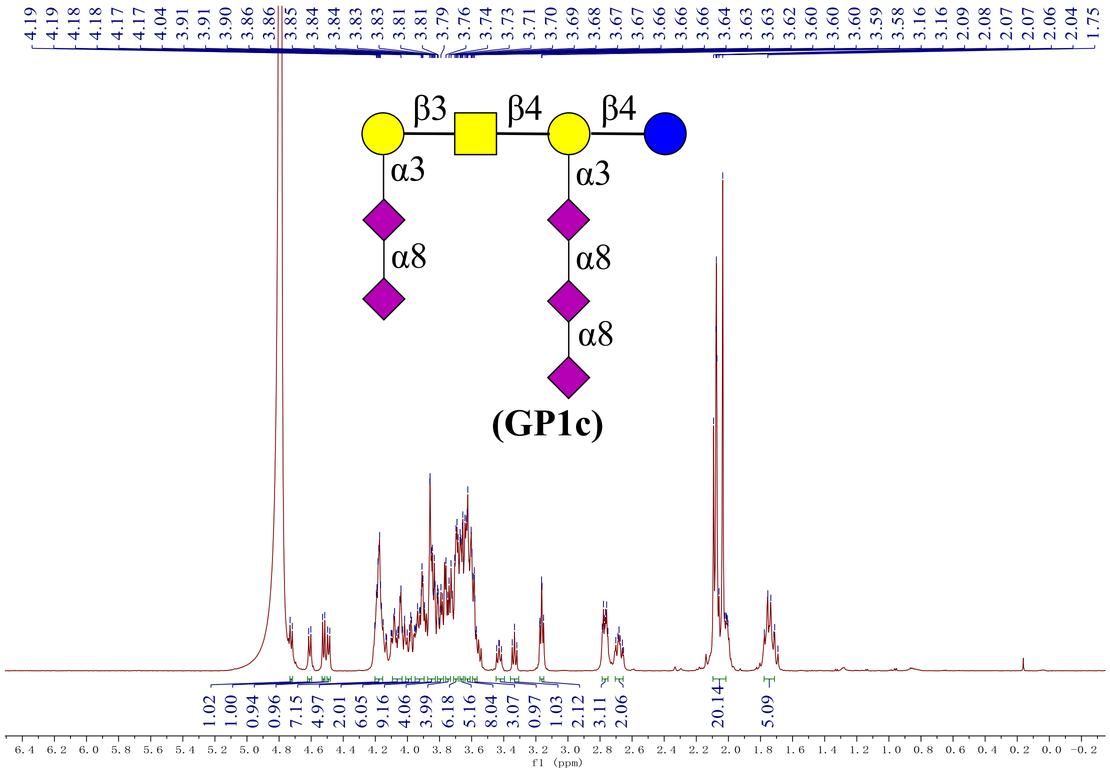

COSY of compound GP1c

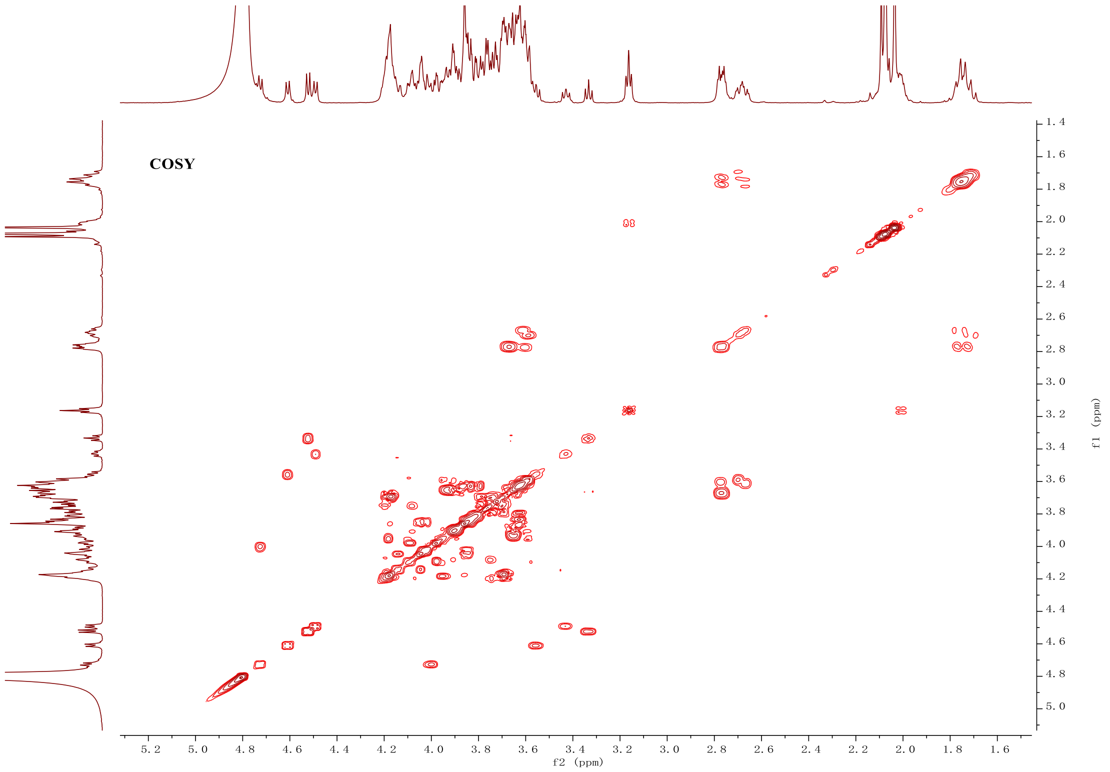

HSQC of compound GP1c

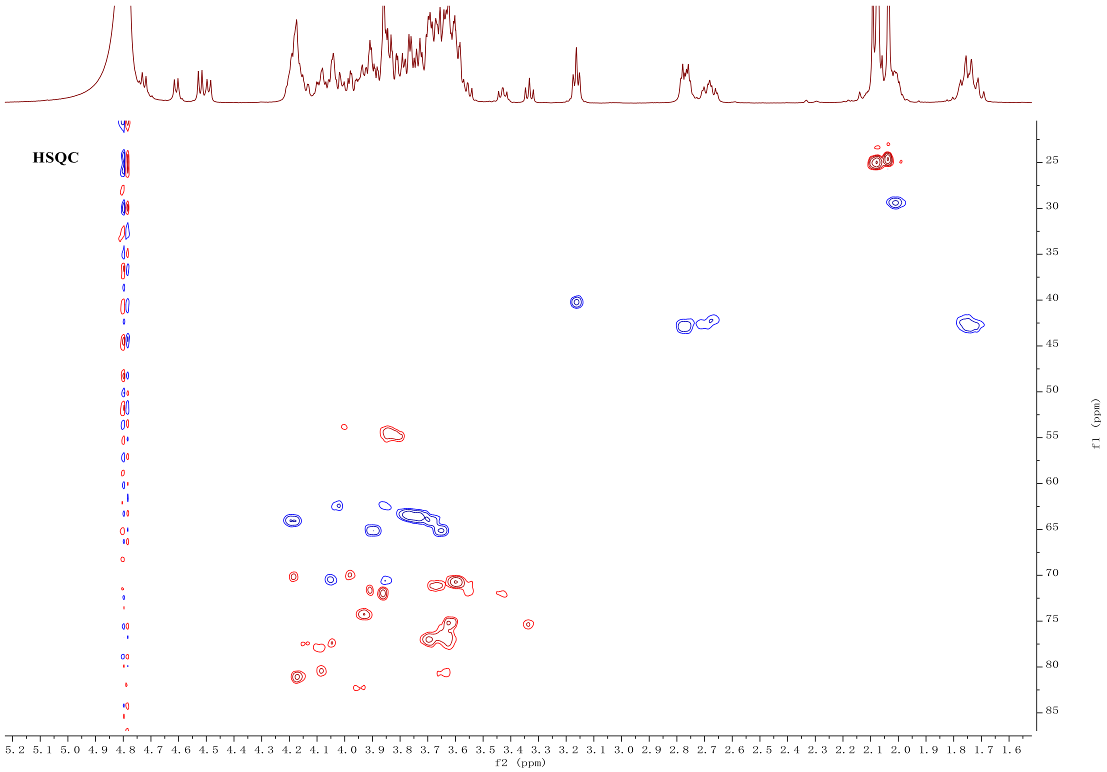

TOCSY of compound GP1c

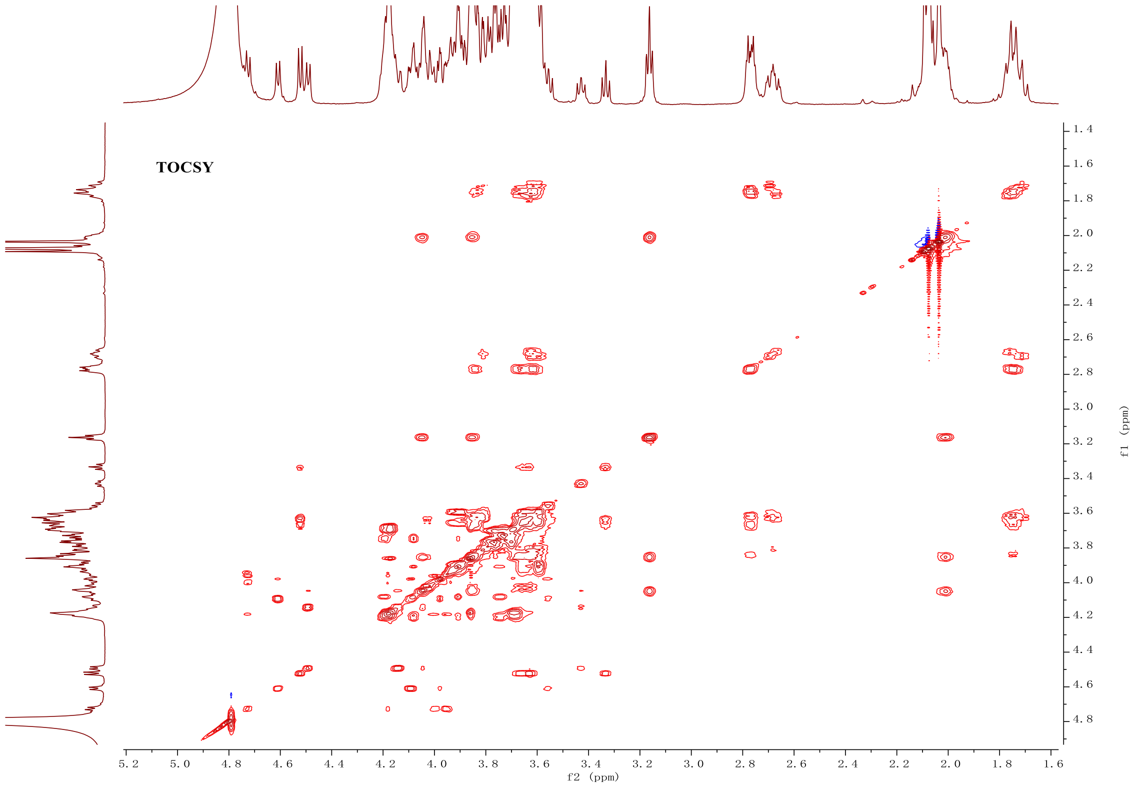

Supplement: Supplementary 1 — Figs. S1 to S25 Tables S1 to S6 [file research.1286.f1.pdf]
